# Country Contextualization of the Mental Health Gap Action Programme Intervention Guide: A Case Study from Nigeria

**DOI:** 10.1371/journal.pmed.1001501

**Published:** 2013-08-20

**Authors:** Jibril Abdulmalik, Lola Kola, Woye Fadahunsi, Kazeem Adebayo, M. Taghi Yasamy, Emmanuel Musa, Oye Gureje

**Affiliations:** 1 WHO Collaborating Centre for Research and Training in Mental Health and Neuroscience, Department of Psychiatry, University of Ibadan, Nigeria; 2 World Health Organization, Nigeria Country Office, Osogbo, Nigeria; 3 Department of Psychiatry, Ladoke Akintola University of Technology, Ogbomoso, Nigeria; 4 Department of Mental Health and Substance Abuse, World Health Organization, Geneva, Switzerland; 5 Nigeria Country Office, World Health Organization, Abuja, Nigeria

## Abstract

As one article in an ongoing series on Global Mental Health Practice, Jibril Abdulmalik and colleagues describe the implementation of the mhGAP-Intervention Guide in Nigeria, which has resulted in a country-specific version.

*Please see later in the article for the Editors' Summary*

**Summary Points**

- The challenge of unmet need for mental health services in low- and middle-income countries is huge and calls for innovative approaches to improve coverage and access to these services.
- A viable option for attaining these objectives is the integration of mental health into primary health care though a system of skill-transfer and support from specialists. The Mental Health Gap Action Programme-Intervention Guide (mhGAP-IG) is a useful manual for aiding non-specialists to provide mental health services. It was produced by the World Health Organization to help countries scale up mental health care services.
- The mhGAP-IG provides a generic template that requires adaptation and contextualization to suit the particular needs of the health system in a given country. This process has now been successfully carried out in Nigeria, culminating in the production of a country-specific version of the mhGAP-IG.
- The process of contextualization and adaptation of the mhGAP-IG for Nigeria, and the lessons learnt during this process are presented here.

*This case study is part of the* PLOS Medicine *series on Global Mental Health Practice*.

## Introduction

The global burden of disease (GBD) study clearly brought to the fore the societal impact of mental health conditions, with the finding that neuropsychiatric conditions account for about 14% of the total disease burden [1]. The extent of this burden has been further emphasized by recent large-scale global surveys, which indicate that not only are mental disorders highly prevalent worldwide, but also that a huge treatment gap exists in both developed and developing countries [2],[3]. Furthermore, there is evidence to demonstrate that effective and affordable treatment for common mental disorders is feasible, even in low- and middle-income countries (LMICs) [4]. Several barriers and constraints in these countries have led to a situation where those with the greatest need for mental health services often lack access to these services [5]. It has therefore become imperative to seek innovative solutions to these global mental health challenges [6]–[8]. Current evidence favours innovative solutions that would result in overall health system strengthening, integrated provision of services [9], and improved access to evidence-based packages of care through task-shifting [10]

As a way of confronting this global challenge, the World Health Organization (WHO) recently launched a programme of action to help countries implement activities aimed at narrowing the gap between need and available services for mental health care. This programme, the Mental Health Gap Action Programme (mhGAP), is designed to assist LMICs in their efforts to scale up the coverage of mental health services for their citizens [11]. An important component of the programme is the development of the mhGAP-Intervention Guide (mhGAP-IG), a manual designed to facilitate the recognition and management of a set of priority mental, neurological, and substance use (MNS) disorders in non-specialist settings. The manual is the product of a rigorous international expert consensus on the most burdensome MNS conditions, and describes approaches to their recognition and the provision of evidence-based interventions, both pharmacological and non-pharmacological, for alleviating these conditions. The priority conditions covered by the mhGAP-IG are depression, psychosis, dementia, bipolar disorder, epilepsy, behavioural disorders, developmental disorders, alcohol use disorders, drug use disorders, and suicide and self harm. The guide provides information on the diagnosis of each condition and the common interventions that can be offered to sufferers by non-specialists. Particular emphasis is placed on the use of commonly available non-pharmacological interventions wherever evidence exists for their efficacy. Referrals to the next level of care are suggested when such are indicated. The process of developing this tool was based on the Grading of Recommendations Assessment, Development and Evaluation (GRADE) methodology to generate an evidence-based, intervention package [12],[13]. Furthermore, the mhGAP-IG is not a stand-alone tool, but comes with a range of supporting technical tools, which includes a facilitator's guide for each module, a contextualization questionnaire, and tools for monitoring and evaluating its use.

There is wide variability in local circumstances among countries, both in regard to the organization of their health systems and the availability of resources to deliver the recommended interventions. Furthermore, differences also exist in the composition and training of health care personnel, loosely referred to as “non-specialists.” For example, in some countries these may be highly skilled doctors with postgraduate training in family medicine or general practice while in some others, especially in low resource settings, such providers may be community health workers with minimal training. For these reasons, it is recommended that a process of contextualization of the generic version of the mhGAP-IG be implemented in each country setting in order to produce a fully adapted version that meets with the needs of the extant health system in which it is to be used. This paper describes the process of adaptation and contextualization of the manual for the Nigerian health system, a system with broad similarities with those of many sub-Saharan African countries.

## The Nigerian Context

Nigeria is Africa's most populous nation, with an estimated population of about 150 million people. Community surveys show lifetime prevalence of mental disorders ranging from 12.1% to 26.2% [14]. Nigeria also participated in the multi-site World Mental Health Surveys, which revealed a huge treatment gap in both developed and developing countries. In Nigeria, only 20% of people with serious common mental disorders received treatment in the preceding 12 months, and this treatment was mostly below the standards for minimally adequate care [3]. The country is classified as an LMIC, and similar to what occurs in other LMICs, mental health services are poorly resourced and therefore difficult to access by the great majority of persons in need. For example, there is no clearly defined budget allocation for mental health in the national health budget even though estimates show that just about 3.3% of the annual health budget is spent on large, institution-based services provided through eight stand-alone mental hospitals [15]. These specialist mental hospitals are disproportionately distributed across the country, and are all located in major urban cities. The practical reality of inadequate access to mental health services for the majority of Nigerians, as a reflection of intra-country disparities, is best illustrated with an example from a region. The specialist neuropsychiatric facility located in the north-eastern region of the country is the only health facility that provides mental health services to the 18.9 million people who live in the six states that make up the region.

Nationally, the staff to population ratios per 100,000 population, of 0.06 psychiatrists, 0.02 psychologists, 0.19 nurses, and 0.09 non-specialized doctors for the country [16], make it clear that a pragmatic option for scaling up mental health services will be to integrate them into non-specialist services, especially into primary care, via a task-shifting approach [17]–[20]. This approach is also supported by the fairly uniform nationwide structure of the primary health care (PHC) system, which can result in greater and equitable improvement in coverage and access to mental health services. The intra-country disparity in the availability of mental health services is most acutely felt, in terms of the distribution of tertiary facilities and availability of mental health professionals. However, these disparities can be attenuated by the integration of mental health into the PHC system.

This option was duly recognized and recommended as the national strategy in the country's Mental Health Policy document of 1991 (see Box 1) [21]. However, implementation of the goals enunciated in that policy has been handicapped by the absence of a clear implementation plan. There has also been an absence of consistent efforts to influence the process of training primary care workers to improve their recognition and management of mental health conditions. The successful contextualization and pilot implementation of the mhGAP-IG as a tool for training primary care workers, therefore, presents an opportunity for the country to develop a pragmatic plan for scaling up mental health services through its integration into primary care. There is emerging evidence from similar projects, which are also leveraging the existing health system to improve mental health services provision in primary care [22] and through maternal mental health programmes [23].

**Box 1. The Summary Declarations of the Mental Health Policy of Nigeria (FMoH, 1991)**

- The policy will be based on the principles of social justice and equity.
- Individuals with mental, neurological, and psychosocial disorders have the same rights to treatment as individuals with physical illnesses.
- Mental health care will be integrated into general health care services at all levels.
- Comprehensive access to mental health services will be ensured through primary health care.
- Appropriate training of mental health care personnel will be provided.
- Fostering of inter-sectoral collaboration will be enhanced with the aim of improving quality of life.
- Healthy attitudes and positive socio-cultural attributes will be promoted.
- Stigma towards mental illness will be eliminated through the promotion of positive attitudes towards the mentally ill in the population.
- The reduction of alcohol and drug abuse, utilizing appropriate preventive, therapeutic and rehabilitative measures will be pursued.
- Special care will be provided to the disadvantaged minority groups in the community.
- Encouragement of non-governmental organizations (NGOs) to participate in various aspects of mental health services
- Full collaboration with appropriate international organizations.
- Periodic review of mental health legislation.
- Encouragement and funding for mental health research.

After a series of technical discussions between the representatives of the Federal Ministry of Health of Nigeria (FMoH), the WHO Collaborating Centre at the Department of Psychiatry University of Ibadan, and the WHO in 2009 and 2010, the Nigerian Federal Ministry of Health volunteered to be one of the first countries that would implement the mhGAP programme and identified Osun State, one of the 36 states in the country, for the field trial. The contextualization process started in January 2011.

## Process and Observations

**Figure 1:**
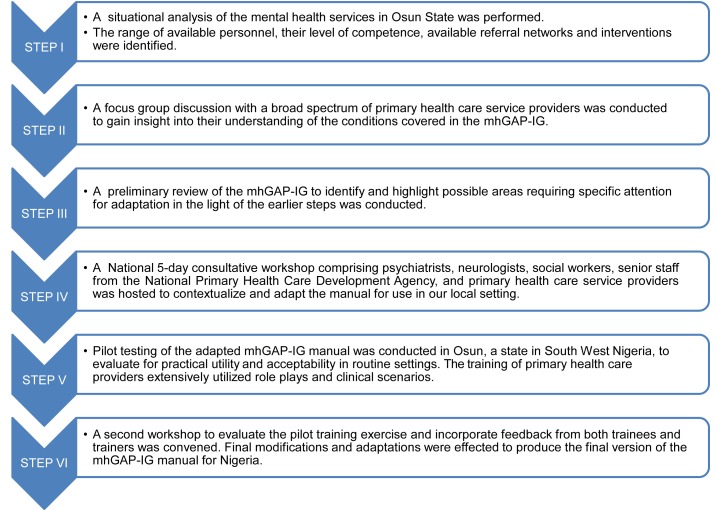
Flowchart describing the Mental Health Gap Action Programme – Intervention Guide contextualization process in Nigeria.

A series of step-wise activities were conducted to achieve the goal of contextualization (see Figure 1).

Step I: In order to provide a framework upon which to base the adaptation of the mhGAP-IG, a preliminary situational analysis of the mental health system was conducted. The tool used for the situational analysis was the World Health Organization Assessment Instrument for Mental Health Systems (WHO-AIMS), which was developed by the WHO Section on Mental Health and Substance Abuse, Geneva, for collecting essential information on the mental health system of a country or region. It comprises six domains: policy and legislative framework, mental health services, mental health in primary health care, human resources, public education and inter-sectoral links, and monitoring and research. It had been previously used to generate a national report on the mental health system in six selected states, out of the 36 states of Nigeria, excluding the current study site of Osun state [15].

The domains used were those of mental health services (domain 2), mental health in primary health care (domain 3), and human resources (domain 4) to collect information on mental health services, the organizational structure of the primary health care services, and the profile of the workforce in Osun state. The results revealed that there was no budgetary allocation for mental health, no designated desk officer for mental health in the state Ministry of Health, and mental health services were only available at the tertiary federal teaching hospitals located in the state. The primary health care workers knew very little about mental health conditions and there were no mental health services in the secondary facilities of the state. It also provided information about the level of training of the personnel, the available interventions (including which level of the clinical staff is administratively permitted to use which intervention), the range of mental disorders that they encounter, and the referral pathways within the health system.

**Figure 2:**
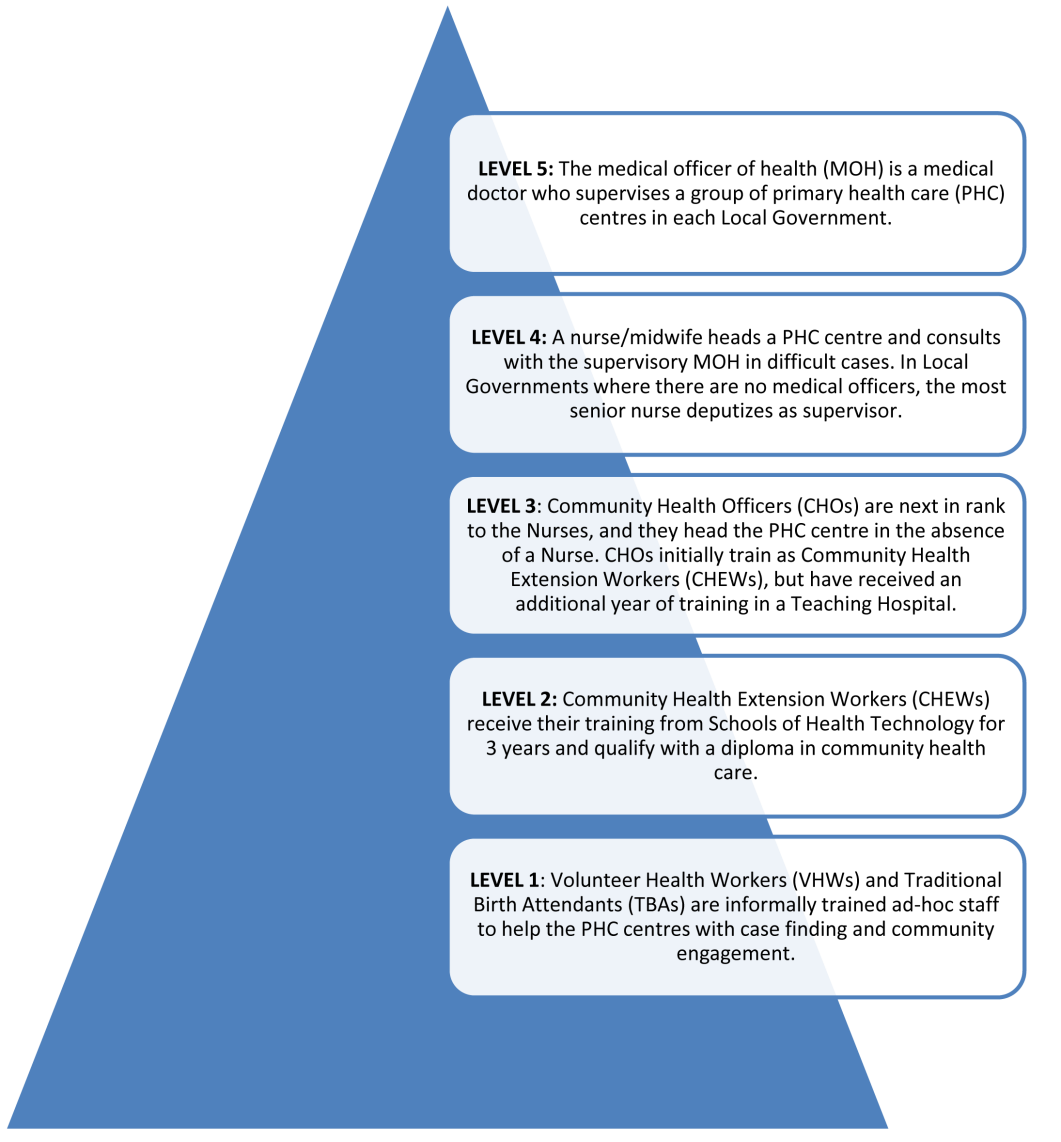
Organizational structure of the Nigerian primary health care system.

Step II: One focus group discussion was held with eight primary care providers, consisting of two nurses, three community health officers (CHOs), and three community health extension workers (CHEWs). The participants were selected to represent the major staffing categories of primary care providers in the country, and they were selected from different facilities within the state (see Figure 2). The aim of the discussion was to obtain from participants their views on: (1) the relevance of the major clinical entities covered by the mhGAP-IG to their day-to-day clinical practice; and (2) how easy or difficult was the format of the guide, especially in terms of layout and navigation in the course of routine practice. The discussion showed that the participants preferred to focus on fewer and more frequently encountered priority conditions. Dementia and behavioural disorders were rarely encountered conditions in their primary care settings, and were therefore excluded. Also, participants made some suggestions for the re-formatting of the layout of the guide to improve its user-friendliness in routine clinical work. The participants were not uncomfortable with the use of the mhGAP-IG, as their school training (CHEWs and CHOs) and daily clinical practice already involved the use of a manual, known as “Standing Orders,” a fairly comprehensive set of instructions for recognizing and making decisions, especially with respect to maternal and child health conditions but that barely mentions mental and neurological conditions. The Standing Order also allows the Nurses, CHOs, and CHEWs to prescribe essential medications including selected psychotropics such as amitriptylline, haloperidol, and carbamazepine, but they are not allowed to give intravenous injections [24].

Step III: A preliminary review of the mhGAP-IG was conducted by a small group of three persons (OG, LK, and WF). The aim of this preliminary review was to prepare the groundwork for the larger consultative workshop, by identifying and highlighting areas that may require expert deliberations. Such areas were duly noted and ear-marked for clarification and consensus decisions in the larger workshops, specifically with regard to whether changes were required, and what range of practical options are currently available in our context. The goal was to facilitate the activities of the workshop at which changes were to be considered and made, but the preliminary review did not limit the group discussions from considering the assigned modules in its entirety. The preliminary review was aided by the mhGAP Intervention Guide Contextualization Questionnaire, developed and provided by WHO to the Nigerian team to facilitate the local customization process. This tool is provided as a supporting information document.

Step IV: A national five-day consultative workshop was convened to conduct the adaptation and contextualization. Participants included psychiatrists with expertise in various subspecialties (general, addiction, mental health services, old-age, child and adolescent), neurologists (adult and pediatric), a social worker, a primary care nurse, and a general practitioner (GP). Also participating were senior technical staff of the National Primary Health Care Development Agency, the official body responsible for the standardization and development of the training programmes for primary care workers in Nigeria. The process received additional technical support from WHO Geneva and the country office. Workshop participants were experienced specialists, the majority of whom were already familiar with the mhGAP programme, and had prior public health research experience and qualifications.

The review process at the workshop was done in small groups, with each group focusing on a module of the mhGAP-IG, and the relevant sections of the Contextualization Questionnaire. Groups were composed with consideration for the areas of expertise of members. The groups were requested to identify areas in need of adaptation and to suggest options for the adaptation. Priority areas of focus included: (1) simplifying the language of the text as necessary to enhance comprehension for the profile of primary care workers; (2) ensuring that suggested interventions could be competently handled by the cadre of staff available; (3) identifying condition types and levels of severity for referrals, and specifying where such referrals are to be directed; and (4) ensuring that recommended interventions were either available within the health system or had alternative suggestions that could be utilized as substitutes. Some examples of these adaptations are provided in Table 1.

**Table 1:** Some examples of changes effected in two modules (Alcohol Use, Alcohol Use Disorders, Drug Use, and Drug Use Disorders) of the Mental Health Gap Action Programme – Intervention Guide in contextualizing it for Nigeria.

Page	Consideration	Response	Suggested Contextualization of mhGAP IG	Suggested Contextualization of Training Materials Used for Training on the mhGAP-IG	Rationale for Suggested Change
58	Emergency referral for methanol poisoning	Not an issue in this environment	Deleted	Not included in the training material	It is not relevant in this setting
58	Delirium as a complication	May not be recognized at PHC level	Retained in manual	Clarify and train to recognize delirium using simple language. For example, replace “fluctuating level of consciousness” with “knowing what is going on sometimes, and not knowing what is going on at other times.”	Consideration of the educational level of the PHC workers, and to facilitate easy comprehension.
59	Examine for nystagmus and ataxia of Wernicke encephalopathy. Opthalmoplegia may also occur	These terms may be difficult for PHC workers to assimilate	Changed to “examine for other neurological problems.”	Train to identify neurological complications and to refer when identified.	Consideration of the competence levels of PHC workers.
60	Standard drinks as a measure of alcohol consumption	Convert to local alcoholic drinks equivalent	Changed to “3½ bottles of beer or palmwine/burukutu”	Explain concept and demonstrate conversion to other locally available drinks.	Ensure practical familiarity with estimating alcohol consumption levels.
61	Reference to thiamine	Not locally available	Retained in manual but with addition of vitamin B complex, if unavailable	Explain rationale for use of thiamine and recommend use of vitamin B complex as a feasible alternative	Providing an available alternative
61	Liver damage	May be missed	Retained in manual	Cover in training to recognize stigmata of liver disease	Improve competence
61	Psychosocial interventions	Some options are not available	Revised to omit unavailable options such as “motivational enhancement therapy” and “contingency management therapy”	Illustrate possible interventions through role play	Emphasis on salient and practically useful options
61	Seek specialist support	Specialists are not readily available	Changed to “refer to a doctor or a specialist”	Encourage effective and prompt use of referrals when necessary	Strengthen referral mechanisms and caution against overzealousness
64	Treat other medical problems such as Wernicke or hepatic encephalopathy	Beyond competence of PHC workers	Changed to “If other medical problems are suspected, refer to a doctor for further assessment and treatment”	Elaborate on these medical problems to enhance easy recognition	Improve competence and prompt referrals

During this workshop, specific training needs of the different cadres of primary care workers were also identified and collated. These needs were grouped around modules reflecting the health conditions in the manual.

The recommendations from each group were subsequently discussed by the entire group of workshop participants. Each suggested change was debated, with the final adaptation reflecting the consensus of the larger group. A country-specific mhGAP-IG manual was produced at the end of the workshop.

Step V: Pilot testing. The adapted mhGAP manual provided the framework for a one-week training of primary care providers selected from several clinics in a local government area of Osun, a state in the south-western region of Nigeria. Trainees were 19 primary care workers (representing the cadres of nurses, CHOs, and CHEWs) and three supervisory GPs (see Figure 2). Trainers were three psychiatrists, one pediatric neurologist, and one social worker, all of whom had been involved in the contextualization consultative workshop. Training consisted of PowerPoint presentations, case discussions, small group work, and several role plays on each of the seven modules. Training materials were based on draft training materials developed and provided by the WHO but with extensive adaptations reflecting the products of the contextualization workshop. The effectiveness of the training was also evaluated using pre- and post-test assessment of trainees, with the aid of a questionnaire designed for this purpose, by the WHO. The language was simplified and adapted to suit our context. The participants (both the trainers and trainees) were requested to provide feedback on the experience of using the mhGAP for the training. In particular, information was requested about whether they thought the adapted guide was a feasible tool, specifically in regard to its applicability for routine use in primary care settings and to provide suggestions for further changes where there was a perceived need for such. This feedback was provided verbally during interactive sessions and via written comments after the post-training test. A WHO representative with international expertise in conducting these trainings was also present during this pilot training to provide technical guidance and assistance.

Step VI: A second national workshop was convened to consider the reports of the pilot training exercise and to make further changes to the manual. The workshop served to identify areas of additional difficulty that were not earlier envisaged during the first workshop. Modifications that proved to be superfluous and not very relevant were also identified and excluded. A final customized version was produced at the end of the second workshop.

## Lessons Learnt

We have described a process of contextualization and adaptation designed to make the mhGAP-IG relevant and appropriate to the extant health system in Nigeria. In the process, we used several methods to gather information, distil this obtained information, and ensure that the manual that was produced represents the consensus of important stakeholders in the field of mental health service delivery. We observed that the early efforts made to ensure that key stakeholders were actively engaged in the project were a very positive factor in the eventual production of a context-specific version of the manual. In particular, we note that a good understanding of the existing health system and the needs of the primary health care providers gained from the situational analysis and focus group discussion was crucial to focusing the subsequent activities appropriately.

The contextualization exercise revealed the major gaps in the existing training processes and curriculum for PHC workers and GPs with respect to identifying and treating mental and neurological disorders. This realization guided the structuring of the training materials and procedure in order to compensate for the deficiencies already anticipated. We relied on the extensive use of role plays, group work, and case scenarios to demonstrate clinical assessment, interviewing skills, and basic approaches to psycho-education. We noted that role plays were very helpful in promoting interactions within the group, building confidence in their competence to handle future clinical scenarios, and establishing their familiarity with the use of the charts. This method should be practically allotted more time than the didactic explanations and lectures about the conditions, and this is a point that should be reiterated. The existing familiarity with the use of manuals and charts, as detailed in their Standing Orders was utilized and parallels drawn with the mhGAP-IG to improve trainee confidence.

The exercise also illustrated the need for training sessions to be followed by a system of clinical support that emphasizes regular supervision from specialists and the provision of a structured referral network. We realized during the contextualization process, especially in the aspects that required modifications to the referral system (from “specialists” to “a medical officer of health”) that by including the GPs in the training programme, we were able to facilitate their supervisory roles and strengthen the existing referral systems. The training workshops ensured not just skill acquisition for the PHC workers and the GPs but also enhanced the development of a working relationship that cascaded from the PHC workers, through the GPs to the psychiatrists, working in a neighbouring tertiary facility. We strongly recommend this strengthening of a supervisory and supportive relationship among various cadres of health workers, in order to enhance the smooth implementation and continued on-the-job learning for the PHC staff. It also improved the confidence of the PHC staff, knowing that if they had difficulties, they could contact someone to ask for help and guidance.

## Limitations

This process was conceived and implemented as a national programme but could only be piloted in one state of Nigeria (Osun), because of funding constraints. It would have been more representative of the national spread to have conducted this process in at least one state from each of the six geopolitical zones. Nonetheless, it remains fair to say that unlike the distribution and organization of tertiary facilities and specialist services, the primary health care system is fairly uniform across the country. Furthermore, the technical support and panel of experts involved at the national workshops were drawn from across the country, and could therefore comment about the peculiarities of the PHC system in their respective regions.

Secondly, the organization of more focus group discussions, with each group comprising exclusively the same cadre of PHC staff may have generated a more representative sampling of opinions that would further enrich the initial situational analysis.

## Looking Forward

A resultant benefit of the inclusion of relevant stakeholders in the national workshops was the insight gained from the exercise by the participants from the National Primary Health Care Development Agency (NPHCDA) about the need for a revision of the curriculum for the training of primary care workers. There is already indication that this insight may lead to efforts to develop better streamlined training modules and programmes, using the mhGAP-IG in the relevant training institutions.

The contextualization effort was conducted within a larger programme of integration of mental health services into primary care in Nigeria. All the stakeholders of the health system were involved, including constant coordination with the health management system for the required authorization, engagement with policy makers to ensure availability of medications, human resource development to bring the training of providers to scale, integration within the health information system, monitoring and evaluation of the mental health service, and community sensitization. The impact of the programme in bridging the gap between need and available services will be evaluated at the appropriate time. Monitoring and evaluation mechanisms are also in place to review the implementation process of the mhGAP-IG over time. Only when this is done in a systematic manner will the overall effectiveness and impact of this process become clear. Negotiations and longer term planning efforts are also in place to ensure national financing and budgeting mechanisms are clearly drawn up, in order to guarantee its sustainability and successful nationwide scaling up. For now, there is only sufficient evidence to claim that the mhGAP-IG, adapted and contextualized to the extant health system, is a handy and useful manual for the accomplishment of the goal of mental health services scale-up in Nigeria.

## Conclusions

The mhGAP-IG is a new and potentially useful tool for scaling up mental health service in settings with scarcity of human resources, especially in LMICs where mental health specialists are few. The contextualization experience described here provides valuable insight and a pragmatic step-by-step guide for potential users of the mhGAP-IG. It details what needs to be done to optimize the use of this tool in a given context.

## Supporting Information

Questionnaire S1 **World Health Organization mhGAP – IG Contextualization Questionnaire.**
(PDF)

Presentation S1 **Final contextualized version of Nigeria's mhGAP-IG.**
(PDF)
