# Supplementary material for: Country Contextualization of the Mental Health Gap Action Programme Intervention Guide: A Case Study from Nigeria
Source: PLoS Med. 2013 Aug 20;10(8):e1001501. doi: 10.1371/journal.pmed.1001501 (PMC3747990; doi:10.1371/journal.pmed.1001501)
Supplement: Questionnaire S1 — World Health Organization mhGAP – IG Contextualization Questionnaire. (PDF) [file pmed.1001501.s001.pdf]

CONTEXTUALIZATION QUESTIONNAIRE

Page	Question to be considered	Response	Suggested contextualization of mhGAP Intervention Guide<br>1. Only make the most essential changes to the guide!<br>2. Only make changes to make it more suitable for the country	Suggested contextualization of training materials used for training on the mhGAP-IG<br>This question does not cover training issues that go beyond the learning of the contents and use of mhGAP-IG	Reasoning or technical basis for the suggested change<br><br>Further information needed before deciding on contextualization
**GENERAL PRINCIPLES OF CARE**					
P6 Left Top	Does the suggestion " Respond to the disclosure of private and distressing information (e.g. regarding sexual assault or self-harm) with sensitivity" require (country-specific) guidance ?			IF YES, add specific guidance in the training	
P7 Left middle	The text states "Pay special attention to national and international human rights standards (Box 1)" Does relevant national mental health legislation exist?		IF YES Add the national mental health legislation to Box 1 and change to ""Pay special attention to national legislation and international human rights standards (Box 1)"<br>IF NO. Change text to "Pay special attention to international human	IF YES Train not only on international but also on relevant national mental health legislation.	

Page	Question to be considered	Response	**Suggested contextualization of mhGAP Intervention Guide**<br>1. Only make the most essential changes to the guide!<br>2. Only make changes to make it more suitable for the country	**Suggested contextualization of training materials used for training on the mhGAP-IG**<br>This question does not cover training issues that go beyond the learning of the contents and use of mhGAP-IG	**Reasoning or technical basis for the suggested change**<br><br>**Further information needed before deciding on contextualization**
			rights standards (Box 1)"		
P7<br>Left<br>bottom	The test states " Ensure that the person understands the proposed treatment and provides free and informed consent to treatment." Are there any relevant national guidelines on informed consent (and limits of confidentiality) that staff should know?			IF YES, train on national guidelines for informed consent	
P7<br>Middle<br>Bottom	The text states "eg unprotected sex". Is this the best example?				

Page	Question to be considered	Response	Suggested contextualization of mhGAP Intervention Guide<br>1. Only make the most essential changes to the guide!<br>2. Only make changes to make it more suitable for the country	Suggested contextualization of training materials used for training on the mhGAP-IG<br>This question does not cover training issues that go beyond the learning of the contents and use of mhGAP-IG	Reasoning or technical basis for the suggested change<br><br>Further information needed before deciding on contextualization
**MASTER CHART**					
P 8	Answer for each of the modules: Is there good evidence that the common presentation is different in this specific country? What local idioms for signs and symptoms of mental, neurological and substances use disorders are used?				
	Depression			TRAINING TO TAKE INTO CONTEXT PECULIAR SOMATIC EXPRESSIONS	
	Psychosis				
	Epilepsy/Seizures				
	Developmental Disorders				
	Behavioural Disorders				
	Dementia				
	Alcohol Use Disorders				
	Drug Use Disorders				

**Page**	**Question to be considered**	**Response**	**Suggested contextualization of mhGAP Intervention Guide**<br>1. Only make the most essential changes to the guide!<br>2. Only make changes to make it more suitable for the country	**Suggested contextualization of training materials used for training on the mhGAP-IG**<br>This question does not cover training issues that go beyond the learning of the contents and use of mhGAP-IG	**Reasoning or technical basis for the suggested change**<br><br>**Further information needed before deciding on contextualization**
	Self-harm / suicide				

**Page**	**Question to be considered**	**Response**	**Suggested contextualization of mhGAP Intervention Guide**<br>1. Only make the most essential changes to the guide!<br>2. Only make changes to make it more suitable for the country	**Suggested contextualization of training materials used for training on the mhGAP-IG**<br>This question does not cover training issues that go beyond the learning of the contents and use of mhGAP-IG	**Reasoning or technical basis for the suggested change**<br><br>**Further information needed before deciding on contextualization**
**MODERATE-SEVERE DEPRESSION**					
P 10 Right top	Are "Interpersonal therapy, behavioural activation cognitive behavioural therapy " available/accessible now or within the next few years?	NO	KEEP TEXT		
P 10 Right top	Are "Structured physical activity programme, relaxation training or problem-solving treatment" available/accessible now or within the next few years?	YES PROBBLEM SOLVING AVAILABLE	KEEP TEXT	Consider, adding basic principles of problem-solving counselling to training materials	
P 10 Right top	The text states " DO NOT manage the complaint with injections or other ineffective treatments (e.g. vitamins)." Is "vitamins" the best example of ineffective care that is routinely provided the country?	NO	CHANGE TO SLEEPING TABLETS AND BLOOD TONIC		
P11	Are suicide, bipolar disorder, psychosis,	YES		IF NO, then	

Page	Question to be considered	Response	Suggested contextualization of mhGAP Intervention Guide<br>1. Only make the most essential changes to the guide!<br>2. Only make changes to make it more suitable for the country	Suggested contextualization of training materials used for training on the mhGAP-IG<br>This question does not cover training issues that go beyond the learning of the contents and use of mhGAP-IG	Reasoning or technical basis for the suggested change<br><br>Further information needed before deciding on contextualization
left column	alcohol and substance use among the selected modules in the country?			discuss this in training as one needs to CONSULT rather than MANAGE these conditions	
P11 left column bottom	The text states " Look for concurrent medical illness, especially signs / symptoms suggesting hypothyroidism, anaemia, tumours, stroke, hypertension, diabetes, HIV / AIDS, obesity or medication use, that can cause or exacerbate depression (such as steroids)". Given what is known about the epidemiology in the country, should the examples of diseases be somewhat changed?	YES	INCLUDE MALARIA AND DELETE STEROID AND OBESITY		
P 12 bottom	Are "Interpersonal therapy, behavioural activation cognitive behavioural therapy "	NO	KEEP TEXT		

Page	Question to be considered	Response	**Suggested contextualization of mhGAP Intervention Guide**<br>1. Only make the most essential changes to the guide!<br>2. Only make changes to make it more suitable for the country	**Suggested contextualization of training materials used for training on the mhGAP-IG**<br>This question does not cover training issues that go beyond the learning of the contents and use of mhGAP-IG	**Reasoning or technical basis for the suggested change**<br><br>**Further information needed before deciding on contextualization**
right	available/accessible now or within the next few years?				
P 12 bottom right	Are "Structured physical activity programme, relaxation training or problem-solving treatment" available/accessible now or within the next few years??	YES	KEEP TEXT		
P12 Right column Top	The text says "consult with a specialist". What doe s consult mean? (phone? refer?) What specialist should be consulted? (a psych nurse? A psychiatrist?)	NO	CHANGE TO 'REFER TO NEXT LEVEL OF CARE'		
P 12 bottom right	The text states "when psychosocial interventions prove ineffective, consider fluoxetine (but not oher SSRIs or TCAs)"<br>1) Is fluoxetine available in PHC?<br>2) Should children adolescents with depression be referred if first line of treatment (psychosocial) does not work or should they be given fluoxetine?	1.NO<br>2.YES	CHANGE TO 'REFER TO THE NEXT LEVEL OF CARE'		

Page	Question to be considered	Response	Suggested contextualization of mhGAP Intervention Guide<br>1. Only make the most essential changes to the guide!<br>2. Only make changes to make it more suitable for the country	Suggested contextualization of training materials used for training on the mhGAP-IG<br>This question does not cover training issues that go beyond the learning of the contents and use of mhGAP-IG	Reasoning or technical basis for the suggested change<br><br>Further information needed before deciding on contextualization
P 13 middle column, bottom	The text says: "Provide culture-relevant parent skills training if available". Is it available/accessible now or within the next few years?	NO		TO BE INCLUDED IN THE TRAINING	
P14 left column top	The text states: "Select an antidepressant from the National or WHO Formulary. Fluoxetine (but not other selective serotonin reuptake inhibitors (SSRIs)) and amitriptyline (as well as other tricyclic antidepressants (TCAs)) are antidepressants mentioned in the WHO Formulary and are on the WHO Model List of Essential Medicines" Which antidepressant drugs are available or will within the next few years be available /accessible in non-specialized care?	AMYTRIPTILINE	IF OTHER SSRI THAN FLUOXETINE IS WIDELY AVAILABLE/ACCESSIBLE and AFFORDABLE, then these may be added for use in adults but (not in adolescence, where fluoxetine remains the only med for adolescents.) If an SSRI (eg sertraline) is added, then adaptations should		

Page	Question to be considered	Response	Suggested contextualization of mhGAP Intervention Guide<br>1. Only make the most essential changes to the guide!<br>2. Only make changes to make it more suitable for the country	Suggested contextualization of training materials used for training on the mhGAP-IG<br>This question does not cover training issues that go beyond the learning of the contents and use of mhGAP-IG	Reasoning or technical basis for the suggested change<br><br>Further information needed before deciding on contextualization
			also be made to page 16, which has all the dosing info.		
P15 Left Colum n Top	The text mentions "CBT or IPT". Are these available now or within the next few years?	NO	DELETE FROM TEXT		

**Page**	**Question to be considered**	**Response**	**Suggested contextualization of mhGAP Intervention Guide**<br>1. Only make the most essential changes to the guide!<br>2. Only make changes to make it more suitable for the country	**Suggested contextualization of training materials used for training on the mhGAP-IG**<br>This question does not cover training issues that go beyond the learning of the contents and use of mhGAP-IG	**Reasoning or technical basis for the suggested change**<br><br>**Further information needed before deciding on contextualization**
**PSYCHOSIS**					
P 18 Middle	the texts says " Rule out psychotic symptoms due to:<br>» Alcohol or drug intoxication or withdrawal<br>(Refer to Alcohol use disorder / Drug use disorder module » ALC and » DRU Are these modules are among the selected conditions? Are these conditions common?	YES	IF THE CONDITION IS COMMON AND THE MODULE WAS NOT SELECTED, then add text on how to identify and manage alcohol intoxication and withdrawal.	IF NO, then discuss this in training as one needs to CONSULT rather than MANAGE these conditions	
P 18 Middle	The texts says " Rule out psychotic symptoms due to: " Delirium due to acute medical conditions<br>such as cerebral malaria, systemic infections /<br>sepsis, head injury" Is cerebral malaria an issue?	YES			
P 18	The text says "review and ensure	YES		Give guidance	

**Page**	**Question to be considered**	**Response**	**Suggested contextualization of mhGAP Intervention Guide**<br>1. Only make the most essential changes to the guide!<br>2. Only make changes to make it more suitable for the country	**Suggested contextualization of training materials used for training on the mhGAP-IG**<br>This question does not cover training issues that go beyond the learning of the contents and use of mhGAP-IG	**Reasoning or technical basis for the suggested change**<br><br>**Further information needed before deciding on contextualization**
Right column Middle	treatment adherence" Do health staff know how to do this?			and examples on adherence strategies during training?	
P19 Left column Top	The text says "several days of:<br>– Markedly elevated or irritable mood<br>– Excessive energy and activity<br>– Excessive talking<br>– Recklessness" These are features of mania. Are there any specific local lay terms for specifically mania?	NO	IF YES, consider adding these in brackets		
P19 Left column Bottom	The text states " Consider especially signs/symptoms suggesting stroke, diabetes, hypertension, HIV/AIDS, cerebral malaria or medications (e.g. steroids) ". Given what is known about the epidemiology in the country, should the examples of diseases be somewhat changed?	YES	ADD TYPHOID PSYCHOSIS		

Page	Question to be considered	Response	Suggested contextualization of mhGAP Intervention Guide<br>1. Only make the most essential changes to the guide!<br>2. Only make changes to make it more suitable for the country	Suggested contextualization of training materials used for training on the mhGAP-IG<br>This question does not cover training issues that go beyond the learning of the contents and use of mhGAP-IG	Reasoning or technical basis for the suggested change<br><br>Further information needed before deciding on contextualization
P20 Middle column Middle	the section title is "facilitate rehab in the community" Are there good local examples of how to do this work in the country?	YES	THE AMAUDO-ABIA PROJECT		
P20 Right column Top	The texts says "explore housing/assisted living support" What does this mean in the country?	NOT AVAILABLE	DELETE		NE FORMAL ASSISTED HOUSING
P20 Right bottom Right	Related to follow-up, what are locally feasible strategies for regular follow up (e.g. home visits, telephone calls, letter, contact through family and friends?).	YES	KEEP TEXT		
P21 Right side	Which antipsychotics are available in nonspecialized health care	CHLOPROM AZINE			
P22 Right bottom	the text says "Consult a specialist" What does consult mean? (phone? refer?) Who is a specialist? Does it include a psychiatric nurse?	KEEP CONSULT	CHANGE TO CONSULT A PSYCHIATRIST IS APPROPRIATE		

**Page**	**Question to be considered**	**Response**	**Suggested contextualization of mhGAP Intervention Guide**<br>1. Only make the most essential changes to the guide!<br>2. Only make changes to make it more suitable for the country	**Suggested contextualization of training materials used for training on the mhGAP-IG**<br>This question does not cover training issues that go beyond the learning of the contents and use of mhGAP-IG	**Reasoning or technical basis for the suggested change**<br><br>**Further information needed before deciding on contextualization**

**Page**	**Question to be considered**	**Response**	**Suggested contextualization of mhGAP Intervention Guide**<br>1. Only make the most essential changes to the guide!<br>2. Only make changes to make it more suitable for the country	**Suggested contextualization of training materials used for training on the mhGAP-IG**<br>This question does not cover training issues that go beyond the learning of the contents and use of mhGAP-IG	**Reasoning or technical basis for the suggested change**<br><br>**Further information needed before deciding on contextualization**
**BIPOLAR DISORDER**					
P 24 Left column	Are there any specific local terms for symptoms of mania?	NO	If yes, put the term between brackets after the scientific term.		
P 24 Left column Middle	The text states “whether hospitalization” was required. Is hospitalization available /accessible?	YES	If NO, then change the question whether it was very difficult to manage the person at home.		
P 24 Right Column top	The text states: "» Begin treatment of acute mania with lithium, valproate, carbamazepine or with antipsychotics. » BPD 3.1" Are these mood stabilizers available/accessible now or in the near future?	BEGIN TREATMENT WITH ANTIPSYCHOTICS (CHLOPROMAZINE) AND REFER TO A PHYSICIAN FOR			

Page	Question to be considered	Response	**Suggested contextualization of mhGAP Intervention Guide**<br>1. Only make the most essential changes to the guide!<br>2. Only make changes to make it more suitable for the country	**Suggested contextualization of training materials used for training on the mhGAP-IG**<br>This question does not cover training issues that go beyond the learning of the contents and use of mhGAP-IG	**Reasoning or technical basis for the suggested change**<br><br>**Further information needed before deciding on contextualization**
		FURTHER MANAGEMENT			
P 24 Right Column Middle	The text states "begin treatment with a mood-stabiliser" Are mood stabilizers available/accessible now or in the near future?	AS ABOVE			
P 25. Left column top	Are febrile illnesses (eg malaria, typhoid fever) common?	YES	IF Yes, add these in the list of concurrent illnesses. Change the text in the management box into "manage both the bipolar disorder and the concurrent condition, except for<br>- Malaria: provide appropriate treatment and follow-up for		

Page	Question to be considered	Response	**Suggested contextualization of mhGAP Intervention Guide**<br>1. Only make the most essential changes to the guide!<br>2. Only make changes to make it more suitable for the country	**Suggested contextualization of training materials used for training on the mhGAP-IG**<br>This question does not cover training issues that go beyond the learning of the contents and use of mhGAP-IG	**Reasoning or technical basis for the suggested change**<br><br>**Further information needed before deciding on contextualization**
			malaria<br>- Typhoid fever: provide appropriate treatment and follow-up for typhoid fever (i..e., one treats the cause and does not add mood stabilizers, although short course of antipsychotics is an option)”		
P 25. Left column top	Are there any other local prevalent illnesses that should be considered as concurrent condition? Are there any concurrent conditions listed that should be excluded as they are highly uncommon?	NO			
P 25 Right column	The text says " if the person is not on a mood stabilizer then begin one” Are mood stabilizers available/accessible	NO			

Page	Question to be considered	Response	**Suggested contextualization of mhGAP Intervention Guide**<br>1. Only make the most essential changes to the guide!<br>2. Only make changes to make it more suitable for the country	**Suggested contextualization of training materials used for training on the mhGAP-IG**<br>This question does not cover training issues that go beyond the learning of the contents and use of mhGAP-IG	**Reasoning or technical basis for the suggested change**<br><br>**Further information needed before deciding on contextualization**
Middle	now or in the near future?				
P 26 Left Column Top	The text covers treatment of bipolar disorder in pregnant/breastfeeding women.				
P 27 Left Column 2nd bullet	The text states " There needs to be some method for monitoring mood, such as keeping a daily mood log in which irritability, anger or euphoria are recorded." How would this be done?		KEEP A PERSONAL MOOD DAIRY AND A VISUAL SCALE FOR ILLITERATEA		
P 27 Right Column 2nd bullet	The text states " Consider supported employment for those who have difficulty obtaining or retaining normal employment." Is supported employment available?	NO			
P 28 Left Colum	The text states " Consider lithium, valproate, carbamazepine or antipsychotics for treatment of acute	WHEN APPROPRIATE THE			

Page	Question to be considered	Response	**Suggested contextualization of mhGAP Intervention Guide**<br>1. Only make the most essential changes to the guide!<br>2. Only make changes to make it more suitable for the country	**Suggested contextualization of training materials used for training on the mhGAP-IG**<br>This question does not cover training issues that go beyond the learning of the contents and use of mhGAP-IG	**Reasoning or technical basis for the suggested change**<br><br>**Further information needed before deciding on contextualization**
n<br>First bullet	mania." Are these mood stabilizers available/accessible now or in the near future?	PRIMARY CARE PHYSICIAN MAY USE THESE OPTIONS WHEN APPROPRIA TE			
P 29<br>Entire page	Are these mood stabilizers available?	YES	NOT AT THE PHC LEVEL		
P 29<br>last senten ce<br><br>and P30 3rd	The text says Consult a specialist. What is meant with a specialist here? What is meant with “consult”?	CONSULT A PSYCHIATRI ST			

**Page**	**Question to be considered**	**Response**	**Suggested contextualization of mhGAP Intervention Guide**<br>1. Only make the most essential changes to the guide!<br>2. Only make changes to make it more suitable for the country	**Suggested contextualization of training materials used for training on the mhGAP-IG**<br>This question does not cover training issues that go beyond the learning of the contents and use of mhGAP-IG	**Reasoning or technical basis for the suggested change**<br><br>**Further information needed before deciding on contextualization**
bullet					
P32 Right column<br><br>Second box	The text says: " Insert an intravenous (i.v.) line and give fluids slowly (30 drops / minute) . . . Give diazepam rectally (same dose as above) if i.v. line is difficult to establish"."<br>Is equipment for IV available in the facilities?				
P32 Right column Second box	The text says " Give diazepam i.v. 10 mg slowly (child: 1 mg / year of age) or lorazepam i.v. 4 mg (0.1 mg / kg), if available."<br>Which parenteral benzodiazepines is available in the country?				
P32 Middle column	The test says "suspect eclampsia" and the right column gives guidance on how to manage this..				

Page	Question to be considered	Response	**Suggested contextualization of mhGAP Intervention Guide**<br>1. Only make the most essential changes to the guide!<br>2. Only make changes to make it more suitable for the country	**Suggested contextualization of training materials used for training on the mhGAP-IG**<br>This question does not cover training issues that go beyond the learning of the contents and use of mhGAP-IG	**Reasoning or technical basis for the suggested change**<br><br>**Further information needed before deciding on contextualization**
and right column third box	Are there any maternal health workers? Are there any national guidelines for management of eclampsia? Are patients with eclampsia referred to and managed in hospital? (This would require adaptation according to national pregnancy and child birth guidelines)				
P32 Right column Fourth box	the text says **"**Neuroinfection**:** Manage the infection according to local guidelines."<br>Are patients with head injury and neuroinfections referred to and managed in hospital?<br>What are the local guidelines for these conditions?				
P32 Left column , fourth box	The text says "**Ask about ….**Other medical problems, medications or poisons (e.g. organophosphate poisoning), substance use (such as stimulant intoxication, benzodiazepine or				

Page	Question to be considered	Response	Suggested contextualization of mhGAP Intervention Guide<br>1. Only make the most essential changes to the guide!<br>2. Only make changes to make it more suitable for the country	Suggested contextualization of training materials used for training on the mhGAP-IG<br>This question does not cover training issues that go beyond the learning of the contents and use of mhGAP-IG	Reasoning or technical basis for the suggested change<br><br>Further information needed before deciding on contextualization
	alcohol withdrawal **» ALC** and **» DRU)**<br><br>Review the list according to the situation analysis of common causes of acute symptomatic seizures in the country				
P33 Right column First box	The text says "Phenobarbital 10 – 15 mg / kg, i.v. (rate of 100 mg / minute).<br>OR<br>**»** Phenytoin 15 – 18 mg / kg i.v. (through different line to diazepam) over 60 minutes. It is critical to have a very good i.v. line as the drug is very caustic and will cause significant local damage if it extravasates."<br><br>What parenteral formulations for AEDs are available? (Depending upon the availability, the protocol might need modification)				

**Page**	**Question to be considered**	**Response**	**Suggested contextualization of mhGAP Intervention Guide**<br>1. Only make the most essential changes to the guide!<br>2. Only make changes to make it more suitable for the country	**Suggested contextualization of training materials used for training on the mhGAP-IG**<br>This question does not cover training issues that go beyond the learning of the contents and use of mhGAP-IG	**Reasoning or technical basis for the suggested change**<br><br>**Further information needed before deciding on contextualization**
P34 left column, second box<br><br>(cf Master Chart)	The text says "convulsive movements". What is the local term for "convulsive movement or seizures"?<br><br>In countries that use IMCI, what local term has been used for "convulsion"?				
P34 right column, first box	The text says "consult a specialist"<br>What does "consult" mean here (phone? refer?)<br>What type of specialist?				
P34 Middle column third	The text says " Suspect an acute etiology<br>» Neuroinfection (meningitis / encephalitis)<br>» Cerebral malaria				

**Page**	**Question to be considered**	**Response**	**Suggested contextualization of mhGAP Intervention Guide**<br>1. Only make the most essential changes to the guide!<br>2. Only make changes to make it more suitable for the country	**Suggested contextualization of training materials used for training on the mhGAP-IG**<br>This question does not cover training issues that go beyond the learning of the contents and use of mhGAP-IG	**Reasoning or technical basis for the suggested change**<br><br>**Further information needed before deciding on contextualization**
box	» Head injury<br>» Hypoglycaemia or hyponatraemia<br>» Substance use / withdrawal"<br><br>Are all of these aetiologies relevant to the country?<br>What tests or facilities are available in the setting to identify these etiologies?				
P34 Right column Third box	The text says "suspect cerebral malaria in high endemic settings"<br><br>Is it a high endemic setting for malaria?				
P34 Left column Fourth box	the text says "fever"<br><br>In countries that use IMCI, what local term has been used for "fever"?<br>What are the local guidelines for management of fever?				
P34	Is IMCI available?		IF NO, adapt		

**Page**	**Question to be considered**	**Response**	**Suggested contextualization of mhGAP Intervention Guide**<br>1. Only make the most essential changes to the guide!<br>2. Only make changes to make it more suitable for the country	**Suggested contextualization of training materials used for training on the mhGAP-IG**<br>This question does not cover training issues that go beyond the learning of the contents and use of mhGAP-IG	**Reasoning or technical basis for the suggested change**<br><br>**Further information needed before deciding on contextualization**
Left column Fourth box					
P35 Right column second box	the text says "Initiate antiepileptic drug » EPI 2.1; either phenobarbital, phenytoin, carbamazepine or valproate. » EPI 2.3" Which of these AEDs are included in national list of essential medicines? Which of these AEDs are available in nonspecialized care (specify according to the facility)?		IF NOT AL LOF THESE AVAILABLE, delete as appropriate reference to these drugs here and in EPI 2.		
P36 Left column first box	Are developmental and behavioral disorders among the selected modules in the country?			IF NO, then discuss this in training as one needs to CONSULT rather than MANAGE these conditions	

**Page**	**Question to be considered**	**Response**	**Suggested contextualization of mhGAP Intervention Guide**<br>1. Only make the most essential changes to the guide!<br>2. Only make changes to make it more suitable for the country	**Suggested contextualization of training materials used for training on the mhGAP-IG**<br>This question does not cover training issues that go beyond the learning of the contents and use of mhGAP-IG	**Reasoning or technical basis for the suggested change**<br><br>**Further information needed before deciding on contextualization**
P36 Right Column third box	the text discusses giving vitamin K (1 mg m.i.) to new-borns.<br><br>Are there local guidelines on this? Is vitamin K available?				
P36 Left column third box	Are depression, psychosis, or self-harm among the selected modules in the country?			IF NO, then discuss this in training as one needs to CONSULT rather than MANAGE these conditions	
P37 Left column 6th bullet point	The text suggests patients to keep a seizure diary.<br>Does health personnel know how to teach this?			IF NO, teach this in the training	

Page	Question to be considered	Response	Suggested contextualization of mhGAP Intervention Guide<br>1. Only make the most essential changes to the guide!<br>2. Only make changes to make it more suitable for the country	Suggested contextualization of training materials used for training on the mhGAP-IG<br>This question does not cover training issues that go beyond the learning of the contents and use of mhGAP-IG	Reasoning or technical basis for the suggested change<br><br>Further information needed before deciding on contextualization
P 37 Left column 7th bullet point	The text says " For co-morbid medical conditions: Before prescribing antiepileptic drug, consider potential for drug-disease or drug-drug interaction. Consult the National or the WHO Formulary."<br>Which locally common co-morbid conditions and common concurrent drugs prescriptions should be born in mind?				
p37 right column , first bullet point	The text says "consult a specialist"<br><br>What does "consult" mean here (phone? refer?) What type of specialist?				
P39 Right column 6th	The text says " National laws related to the issue of driving and epilepsy need to be observed".				

Page	Question to be considered	Response	**Suggested contextualization of mhGAP Intervention Guide**<br>1. Only make the most essential changes to the guide!<br>2. Only make changes to make it more suitable for the country	**Suggested contextualization of training materials used for training on the mhGAP-IG**<br>This question does not cover training issues that go beyond the learning of the contents and use of mhGAP-IG	**Reasoning or technical basis for the suggested change**<br><br>**Further information needed before deciding on contextualization**
bullet point	What are the national laws on this? Are there any issues related to this?				

**Page**	**Question to be considered**	**Response**	**Suggested contextualization of mhGAP Intervention Guide**<br>1. Only make the most essential changes to the guide!<br>2. Only make changes to make it more suitable for the country	**Suggested contextualization of training materials used for training on the mhGAP-IG**<br>This question does not cover training issues that go beyond the learning of the contents and use of mhGAP-IG	**Reasoning or technical basis for the suggested change**<br><br>**Further information needed before deciding on contextualization**
**DEVELOPMENTAL DISORDERS**					
p 40 left column top	The text says 'Assess child's development using local developmental milestones…'.<br>Are local developmental milestones available?			If yes, include in training and provide developmental milestones as handout	
p 40 right column top	The text says: 'Manage nutrition problems, including iodine deficiency and medical conditions, using IMCI.<br>1) Has IMCI been adopted by the country and in use?<br>2) Is the person who manages developmental disorders also managing nutritional problems?		If answer to 1) is NO and 2) is YES, remove reference to IMCI and add reference to national guidelines for management of nutrional problems.<br>If answers to 1) is NO and 2) is NO, ask to refer to health worker responsible for managing nutrition problems.		

**Page**	**Question to be considered**	**Response**	**Suggested contextualization of mhGAP Intervention Guide**<br>1. Only make the most essential changes to the guide!<br>2. Only make changes to make it more suitable for the country	**Suggested contextualization of training materials used for training on the mhGAP-IG**<br>This question does not cover training issues that go beyond the learning of the contents and use of mhGAP-IG	**Reasoning or technical basis for the suggested change**<br><br>**Further information needed before deciding on contextualization**
p 40 Right column middle	The text says: 'Consider parent skills training, when available'<br>Is parent skills training for developmental problems available/accessible now or within the next few years?		.	If NO, consider adding parent skills training for developmental problems to staff within PHC system as part of mhGAP training.	
P40 Right column middle	The text says 'assess current level of adaptive functioning in consultation with specialist, if available'<br>Who is a specialist (psychiatrist, psych nurse, pediatrician?) Is a specialist available/accessible? Which modality is adopted for consultation (referral, phone)?		If YES, specify (professional category, modality)<br>'		
P 40 Right column	The text says 'facilitate and collaborate with community-based rehabilitation services'		IF NO, deliberate the pros and cons of keeping or removing		

Page	Question to be considered	Response	**Suggested contextualization of mhGAP Intervention Guide**<br>1. Only make the most essential changes to the guide!<br>2. Only make changes to make it more suitable for the country	**Suggested contextualization of training materials used for training on the mhGAP-IG**<br>This question does not cover training issues that go beyond the learning of the contents and use of mhGAP-IG	**Reasoning or technical basis for the suggested change**<br><br>**Further information needed before deciding on contextualization**
bottom	Are community-based rehabilitation services available/accessible now or within the next few years?		the relevant text.		
P 40<br>Right column bottom	The text says 'Refer to a specialist, if available, for further etiological assessment.<br>Who is a specialist (psychiatrist, psych nurse, pediatrician?) Is a specialist available/accessible?				
P 41<br>right column top	The text says 'Treat maternal depression. See Depression Module and other WHO documents for maternal depression and early childhood development'<br>Is the mhGAP Depression module adopted in the country?<br>Are other WHO relevant tools is use?		If OTHER RELEVANT WHO TOOLS for both identification and referral of maternal depression and early childhood development are in use, specify	If NO, consider including brief guidance on identification and referral of maternal depression and promotion of child development.	
P 41	The text says 'Treat according to relevant			IF NO, then	

Page	Question to be considered	Response	**Suggested contextualization of mhGAP Intervention Guide**<br>1. Only make the most essential changes to the guide!<br>2. Only make changes to make it more suitable for the country	**Suggested contextualization of training materials used for training on the mhGAP-IG**<br>This question does not cover training issues that go beyond the learning of the contents and use of mhGAP-IG	**Reasoning or technical basis for the suggested change**<br><br>**Further information needed before deciding on contextualization**
right column middle	modules: epilepsy, depression, behavioural disorders.' Are other mhGAP modules adopted?			discuss this in training as one needs to CONSULT rather than MANAGE these conditions	
P 41 right column bottom	The text says 'Consider additional family psychoeducation (and parent skills training for the specific problem behaviour)'<br>Is psychoeducation and parent skills training for behavioral problems available/accessible now or within the next few years? (see also Behavioural Disorders module)			If NO, consider adding family psychoeducation or parent skills training to staff within PHC system as part of mhGAP training	
P 41 right column bottom	The text says ' Consider cognitive behavioural therapy, if trained resources are available.'<br>Are CBT trained resources		IF NO, deliberate the pros and cons of keeping or removing the relevant text.		

Page	Question to be considered	Response	**Suggested contextualization of mhGAP Intervention Guide**<br>1. Only make the most essential changes to the guide!<br>2. Only make changes to make it more suitable for the country	**Suggested contextualization of training materials used for training on the mhGAP-IG**<br>This question does not cover training issues that go beyond the learning of the contents and use of mhGAP-IG	**Reasoning or technical basis for the suggested change**<br><br>**Further information needed before deciding on contextualization**
	available/accessible now or within the next few years?				

Page	Question to be considered	Response	Suggested contextualization of mhGAP Intervention Guide<br>1. Only make the most essential changes to the guide!<br>2. Only make changes to make it more suitable for the country	Suggested contextualization of training materials used for training on the mhGAP-IG<br>This question does not cover training issues that go beyond the learning of the contents and use of mhGAP-IG	Reasoning or technical basis for the suggested change<br><br>Further information needed before deciding on contextualization
**BEHAVIOURAL DISORDERS**					
P 44 right column up	The text says 'Consider parent skills training, when available'.<br>Is parent skills training available/accessible now or within the next few years?			If NO, consider adding parent skills training to staff within PHC system as part of mhGAP training	
P 44 right column up	The text says 'Consider psychosocial interventions such as cognitive behavioural therapy and social skills training based on availability'<br>Are such psychosocial interventions available/accessible now or within the next few years?		IF NO, deliberate the pros and cons of keeping or removing some or all of the relevant text.		
P 44 right column up	The text says 'When available use problem solving techniques' Are problem solving counseling techniques available/accessible now or within the next few years?			Consider, adding basic principles of problem-solving counselling to	

Page	Question to be considered	Response	**Suggested contextualization of mhGAP Intervention Guide**<br>1. Only make the most essential changes to the guide!<br>2. Only make changes to make it more suitable for the country	**Suggested contextualization of training materials used for training on the mhGAP-IG**<br>This question does not cover training issues that go beyond the learning of the contents and use of mhGAP-IG	**Reasoning or technical basis for the suggested change**<br><br>**Further information needed before deciding on contextualization**
				training materials	
P 44 right column middle	The text says 'If the carer has depression, use the relevant module of mhGAP-IG'<br>Is the mhGAP depression module adopted?			IF NO, then discuss this in training as one needs to CONSULT rather than MANAGE this condition	
P 44 right column middle	The text says 'Consult a specialist for methylphenidate'<br>Is methylphenidate available/accessible now or within the next few years??<br>Who is authorized to prescribe it?		IF NO, deliberate the pros and cons of keeping or removing some or all of the relevant text. .		
P 45 right column up	The text says 'Consider parent skills training when available.'<br>Is parent skills training for behavioural problems available/accessible now or within the next few year?		.	If NOT, consider adding parent skills training to staff within PHC system as part	

Page	Question to be considered	Response	**Suggested contextualization of mhGAP Intervention Guide**<br>1. Only make the most essential changes to the guide!<br>2. Only make changes to make it more suitable for the country	**Suggested contextualization of training materials used for training on the mhGAP-IG**<br>This question does not cover training issues that go beyond the learning of the contents and use of mhGAP-IG	**Reasoning or technical basis for the suggested change**<br><br>**Further information needed before deciding on contextualization**
				of mhGAP training	
P 45 right column up	The text says 'Consider psychosocial interventions such as CBT and social skills training based on availability.'<br>Are such psychosocial interventions available /accessible now or within the next few year?		If YES, refer to appropriate service.<br>If NO, deliberate the pros and cons of keeping or removing some or all of the relevant text.		
P 45 right column middle	The text says 'Consider methylphenidate only under conditions mentioned above for hyperkinetic disorder.'<br>Is methylphenidate available//accessible now or within the next few year?<br>Who is authorized to prescribe it (available at PHC level)?		If NOT, deliberate the pros and cons of removing it and removing relevant intervention details.<br>If YES, specify procedures.		
P46 right column up	The text says 'Provide parent education and home visits. In case of serious risk or non-response, involve other available resources and specialists'		.		

Page	Question to be considered	Response	Suggested contextualization of mhGAP Intervention Guide<br>1. Only make the most essential changes to the guide!<br>2. Only make changes to make it more suitable for the country	Suggested contextualization of training materials used for training on the mhGAP-IG<br>This question does not cover training issues that go beyond the learning of the contents and use of mhGAP-IG	Reasoning or technical basis for the suggested change<br><br>Further information needed before deciding on contextualization
	Which resources and specialists are available and shall be involved.<br>Are there national guidelines on this?				
P46 right column up	The text says 'Consider legal interventions according to local legislation' What legal interventions may be considered?		Specify as appropriate.		
P 48 column right up	The text says ' Reduce dose and consult a specialist' Which specialist (pediatrician? Psychiatrist? Psych nurse?) What is the modality for consultation (referral, phone?)				
P48 column right down	The text says 'If increase in height or weight stops, discontinue methylphenidate and consult a specialist' Which specialist (pediatrician? Psychiatrist? Psych nurse?) What is the modality for consultation (referral,				

Page	Question to be considered	Response	**Suggested contextualization of mhGAP Intervention Guide**<br>1. Only make the most essential changes to the guide!<br>2. Only make changes to make it more suitable for the country	**Suggested contextualization of training materials used for training on the mhGAP-IG**<br>This question does not cover training issues that go beyond the learning of the contents and use of mhGAP-IG	**Reasoning or technical basis for the suggested change**<br><br>**Further information needed before deciding on contextualization**
	phone?)				
P48 column right down	The text says 'If the child responds to methylphenidate, continue it for one year and then consult a specialist ' Which specialist (pediatrician? Psychiatrist? Psych nurse?) What is the modality for consultation (referral, phone?)				

Page	Question to be considered	Response	Suggested contextualization of mhGAP Intervention Guide<br>1. Only make the most essential changes to the guide!<br>2. Only make changes to make it more suitable for the country	Suggested contextualization of training materials used for training on the mhGAP-IG<br>This question does not cover training issues that go beyond the learning of the contents and use of mhGAP-IG	Reasoning or technical basis for the suggested change<br><br>Further information needed before deciding on contextualization
**DEMENTIA**					
P50 left column fourth bullet point	The text says: "Is there a clinical history of - Goitre, slow pulse, dry skin or hypothyroidism?<br>– Sexually transmitted infection (STI) or HIV?<br>– Cardiovascular disease?<br>– Poor dietary intake, malnutrition, anaemia?<br><br>1. Given what is known about the epidemiology in the country, should the examples of diseases be changed?<br>2. Are the non-specialist health care providers trained in detecting the above mentioned conditions?				
P50 right column	The text says "consult a specialist"<br>What does "consult" mean here (phone? refer?)				

Page	Question to be considered	Response	Suggested contextualization of mhGAP Intervention Guide<br>1. Only make the most essential changes to the guide!<br>2. Only make changes to make it more suitable for the country	Suggested contextualization of training materials used for training on the mhGAP-IG<br>This question does not cover training issues that go beyond the learning of the contents and use of mhGAP-IG	Reasoning or technical basis for the suggested change<br><br>Further information needed before deciding on contextualization
second box	What type of specialist?				
P50 middle column third box	The text says "**If there are any other unusual features, such as:**<br>**»** Onset before the age of 60 years<br>**»** Clinical hypothyroidism<br>**»** Cardiovascular disease<br>**»** History of previous STI or HIV<br>**»** History of head injury or stroke"<br><br>1. Given what is known about the epidemiology in the country, should the examples of diseases be changed?<br>2. Are the non-specialist health care providers trained in detecting the above mentioned conditions?				
P50 right column third	The text says "consult a specialist"<br>What does "consult" mean here (phone? refer?)<br>What type of specialist?				

Page	Question to be considered	Response	**Suggested contextualization of mhGAP Intervention Guide**<br>1. Only make the most essential changes to the guide!<br>2. Only make changes to make it more suitable for the country	**Suggested contextualization of training materials used for training on the mhGAP-IG**<br>This question does not cover training issues that go beyond the learning of the contents and use of mhGAP-IG	**Reasoning or technical basis for the suggested change**<br><br>**Further information needed before deciding on contextualization**
box					
P51 left column first box	Are "moderate-severe depression and psychosis" among the selected modules in the country?<br><br>Are there any other priority mental disorders that should be looked for?			IF NO, then discuss this in training as one needs to CONSULT rather than MANAGE these conditions	
P51 right column first box	Is development of cognitive impairment as a result of severe depression an important issue in the settings?				
P51 left column second box	Are there any local or lay terms for psychological and behavioural symptoms of dementia?				
P52	The text says: "Assess for				

**Page**	**Question to be considered**	**Response**	**Suggested contextualization of mhGAP Intervention Guide**<br>1. Only make the most essential changes to the guide!<br>2. Only make changes to make it more suitable for the country	**Suggested contextualization of training materials used for training on the mhGAP-IG**<br>This question does not cover training issues that go beyond the learning of the contents and use of mhGAP-IG	**Reasoning or technical basis for the suggested change**<br><br>**Further information needed before deciding on contextualization**
left column first box	» Hypertension (blood pressure)<br>» Hyperlipidaemia<br>» Diabetes<br>» Smoking<br>» Obesity (weight, waist-to-hip ratio)<br>» Heart disease (angina or myocardial infarction)<br>» Previous stroke or transient ischaemic attacks"<br><br>Are the non-specialist health care providers trained in detecting the above mentioned conditions?				
P52 right column first box	The text says: "Reduce cardiovascular risk factors according to local guidelines"<br><br>Are there local guidelines available for management of cardiovascular risk factors and diseases?				
P52	The text says "refer to appropriate				

**Page**	**Question to be considered**	**Response**	**Suggested contextualization of mhGAP Intervention Guide** 1. Only make the most essential changes to the guide! 2. Only make changes to make it more suitable for the country	**Suggested contextualization of training materials used for training on the mhGAP-IG** This question does not cover training issues that go beyond the learning of the contents and use of mhGAP-IG	**Reasoning or technical basis for the suggested change** **Further information needed before deciding on contextualization**
right column first box	specialists"<br><br>What type of specialist?				
P52 left column second box	The text says " Evaluate nutrition, eyesight, hearing, dentition, bladder and bowel function, and pain".<br><br>Are there any local guidelines available for evaluating and managing these physical conditions and/or are the non-specialists trained in doing these?				
P52 left column second box	The text says " Obtain urinalysis".<br><br>Are facilities for urinalysis for detecting urinary tract infection available in the settings?				
P52 left	The text says " Review medications, particularly those with significant				

Page	Question to be considered	Response	**Suggested contextualization of mhGAP Intervention Guide**<br>1. Only make the most essential changes to the guide!<br>2. Only make changes to make it more suitable for the country	**Suggested contextualization of training materials used for training on the mhGAP-IG**<br>This question does not cover training issues that go beyond the learning of the contents and use of mhGAP-IG	**Reasoning or technical basis for the suggested change**<br><br>**Further information needed before deciding on contextualization**
column second box	anticholinergic side-effects (such as amitriptyline (an antidepressant); many antihistamines; antipsychotic drugs)"<br><br>Which common concurrent medical prescriptions should be kept in mind?				
P52 right column second box	The text says "refer to appropriate specialists"<br><br>What type of specialist?				
P52 right column third box	The text says " Explore psychosocial interventions:<br>– financial support such as disability services<br>– information about the condition of the person<br>– respite care<br>– activation of community support network				

Page	Question to be considered	Response	**Suggested contextualization of mhGAP Intervention Guide**<br>1. Only make the most essential changes to the guide!<br>2. Only make changes to make it more suitable for the country	**Suggested contextualization of training materials used for training on the mhGAP-IG**<br>This question does not cover training issues that go beyond the learning of the contents and use of mhGAP-IG	**Reasoning or technical basis for the suggested change**<br><br>**Further information needed before deciding on contextualization**
	– family or individual therapy if available"<br><br>Are the psychosocial interventions mentioned above available/accessible now or within few years?				
P52 right column third box	Is "depression" among the selected modules in the country?			IF NO, then discuss this in training as one needs to CONSULT rather than MANAGE this condition	
P53 left column	Are there any locally validated tools for diagnosis of dementia?				
P 53 middle column	- what three common words can be used to assess memory?<br>- identify the questions to assess				

Page	Question to be considered	Response	**Suggested contextualization of mhGAP Intervention Guide**<br>1. Only make the most essential changes to the guide!<br>2. Only make changes to make it more suitable for the country	**Suggested contextualization of training materials used for training on the mhGAP-IG**<br>This question does not cover training issues that go beyond the learning of the contents and use of mhGAP-IG	**Reasoning or technical basis for the suggested change**<br><br>**Further information needed before deciding on contextualization**
	orientation<br>- identify the questions to assess language skills				
P53 right column	Identify the questions to ask the key informant for each of the bullet points				
P54 left column	Who should be informed of the results of the assessment?				
P54 left column	In your opinion what information is required to be given to the person and family?				
P54 middle column	Who would be delivering the psychosocial interventions for cognitive symptoms and functioning?<br><br>How would these be delivered?				
P54 middle and	Who would be providing advice on promoting independence, functioning and mobility and how this would be				

Page	Question to be considered	Response	**Suggested contextualization of mhGAP Intervention Guide**<br>1. Only make the most essential changes to the guide!<br>2. Only make changes to make it more suitable for the country	**Suggested contextualization of training materials used for training on the mhGAP-IG**<br>This question does not cover training issues that go beyond the learning of the contents and use of mhGAP-IG	**Reasoning or technical basis for the suggested change**<br><br>**Further information needed before deciding on contextualization**
right column	provided?<br><br>What social resources are available for liaison?				
P55 left column	"Managing behavioural and psychological symptoms"<br><br>Is there any intervention here which requires to be added/revised?				
P55 left column	Are there any local policies/guidelines in relation to abuse of old people?				
P55 middle column	"interventions for carers"<br><br>- What social services/resources are available? How can the liaison be built?<br>- who would deliver the psychosocial interventions to carers and how would these be delivered?<br>- are advanced psychosocial				

Page	Question to be considered	Response	Suggested contextualization of mhGAP Intervention Guide<br>1. Only make the most essential changes to the guide!<br>2. Only make changes to make it more suitable for the country	Suggested contextualization of training materials used for training on the mhGAP-IG<br>This question does not cover training issues that go beyond the learning of the contents and use of mhGAP-IG	Reasoning or technical basis for the suggested change<br><br>Further information needed before deciding on contextualization
	interventions such as problem-solving counseling, cognitive-behaviour interventions available/accessible now or in the next few years?				
P55 right column	Are there any changes required in the follow-up plan?				
P56 left cloumn	The text says "consult a specialist"<br><br>What does "consult" mean here (phone? refer?)<br>What type of specialist?				
P56 left column	If haloperidol is not available, what other antipsychotic medications are available and can be used?				
P56 left column	When and how the antipsychotic medications can be discontinued?				
P56 left	Is "psychosis" among the selected modules in the country?			IF NO, then discuss this in	

**Page**	**Question to be considered**	**Response**	**Suggested contextualization of mhGAP Intervention Guide**<br>1. Only make the most essential changes to the guide!<br>2. Only make changes to make it more suitable for the country	**Suggested contextualization of training materials used for training on the mhGAP-IG**<br>This question does not cover training issues that go beyond the learning of the contents and use of mhGAP-IG	**Reasoning or technical basis for the suggested change**<br><br>**Further information needed before deciding on contextualization**
column				training as one needs to CONSULT rather than MANAGE these conditions	
P56 middle column	Are acetylcholinesterase inhibitors or memantine available in the country?<br><br>Are adequate specialists available for supervision for above treatment?<br><br>Is it possible to make a specific diagnosis of Alzheimer's Disease?				

Page	Question to be considered	Response	Suggested contextualization of mhGAP Intervention Guide<br>1. Only make the most essential changes to the guide!<br>2. Only make changes to make it more suitable for the country	Suggested contextualization of training materials used for training on the mhGAP-IG<br>This question does not cover training issues that go beyond the learning of the contents and use of mhGAP-IG	Reasoning or technical basis for the suggested change<br><br>Further information needed before deciding on contextualization
**ALCOHOL USE AND ALCOHOL USE DISORDERS**					
p.58 Right Column top	The texts states " If methanol poisoning is suspected, refer to hospital for emergency management." Is methanol poisoning an issue in this setting?		IF NO remove from chart		
p.58 Right Column Middle	The text states " Treat in hospital or detoxification centre if available." Are detox centers available?		If NOT available, replace with in hospital.	If hospital beds are also not available then family supervision at home can be a replacement	
p.58 Right Column Middle	The text states "if withdrawal is complicated by delirium". Do staff know how to recognize delirium?			Delirium needs to be trained	
P59 Middle	the text states " Examine for nystagmus and ataxia of Wernicke’s encephalopathy.			Cover in training	

Page	Question to be considered	Response	**Suggested contextualization of mhGAP Intervention Guide** 1. Only make the most essential changes to the guide! 2. Only make changes to make it more suitable for the country	**Suggested contextualization of training materials used for training on the mhGAP-IG** This question does not cover training issues that go beyond the learning of the contents and use of mhGAP-IG	**Reasoning or technical basis for the suggested change** **Further information needed before deciding on contextualization**
Column Top	Ophthalmoplegia may occur in severe cases." Do staff know how to do this examination? Is the text understandable?				
P59 Middle Column Bottom	The text states " *Exclude other common causes or confusion, such as infections, hypoxia, hypoglycaemia, hepatic encephalopathy, and cerebrovascular accidents." To what extent do the staff have the means and ability to exclude these causes?*				
P 59 Right Column Top	The text refers to thiamine. Is it available or will it be available in the few years?		IF NO, deliberate the pros and cons of keeping or removing some or all of the relevant text.		
P 60 Left Column Middle	The text states: "5 or more standard drinks (or 60 g alcohol)*. . . ***** *A standard drink is a measure of the amount of pure alcohol consumed, usually between 8 g and 12 g. If the amount of alcohol*		Convert to local standard drinks		

Page	Question to be considered	Response	Suggested contextualization of mhGAP Intervention Guide<br>1. Only make the most essential changes to the guide!<br>2. Only make changes to make it more suitable for the country	Suggested contextualization of training materials used for training on the mhGAP-IG<br>This question does not cover training issues that go beyond the learning of the contents and use of mhGAP-IG	Reasoning or technical basis for the suggested change<br><br>Further information needed before deciding on contextualization
	contained in a standard drink in that country is outside these limits, the number of standard drinks may need to be adjusted." Is there implicit use of the concept of standard drink in the country? If yes, how much alcohol is in it roughly?				
P 60 Left Column Middle	The text states " *This may be done informally or by the use of a questionnaire such as the WHO-AUDIT, or the WHO–ASSIST."* Are these 2 WHO tools in use in the country? Will they potentially be used in the next few years?				
P 61 Left column Last bullet	The text states "harm to the liver" Do staff know how to recognize "harm to the liver"?			Cover in training - yellow skin and eyes, drowsy or confused	
P 61 Right Colum	The text refers again to thiamine. Is it available or will it be available in the next few years?		IF NO, see above		

Page	Question to be considered	Response	**Suggested contextualization of mhGAP Intervention Guide**<br>1. Only make the most essential changes to the guide!<br>2. Only make changes to make it more suitable for the country	**Suggested contextualization of training materials used for training on the mhGAP-IG**<br>This question does not cover training issues that go beyond the learning of the contents and use of mhGAP-IG	**Reasoning or technical basis for the suggested change**<br><br>**Further information needed before deciding on contextualization**
n Top					
P 61 Right Column Middle	The text states: "Consider referral to a self-help group (such as Alcoholics Anonymous), or a residential therapeutic community" Are self help groups, AA and residential therapeutic communities all available or potentially will be available in the next few years?		IF NO, deliberate the pros and cons of keeping or removing some or all of the relevant text in the chart Even if one removea therapeutic communities from chart it is recommended to leave self-help groups in because it provides an impetus for people to create them - they can be easily developed without funding,		
P 61 Right Colum	The text states "If available, provide psychosocial interventions such as family counselling or therapy, problem-solving		IF NO, deliberate the pros and cons of keeping or removing		

Page	Question to be considered	Response	**Suggested contextualization of mhGAP Intervention Guide** 1. Only make the most essential changes to the guide! 2. Only make changes to make it more suitable for the country	**Suggested contextualization of training materials used for training on the mhGAP-IG** This question does not cover training issues that go beyond the learning of the contents and use of mhGAP-IG	**Reasoning or technical basis for the suggested change** **Further information needed before deciding on contextualization**
n Bottom	counselling or therapy, cognitive behavioural therapy, motivational enhancement therapy, or contingency management therapy. » INT". Are these available/accessible now or within the next few years?		some or all of the relevant text in the chart.		
P 61 Right Column Bottom	The text states "Consider referral to a specialized treatment facility" Are these available/accessible now or within the next few years?				
P 61 Right Column Bottom	The text states "Follow up as needed, frequently initially" Who does the follow-up mean? What does frequently mean?		Depends on resources but at least monthly visit either to clinic or at home by someone from the team		
P 61 Right Column	The text states "Seek specialist support as needed." Who are specialists (psych nurses?)? How would their support be sought? (referral, phone call?)				

Page	Question to be considered	Response	**Suggested contextualization of mhGAP Intervention Guide** 1. Only make the most essential changes to the guide! 2. Only make changes to make it more suitable for the country	**Suggested contextualization of training materials used for training on the mhGAP-IG** This question does not cover training issues that go beyond the learning of the contents and use of mhGAP-IG	**Reasoning or technical basis for the suggested change** **Further information needed before deciding on contextualization**
Bottom					
P62 Left column Middle	Will it be difficult for the staff to ask about each of the following problems? "– accidents, driving while intoxicated<br>– relationship problems<br>– medical problems such as liver disease / stomach ulcers<br>– legal / financial problems<br>– sex while intoxicated and that is later regretted or risky<br>– alcohol-related violence including domestic violence"				
P 62 Left column 4th bullet	Is the concept of "taking a chronological history" clear?				
P 62 Middle column	Do staff have the skills and means to identify "evidence of long-term heavy alcohol consumption, such as liver				

Page	Question to be considered	Response	Suggested contextualization of mhGAP Intervention Guide<br>1. Only make the most essential changes to the guide!<br>2. Only make changes to make it more suitable for the country	Suggested contextualization of training materials used for training on the mhGAP-IG<br>This question does not cover training issues that go beyond the learning of the contents and use of mhGAP-IG	Reasoning or technical basis for the suggested change<br><br>Further information needed before deciding on contextualization
2nd bullet	disease (swollen liver, peripheral signs of liver injury), cerebellar or peripheral nerve damage."?				
P 62 Middle column 2nd bullet	Do staff have the skills and means to investigate: "liver enzymes and full blood examination."?		Delete reference to these investigations if these cannot be done in the clinic		
P 62 section 2.3	The section covers self-help group. Are these available/accessible within the next few years?		leave self-help groups in because it provides an impetus for people to create them - they can be easily developed without funding,		
P 62 section 2.4 1st bullet	Are there local agencies providing this sort of support?				
P. 63	The text states" Where available, work		Adapt text advising the		

**Page**	**Question to be considered**	**Response**	**Suggested contextualization of mhGAP Intervention Guide** 1. Only make the most essential changes to the guide! 2. Only make changes to make it more suitable for the country	**Suggested contextualization of training materials used for training on the mhGAP-IG** This question does not cover training issues that go beyond the learning of the contents and use of mhGAP-IG	**Reasoning or technical basis for the suggested change** **Further information needed before deciding on contextualization**
1st bullet	with local agencies and community resources to find supported housing or assisted living facilities, as well as independent living facilities, if these are needed. Carefully consider the capacity of the person and the availability of alcohol or other substances in advising and facilitating optimal housing arrangements." Are any of these housing arrangements available/accessible now or within the next few years?		clinician to acknowledge that people living conditions have an impact on their health and treatment. It might be possible to mediate a return to living with a friend or relative.		
P 63 First column 2nd last bullet	The text states "Inform them [i.e. family members] about and help them access support groups (e.g. self-help groups for families and carers)" Are such groups available/accessible now or within the next few years?		leave self-help groups in because it provides an impetus for people to create them - they can be easily developed without funding,		
P63 Left column	The text states " Clarify the confidential nature of the health care discussion, including in what circumstances parents				

Page	Question to be considered	Response	**Suggested contextualization of mhGAP Intervention Guide**<br>1. Only make the most essential changes to the guide!<br>2. Only make changes to make it more suitable for the country	**Suggested contextualization of training materials used for training on the mhGAP-IG**<br>This question does not cover training issues that go beyond the learning of the contents and use of mhGAP-IG	**Reasoning or technical basis for the suggested change**<br><br>**Further information needed before deciding on contextualization**
last bullet	or other adults will be given information" Does the country have legislation on limits of informed consent? What are the expectations on the health worker in terms of consent?				
P63 Middle column last bullet	The last bullet gives parenting advice. Does the advice make sense with this cultural context?				
P63 Third column 2nd last bullet	the text mentions TWO [standard] drinks. Does the country use (implicitly) use the concept of standard drinks?				
P63 Third column last bullet	the text states " should be offered what social support services are available, including additional post natal visits, parenting training, and child care during medical visits" To what extent are each of				

**Page**	**Question to be considered**	**Response**	**Suggested contextualization of mhGAP Intervention Guide**<br>1. Only make the most essential changes to the guide!<br>2. Only make changes to make it more suitable for the country	**Suggested contextualization of training materials used for training on the mhGAP-IG**<br>This question does not cover training issues that go beyond the learning of the contents and use of mhGAP-IG	**Reasoning or technical basis for the suggested change**<br><br>**Further information needed before deciding on contextualization**
	these services available?				
P64 Left column 2nd bullet	the text states "people with impaired hepatic metabolism (e.g. liver failure, elderly)" can staff identify impaired hepatic metabolism?		Replace “In people with impaired hepatic metabolism (eg liver failure, elderly)” with “When hepatic metabolism is suspected, use single low dose initially (5-10 mg) and determine the duration of action of this dose before prescribing further doses. Refer to hospital or make telephone consultation with a liver specialist of alcohol disease specialist”		
P64 Left	The text refers again to thiamine. Is it available or will it be available in the next				

Page	Question to be considered	Response	Suggested contextualization of mhGAP Intervention Guide<br>1. Only make the most essential changes to the guide!<br>2. Only make changes to make it more suitable for the country	Suggested contextualization of training materials used for training on the mhGAP-IG<br>This question does not cover training issues that go beyond the learning of the contents and use of mhGAP-IG	Reasoning or technical basis for the suggested change<br><br>Further information needed before deciding on contextualization
column 3rd bullet	few years?				
P64 Left column last bullet	The text states " Ensure adequate fluid intake and electrolyte requirements are met. Correct potassium and magnesium levels that are typically low." Do staff have the means and skills to do all of this?		If NO, delete from chart. It could be replaced with something on ensuring access to food for a few days…		
P64 Middle column 2nd bullet.	What does "consult a specialist mean" in terms of who and how.				
P64 Middle column 3rd bullet.	The text states: " Consider and treat other medical problems (e.g. Wernicke’s encephalopathy, hepatic encephalopathy, gastrointestinal bleeding, head injury with or without subdural haematoma)." Do the staff have the skills and means to do this?				

Page	Question to be considered	Response	**Suggested contextualization of mhGAP Intervention Guide**<br>1. Only make the most essential changes to the guide!<br>2. Only make changes to make it more suitable for the country	**Suggested contextualization of training materials used for training on the mhGAP-IG**<br>This question does not cover training issues that go beyond the learning of the contents and use of mhGAP-IG	**Reasoning or technical basis for the suggested change**<br><br>**Further information needed before deciding on contextualization**
P 64 Right Column Bottom	The text implicitly expresses the option to withdrawal outside inpatient setting. Is there any national policy or legislation on this option?				
P64 Middle column 3rd bullet	The text refers again to thiamine. Is it available or will it be available in the next few years?				
P64 Last bullet	Do the staff have the skills and means to" Always consider other causes of delirium and hallucinations (e.g. head injury, hypoglycaemia, infection (most commonly pneumonia), hypoxia, hepatic encephalopathy or cerebrovascular accidents)."				
P65	Are " acamprosate, naltrexone and disulfiram" available in the country? In specialized care? In non.specialized				

**Page**	**Question to be considered**	**Response**	**Suggested contextualization of mhGAP Intervention Guide**<br>1. Only make the most essential changes to the guide!<br>2. Only make changes to make it more suitable for the country	**Suggested contextualization of training materials used for training on the mhGAP-IG**<br>This question does not cover training issues that go beyond the learning of the contents and use of mhGAP-IG	**Reasoning or technical basis for the suggested change**<br><br>**Further information needed before deciding on contextualization**
	care?				

Page	Question to be considered	Response	**Suggested contextualization of mhGAP Intervention Guide**<br>1. Only make the most essential changes to the guide!<br>2. Only make changes to make it more suitable for the country	**Suggested contextualization of training materials used for training on the mhGAP-IG**<br>This question does not cover training issues that go beyond the learning of the contents and use of mhGAP-IG	**Reasoning or technical basis for the suggested change**<br><br>**Further information needed before deciding on contextualization**
**DRUG USE AND DRUG USE DISORDERS**					
P66	Are these the main emergency presentations in the country?		If NO, remove those emergency presentations which are rare, and focus on the more common emergency problems. Use the names of specific locally used drugs if possible.		
	Is NALOXONE available?		If NOT, replace with support breathing (either mouth to mouth (if considered acceptable), bag and mask or ventilator)		
	Are the drugs used to treat opioid withdrawal available?		If NOT, focus on those drugs which are available, clonidine, weak opioids and other		

**Page**	**Question to be considered**	**Response**	**Suggested contextualization of mhGAP Intervention Guide**<br>1. Only make the most essential changes to the guide!<br>2. Only make changes to make it more suitable for the country	**Suggested contextualization of training materials used for training on the mhGAP-IG**<br>This question does not cover training issues that go beyond the learning of the contents and use of mhGAP-IG	**Reasoning or technical basis for the suggested change**<br><br>**Further information needed before deciding on contextualization**
			symptomatic medication as listed in the section on opioid withdrawal.		
Pg 68	Are detoxification services available?		If there are not detoxification facilities, detoxification can usually be done at home with family or other person's supports and frequent visits from the health centre or to the heatlh centre.		
	Are there mechanisms for the supervised dispensing of benzodiazepines? DRU3.2		If not, then reconsider prescribing benzodiazepines, using detoxification instead as the first option, switching to gradual reduction only if severe withdrawal symptoms develop		

Page	Question to be considered	Response	**Suggested contextualization of mhGAP Intervention Guide**<br>1. Only make the most essential changes to the guide!<br>2. Only make changes to make it more suitable for the country	**Suggested contextualization of training materials used for training on the mhGAP-IG**<br>This question does not cover training issues that go beyond the learning of the contents and use of mhGAP-IG	**Reasoning or technical basis for the suggested change**<br><br>**Further information needed before deciding on contextualization**
Pg 69	Are urine drugs screens available		Consider purchasing cheap urine dipstick tests. They do not require expensive laboratory services. If these are still not available consider still collecting the sample and putting it in the fridge for later reference if needed but relying on clinical assessment skills. Samples should only be sent away to external laboratories if the results are likely to make a difference to management when they return.		
	Are other tests available		Consider referring for		

Page	Question to be considered	Response	**Suggested contextualization of mhGAP Intervention Guide**<br>1. Only make the most essential changes to the guide!<br>2. Only make changes to make it more suitable for the country	**Suggested contextualization of training materials used for training on the mhGAP-IG**<br>This question does not cover training issues that go beyond the learning of the contents and use of mhGAP-IG	**Reasoning or technical basis for the suggested change**<br><br>**Further information needed before deciding on contextualization**
			expensive tests as needed. If they are completely unavailable, remove.		
Pg 70	Are self help groups such as NA available		If YES, but not NA, then replace with the names of the more common groups. If NOT, then consider starting self help groups in your area by providing a meeting room once a week where people can meet, perhaps facilitated by a staff member if there are no volunteers initially.		
	Are there services in the community to provide housing and employment?		If NOT, see what resources are in the patients extended family and social network, and		

Page	Question to be considered	Response	**Suggested contextualization of mhGAP Intervention Guide**<br>1. Only make the most essential changes to the guide!<br>2. Only make changes to make it more suitable for the country	**Suggested contextualization of training materials used for training on the mhGAP-IG**<br>This question does not cover training issues that go beyond the learning of the contents and use of mhGAP-IG	**Reasoning or technical basis for the suggested change**<br><br>**Further information needed before deciding on contextualization**
			whether the health care staff can try and repair facilitate the repair of any of these relationships, thus enabling the patient to find housing or employment.		
71	Does the advice on breastfeeding seem too complicated.		Simplify - breastfeed.		
P72	Are none of the medications in the first colums available?		Use symptomatic medications listed in the last paragraph, analgaesics (including weak opioids, paracetamol, NSAIDs) anti-emetics, sedatives(in low doses)		
	Are there no detoxification facilities?		Detoxification can be done on an outpatient		

Page	Question to be considered	Response	Suggested contextualization of mhGAP Intervention Guide<br>1. Only make the most essential changes to the guide!<br>2. Only make changes to make it more suitable for the country	Suggested contextualization of training materials used for training on the mhGAP-IG<br>This question does not cover training issues that go beyond the learning of the contents and use of mhGAP-IG	Reasoning or technical basis for the suggested change<br><br>Further information needed before deciding on contextualization
			basis or at home under the supervision of family or friends. Frequent visits to or by the clinic can help.		
	No agonist maintenance treatment?		Use detoxification and relapse prevention (and advice on how to avoid overdose) for the management of opioid withdrawal. Consider if it is possible to start opioid agonist service with methadone or buprenorphine if none exists.		
	Does the most common drug not receive enough prominence?		If cannabis, for example, is the most common drug, consider rearranging the text to		

Page	Question to be considered	Response	Suggested contextualization of mhGAP Intervention Guide<br>1. Only make the most essential changes to the guide!<br>2. Only make changes to make it more suitable for the country	Suggested contextualization of training materials used for training on the mhGAP-IG<br>This question does not cover training issues that go beyond the learning of the contents and use of mhGAP-IG	Reasoning or technical basis for the suggested change<br><br>Further information needed before deciding on contextualization
			gie more prominence to cannabis withdrawal.		

Page	Question to be considered	Response	Suggested contextualization of mhGAP Intervention Guide<br>1. Only make the most essential changes to the guide!<br>2. Only make changes to make it more suitable for the country	Suggested contextualization of training materials used for training on the mhGAP-IG<br>This question does not cover training issues that go beyond the learning of the contents and use of mhGAP-IG	Reasoning or technical basis for the suggested change<br><br>Further information needed before deciding on contextualization
**SELF-HARM/SUICIDE**					
P74 Right column Middle	the text states "» If Acute Pesticide Intoxication, follow Pesticide Intoxication Management. » SUI 2.3", Does self-poisoning with pesticides occur in this country?		IF NO, remove this sentence and drop section SUI2.3		
P74 right column, bottom (also 2x on P75 right column and on P77 left column	The text says "consult mental health specialist if available" Is there a mental health specialist available/accessible?				

**Page**	**Question to be considered**	**Response**	**Suggested contextualization of mhGAP Intervention Guide**<br>1. Only make the most essential changes to the guide!<br>2. Only make changes to make it more suitable for the country	**Suggested contextualization of training materials used for training on the mhGAP-IG**<br>This question does not cover training issues that go beyond the learning of the contents and use of mhGAP-IG	**Reasoning or technical basis for the suggested change**<br><br>**Further information needed before deciding on contextualization**
)					
P76 Left column top	The text states " Depression, Alcohol or drug use disorders, Bipolar disorder, Psychosis, Epilepsy, Behavioural disorders" Are these disorders among the selected modules in the country?			IF NO, then discuss this in training as one needs to CONSULT rather than MANAGE these conditions	
P77 Right column, second 2nd bullet	The text says "restrict access to the means of self-harm (e.g. pesticides and other toxic substances, medication, firearms)." Are pesticides and firearms an issue as a means of suicide in the country? Are there any (other) preventable, common means of suicide in the country?				
P77 Right column	The text says "if available, such as crisis centers and local mental health centre." Are crisis centers and local mental health				

Page	Question to be considered	Response	**Suggested contextualization of mhGAP Intervention Guide**<br>1. Only make the most essential changes to the guide!<br>2. Only make changes to make it more suitable for the country	**Suggested contextualization of training materials used for training on the mhGAP-IG**<br>This question does not cover training issues that go beyond the learning of the contents and use of mhGAP-IG	**Reasoning or technical basis for the suggested change**<br><br>**Further information needed before deciding on contextualization**
3rd bullet	centers available?				
P77 Middle column last bullet	The text states "Consider problem-solving therapy for treating people with acts of self-harm in the last year, if sufficient human resources are available. » INT" Is problem-solving therapy available/accessible now or within the next few years?				
P78 Middle column 1st bullet	The text states "Maintain regular contact (via telephone, home visits, letters, or contact cards)" How is contact maintained in the country?				
P78 Right column 1st bullet	What are the means of self-harm? And what are the feasible ways to reduce access to these means?				
P78	The text states "Develop policies to				

Page	Question to be considered	Response	Suggested contextualization of mhGAP Intervention Guide<br>1. Only make the most essential changes to the guide!<br>2. Only make changes to make it more suitable for the country	Suggested contextualization of training materials used for training on the mhGAP-IG<br>This question does not cover training issues that go beyond the learning of the contents and use of mhGAP-IG	Reasoning or technical basis for the suggested change<br><br>Further information needed before deciding on contextualization
Right column 1st bullet	reduce harmful use of alcohol as a component of suicide prevention particularly within populations with high prevalence of alcohol use" Is alcohol prevalent? Do relevant policies exist?				

Page	Question to be considered	Response	Suggested contextualization of mhGAP Intervention Guide<br>1. Only make the most essential changes to the guide!<br>2. Only make changes to make it more suitable for the country	Suggested contextualization of training materials used for training on the mhGAP-IG<br>This question does not cover training issues that go beyond the learning of the contents and use of mhGAP-IG	Reasoning or technical basis for the suggested change<br><br>Further information needed before deciding on contextualization
**OTHER SIGNIFICANT EMOTIONAL OR MEDICALLY UNEXPLAINED COMPLAINTS**					
P80 Right column Bottom	The text states "Where available, consider one of the following treatments: structured physical activity programme, DEP 2.4 behavioural activation, relaxation training, or problem-solving treatment. INT" Is this available/accessible now or within the next few years?	YES	IF NO, deliberate the pros and cons of keeping or removing some or all of the relevant text in the chart.		
