# Supplementary material for: Country Contextualization of the Mental Health Gap Action Programme Intervention Guide: A Case Study from Nigeria
Source: PLoS Med. 2013 Aug 20;10(8):e1001501. doi: 10.1371/journal.pmed.1001501 (PMC3747990; doi:10.1371/journal.pmed.1001501)
Supplement: Presentation S1 — Final contextualized version of Nigeria's mhGAP-IG. (PDF) [file pmed.1001501.s002.pdf]

mhGAP Intervention Guide for Nigeria

for mental, neurological and substance use disorders
in non-specialized health settings

Version 1.0

World Health
Organization

mental health Gap Action Programme

Table of contents

Foreword...i

Acknowledgements...............................ii

Abbreviations and Symbols..................iii

I. Introduction....................................1

II. General Principles of Care.............6

III. Master Chart..................................6

IV Modules

1. Moderate-Severe Depression.......................10
2. Psychosis..18
3. Bipolar Disorder...24
4. Epilepsy / Seizures......................................32
5. Developmental Disorders............................40
6. Behavioural Disorders.................................44
7. Dementia..44
8. Alcohol Use and Alcohol Use Disorder...........50
9. Drug Use and Drug Use Disorders.................58
10. Self-harm / Suicide......................................66
11. Other Significant Emotional or Medically Unexplained Complaints................80

V Advanced Psychosocial Interventions..................82

Foreword

From the Office of the Director of Public Health Federal Ministry of Health Abuja Nigeria.

Acknowledgements

Adapted mhGAP Invervention Guide for Nigeria

Local Experts

O. Gureje, Department of Psychiatry, College of Medicine, University of Ibadan; Dr S. Jibrin, Federal Ministry of Health; Dr. J. Abdulmalik, University College Hospital Ibadan; Dr. A. Ojagbemi, University College Hospital, Ibadan; Dr. J. Eaton cbm, Nigeria; Dr. K. Adebayo LAUTECH Osogbo; F. Jinadu, Yaba Neuro Psychiatric Hospital, Lagos; A. Adesope, Community Health Services Dept., NPHCDA, Abuja; S. Oluwole, Department of Medicine, College of Medicine, University of Ibadan; N. Ihebuzor, Community Health Services Dept., NPHCDA; S. Abimbola, Community Health Services Dept., NPHCDA; M. Salisu, Department of Paediatrics LASUTH, Lagos; J. Abdumalik, Department of Psychiatry, College of Medicine, University of Ibadan; B. Buhari, Prof. R. Uwakwe, Nnamdi Asikwe Teaching Hospital, Nnewi, Anambra; M. Oche, Federal Medical Centre Makurdi; S. Sale, Department of Psychiatry, Aminu Kano Teaching Hospital, Kano; A. Atanda, Medical Social Services Department, University of Ibadan; T. Alabi, PHC Co-Ordinator, Olorunda LG, Osogbo; S. Okunoye, PHC Olorunda LG, Osogbo; W. Fadahunsi, Department of Psychiatry, College of Medicine, University of Ibadan.

WHO Offices
T. Yasamy, WHO Geneva; E. Musa, WHO, Nigeria; L. Kola, WHO, Nigeria.

Administrative Support
B. Salako, Department of Psychiatry, College of Medicine, University of Ibadan.

For more information, please contact:

World Health Organization
Country Office,
Plot 1620A, Maitama Sule Street,
Off Yakubu Gowon Crescent,
Asokoro, Abuja Nigeria.

Printed by: **Gbala Printing Works,**
Mokola, Ibadan, Oyo State, Nigeria.
Tel: 08055203857

Abbreviations and Symbols

Abbreviations

AIDS	acquired immune deficiency syndrome
CBT	congnitive behavioural therapy
HIV	human immunodeficiency virus
i.m.	intramuscular
IMCI	Integrated Management
IPT	interpersonal psychotherapy
i.v.	intravenous
mhGAP	mental health Gap Action Programme
mhGAP-IG	mental health Gap Action Programme Intervention Guide
OST	opioid-susbstitution therapy
SSPI	selective serotonin reuptake inhibitor
STI	sexually transmitted infection
TCA	tricyclic antidepressant

Symbols

- Babies / small children
- Children / adolescents
- Women
- Pregnant women
- Adult
- Older person
- Attention/Problem
- Go to / look at / Skip out of this module
- Do not
- Further information
- Refer to hospital
- Medication
- Psychosocial intervention
- Consult specialist
- Terminate assessment

YES if YES

NO if NO

Introduction

Mental Health Gap Action Programme (mhGAP) – background

About four out of five people in low- and middle-income countries who need services for mental, neurological and substance use conditions do not receive them. Even when available, the interventions often are neither evidence-based nor of high quality. WHO recently launched the Mental Health Gap Action Programme (mhGAP) for low- and middle-income countries with the objective of scaling up care for mental, neurological and substance use disorders. This mhGAP Intervention Guide (mhGAP-IG) has been developed to facilitate mhGAP-related delivery of evidence-based interventions in non-specialized health-care settings.

There is a widely shared but mistaken idea that all mental health interventions are sophisticated and can only be delivered by highly specialized staff. Research in recent years has demonstrated the feasibility of delivery of pharmacological and psychosocial interventions in non-specialized health-care settings. The present model guide is based on a review of all the science available in this area and presents the interventions recommended for use in low- and middle-income countries. The mhGAP-IG includes guidance on evidence-based interventions to identify and manage a number of priority conditions. The priority conditions included are depression, psychosis, bipolar disorders, epilepsy, developmental and behavioural disorders in children and adolescents, dementia, alcohol use disorders, drug use disorders, self-harm / suicide and other significant emotional or medically unexplained complaints. These priority conditions were selected because they represent a large burden in terms of mortality, morbidity or disability, have high economic costs, and are associated with violations of human rights.

Development of the mhGAP Intervention Guide (mhGAP-IG)

The mhGAP-IG has been developed through an intensive process of evidence review. Systematic reviews were conducted to develop evidence-based recommendations. The process involved a WHO Guideline Development Group of international experts, who collaborated closely with the WHO Secretariat. The recommendations were then converted into clearly presented stepwise interventions, again with the collaboration of an international group of experts. The mhGAP-IG was then circulated among a wider range of reviewers across the world to include all the diverse contributions.

The mhGAP-IG is based on the mhGAP Guidelines on interventions for mental, neurological and substance use disorders (http://www.who.int/mental_health/mhgap/evidence/en/). The mhGAP Guidelines and the mhGAP-IG will be reviewed and updated in 5 years. Any revision and update before that will be made to the online version of the document.

Purpose of the mhGAP Intervention Guide

The mhGAP-IG has been developed for use in non-specialized health-care settings. It is aimed at health-care providers working at first- and second-level facilities. These health-care providers may be working in a health centre or as part of the clinical team at a district-level hospital or clinic. They include general physicians, family physicians, nurses and clinical officers. Other non-specialist health-care providers can use the mhGAP-IG with necessary adaptation. The first-level facilities include the health-care centres that serve as first point of contact with a health professional and provide outpatient medical and nursing care. Services are provided by general practitioners or physicians, dentists, clinical officers, community nurses, pharmacists and midwives, among others. Second-level facilities include the hospital at the first referral level responsible for a district or a defined geographical area containing a defined population and governed by a politico-administrative organization, such as a district health management team. The district clinician or mental health specialist supports the first- level health-care team for mentoring and referral.

The mhGAP-IG is brief so as to facilitate interventions by busy non-specialists in low- and middle-income countries. It describes in detail what to do but does not go into descriptions of how to do. It is important that the non-specialist health-care providers are trained and then supervised and supported in using the mhGAP-IG in assessing and managing people with mental, neurological and substance use disorders.

Introduction

It is not the intention of the mhGAP-IG to cover service development. WHO has existing documents that guide service development. These include a tool to assess mental health systems, a Mental Health Policy and Services Guidance Package, and specific material on integration of mental health into primary care. Information on mhGAP implementation is provided in Mental Health Gap Action Programme: Scaling up care for mental, neurological and substance use disorders. Useful WHO documents and their website links are given at the end of the introduction.

Although the mhGAP-IG is to be implemented primarily by non-specialists, specialists may also find it useful in their work. In addition, specialists have an essential and substantial role in training, support and supervision. The mhGAP-IG indicates where access to specialists is required for consultation or referral. Creative solutions need to be found when specialists are not available in the district. For example, if resources are scarce, additional mental health training for non-specialist health-care providers may be organized, so that they can perform some of these functions in the absence of specialists. Specialists would also benefi t from training on public health aspects of the programme and service organization. Implementation of the mhGAP-IG ideally requires coordinated action by public health experts and managers, and dedicated specialists with a public health orientation.

Adaptation of the mhGAP-IG

The mhGAP-IG is a model guide and it is essential that it is adapted to national and local situations. Users may select a subset of the priority conditions or interventions to adapt and implement, depending on the contextual differences in prevalence and availability of resources. Adaptation is necessary to ensure that the conditions that contribute most to burden in a specific country are covered and that the mhGAP-IG is appropriate for the local conditions that affect the care of people with mental, neurological and substance use disorders in the health facility. The adaptation process should be used as an opportunity to develop a consensus on technical issues across disease conditions; this requires involvement of key national stakeholders. Adaptation will include language translation and ensuring that the interventions are acceptable in the sociocultural context and suitable for the local health system.

MhGAP implementation – key issues

Implementation at the country level should start from organizing a national stakeholder's meeting, needs assessment and identification of barriers to scaling-up. This should lead to preparing an action plan for scaling up, advocacy, human resources development and task shifting of human resources, financing and budgeting issues, information system development for the priority conditions, and monitoring and evaluation.

District-level implementation will be much easier after national-level decisions have been put into operation. A series of coordination meetings is initially required at the district level. All district health officers need to be briefed, especially if mental health is a new area to be integrated into their responsibilities. Presenting the mhGAP-IG could make them feel more comfortable when they learn that it is simple, applicable to their context, and could be integrated within the health system. Capacity building for mental health care requires initial training and continued support and supervision. However, training for delivery of the mhGAP-IG should be coordinated in such a way as not to interrupt ongoing service delivery.

Introduction

How to use the mhGAP-IG

» The mhGAP-IG starts with **“General Principles of Care”**. It provides good clinical practices for the interactions of healthcare providers with people seeking mental health care. All users of the mhGAP-IG should familiarize themselves with these principles and should follow them as far as possible.

» The mhGAP-IG includes a **“Master Chart”,** which provides information on common presentations of the priority conditions. This should guide the clinician to the relevant modules.

– In the event of potential **co-morbidity (two disorders present at the same time),** it is important for the clinician to confirm the co-morbidity and then make an overall management plan for treatment.

– The most serious conditions should be managed first. Follow-up at next visit should include checking whether symptoms or signs indicating the presence of any other priority condition have also improved. If the condition is flagged as an emergency, it needs to be managed first. For example, if the person is convulsing, the acute episode should be managed first before taking detailed history about the presence of epilepsy.

» The modules, organized by individual priority conditions, are a tool for clinical decision-making and management. Each module is in a different colour to allow easy differentiation. There is an introduction at the beginning of each module that explains which condition(s) the module covers.

» Each of the modules consists of two sections. The first section is the assessment and management section. In this section, the contents are presented in a framework of flowcharts with multiple decision points. Each decision point is identified by a number and is in the form of a question. Each decision point has information organized in the form of three columns –
“assess, decide and manage”.

Assess **Decide** **Manage**

– The left-hand column includes the details for assessment of the person. It is the assess column, which guides users how to assess the clinical condition of a person.
Users need to consider all elements of this column before moving to the next column.

– The middle column specifies the different scenarios the health-care provider might be facing. This is the decide column.

– The right-hand column describes suggestions on how to manage the problem. It is the manage column. It provides information and advice, related to particular decision points, on psychosocial and pharmacological interventions. The management advice is linked (crossreferenced) to relevant intervention details that are too detailed to be included in the flowcharts. The relevant intervention details are identified with codes. For example, DEP 3 means the intervention detail number three for the Moderate-Severe Depression Module.

– The mhGAP-IG uses a series of symbols to highlight certain aspects within the assess, decide and manage columns of the flowcharts. A list of the symbols and their explanation is given in the section Abbreviations and Symbols.

NOTE: Users of the mhGAP-IG need to start at the top of the assessment and management section and move through all the decision points to develop a comprehensive management plan for the person.

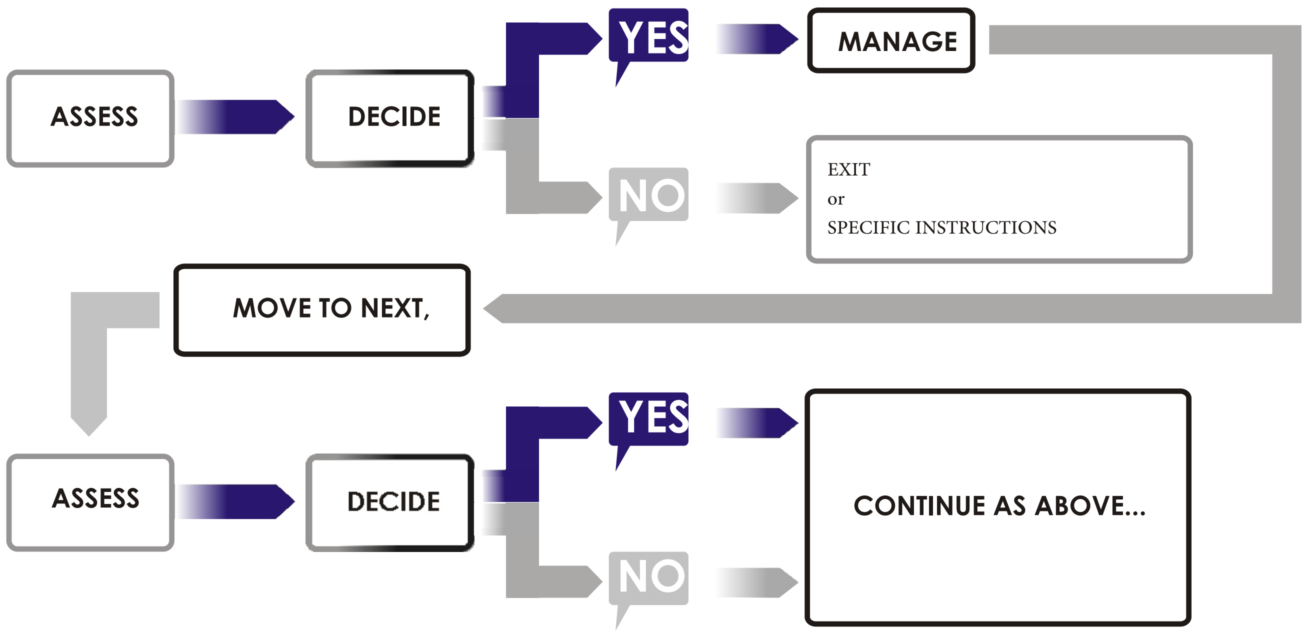

Instructions to use flow charts correctly and comprehensively

» The second section of each module consists of ***intervention details*** which provides more information on follow-up, referral, relapse prevention, and more technical details of psychosocial/non-pharmacological and pharmacological treatments, and important side-effects or interactions. The intervention details are presented in a generic format. They will require adaptation to local conditions and language, and possibly addition of examples and illustrations to enhance understanding, acceptability and attractiveness.

» Although the mhGAP-IG is primarily focusing on clinical interventions and treatment, there are opportunities for the health-care providers to provide evidence-based interventions to prevent mental, neurological and substance use disorders in the community. Prevention boxes for these interventions can be found at the end of some of the conditions.

» Section V covers **"Advanced Psychosocial Interventions"** For the purposes of the mhGAP-IG, the term "advanced psychosocial interventions" refers to interventions that take more than a few hours of a health-care provider's time to learn and typically more than a few hours to implement.
Such interventions can be implemented in non-specialized care settings but only when sufficient human resource time is made available. Within the fl owcharts in the modules, such interventions are marked by the abbreviation INT indicating that these require a relatively more intensive use of human resources.

Introduction

Related WHO documents that can be downloaded from the following links:

Assessment of iodine deficiency disorders and monitoring their elimination: A guide for programme managers. Third edition (updated 1st September 2008)
http://www.who.int/nutrition/publications/micronutrients/iodine_deficiency/9789241595827/en/index.html

CBR: A strategy for rehabilitation, equalization of opportunities, poverty reduction and social inclusion of people with disabilities (Joint Position Paper 2004)
Http://whqlibdoc.who.int/publications/2004/9241592389_eng.pdf

Clinical management of acute pesticide intoxication: Prevention of suicidal behaviours
http://www.who.int/mental_health/prevention/suicide/Pesticides_intoxication.pdf

Epilepsy: A manual for medical and clinical officers in Africa
Http://www.who.int/mental_health/media/en/639.pdf

IASC guidelines on mental health and psychosocial support in emergency settings
http://www.who.int/mental_health/emergencies/guidelines_Iasc_mental_health_psychosocial_april_2008.pdf

IMCI care for development: For the healthy growth and development of children
http://www.who.int/child_adolescent_health/documents/Imci_care_for_development/en/index.html

Improving health systems and services for mental health
http://www.who.int/mental_health/policy/services/mhsystems/En/index.html

Infant and young child feeding – tools and materials
http://www.who.int/child_adolescent_health/documents/Iycf_brochure/en/index.html

Integrated management of adolescent and adult illness / Integrated management of childhood illness (IMAI/IMCI)
Http://www.who.int/hiv/topics/capacity/en/

Integrated management of childhood illness (IMCI)
http://www.who.int/child_adolescent_health/topics/Prevention_care/child/imci/en/index.html

Integrating mental health into primary care – a global perspective
http://www.who.int/mental_health/policy/Integratingmhintoprimarycare2008_lastversion.pdf

Lancet series on global mental health 2007
Http://www.who.int/mental_health/en/

Mental Health Gap Action Programme (mhGAP)
Http://www.who.int/mental_health/mhGAP/en/

mhGAP Evidence Resource Centre
http://www.who.int /mental_health/mhgap/evidence/en/

Pharmacological treatment of mental disorders in primary health care
http://www.who.int/mental_health/management/psychotropic/En/index.html

Pregnancy, childbirth, postpartum and newborn care: A guide for essential practice
http://www.who.int/making_pregnancy_safer/documents/924159084x/en/index.html

Preventing suicide: a resource series
http://www.who.int/mental_health/resources/preventingsuicide/En/index.html

Prevention of cardiovascular disease: guidelines for assessment and management of cardiovascular risk
http://www.who.int/cardiovascular_diseases/guidelines/Prevention_of_Cardiovascular_Disease/en/index.html

Prevention of mental disorders: Effective interventions and policy options
http://www.who.int/mental_health/evidence/en/prevention_of_Mental_disorders_sr.pdf

Promoting mental health: Concepts, emerging evidence, practice
http://www.who.int/mental_health/evidence/MH_Promotion_Book.pdf

World Health Organization Assessment Instrument for Mental Health Systems (WHO-AIMS)
Http://www.who.int/mental_health/evidence/WHO-AIMS/en/

GPC

General Principles of Care

GPC

Health -care providers should follow good clinical practices in their interactions with all people seeking care. They should respect the privacy of people seeking care for mental, neurological and substance use disorders, foster good relationship with them and their carers, and respond to those seeking care in a non-judgemental, non-stingmatizing and supportive manner. The following key actions should be considered when implementing the mhGAP Intervention Guide. These are not repeated in each module.

1. Communication with people seeking care and their carers

» Ensure that communication is clear, empathic, and sensitive to age, gender, culture and language differences.

» Be friendly, respectful and non-judgmental at all times.

» Use simple and clear language.

» Respond to the disclosure of private and distressing information (e.g. regarding sexual assault or self-harm) with sensitivity.

» Provide information to the person on their health status in terms that they can understand.

» Ask the person for their own understanding of the condition.

2. Assessment

» Take a medical history, history of the presenting complaint(s), past history and family history, as relevant.

» Perform a general physical assessment.

» Assess, manage or refer, as appropriate, for any concurrent medical conditions.

» Assess for psychosocial problems, noting the past and ongoing social and relationship issues, living and financial circumstances, and any other ongoing stressful life events.

3. Treatment and monitoring

» Determine the importance of the treatment to the person as well as their readiness to participate in their care.

» Determine the goals for treatment for the affected person and create a management plan that respects their preferences for care (also those of their carer, if appropriate).

» Devise a plan for treatment continuation and follow-up, in consultation with the person.

» Inform the person of the expected duration of treatment, potential side-effects of the intervention, any alternative treatment options, the importance of adherence to the treatment plan, and of the likely prognosis.

» Address the person's questions and concerns about treatment, and communicate realistic hope for better functioning and recovery.

» Continually monitor for treatment effects and outcomes, drug interactions (including with alcohol, over-the-counter medication and complementary/traditional medicines), and adverse effects from treatment, and adjust accordingly.

» Facilitate referral to specialists, where available and as required.

» Make efforts to link the person to community support.

» At follow-up, reassess the person's expectations of treatment, clinical status, understanding of treatment and adherence to the treatment and correct any misconceptions.

» Encourage self-monitoring of symptoms and explain when to seek care immediately.

» Document key aspects of interactions with the person and the family in the case notes.

» Use family and community resources to contact people who have not returned for regular follow-up.

» Request more frequent follow-up visits for pregnant women or women who are planning a pregnancy.

» Assess potential risks of medications on the fetus or baby when providing care to a pregnant or breastfeeding woman.

» Make sure that the babies of women on medications who are breastfeeding are monitored for adverse effects or withdrawal and have comprehensive examinations if required.

» Request more frequent follow-up visits for older people with priority conditions, and associated autonomy loss or in situation of social isolation.

» Ensure that people are treated in a holistic manner, meeting the mental health needs of people with physical disorders, as well as the physical health needs of people with mental disorders.

4. Mobilizing and providing social support

» Be sensitive to social challenges that the person may face, and note how these may influence the physical and mental health and well-being.

General Principles of Care

- Where appropriate, involve the carer or family member in the person's care.
- Encourage involvement in self-help and family support groups, where available.
- Identify and mobilize possible sources of social and community support in the local area, including educational, housing and vocational supports.
- For children and adolescents, coordinate with schools to mobilize educational and social support, where possible.
- Pay special attention to confidentiality, as well as the right of the person to privacy.
- With the consent of the person, keep carers informed about the person's health status, including issues related to assessment, treatment, follow-up, and any potential side effects.
- Prevent stigma, marginalization and discrimination, and promote the social inclusion of people with mental, neurological and substance use disorders by fostering strong links with the employment, education, social (including housing) and other relevant sectors.

5. Protection of human rights

- Pay special attention to national legislation and international human rights standards (Box 1).
- Promote autonomy and independent living in the community and discourage institutionalization.
- Provide care in a way that respects the dignity of the person, that is culturally sensitive and appropriate, and that is free from discrimination on the basis of race, colour, sex, language, religion, political or other opinion, national, ethnic, indigenous or social origin, property, birth, age or other status.
- Ensure that the person understands the proposed treatment and provides free and informed consent to treatment.
- Involve children and adolescents in treatment decisions in a manner consistent with their evolving capacities, and give them the opportunity to discuss their concerns in private.

6. Attention to overall well-being

- Provide advice about physical activity and healthy body weight maintenance.
- Educate people about harmful alcohol use.
- Encourage cessation of tobacco and substance use.
- Provide education about other risky behaviour (e.g. Unprotected sex).
- Conduct regular physical health checks.
- Prepare people for developmental life changes, such as puberty and menopause, and provide the necessary support.
- Discuss plans for pregnancy and contraception methods with women of childbearing age.

BOX 1
Key international human rights standards

Convention against torture and other cruel, inhuman or degrading treatment or punishment. United Nations General Assembly Resolution 39/46, annex, 39 UN GAOR Supp. (No. 51) at 197, UN Doc. A/39/51 (1984). Entered into force 26 June 1987.
http://www2.ohchr.org/english/law/cat.htm

Convention on the elimination of all forms of discrimination against women (1979). Adopted by United Nations General Assembly Resolution 34/180 of 18 December 1979.
http://www.un.org/womenwatch/daw/cedaw/cedaw.htm

Convention on the rights of persons with disabilities and optional protocol. Adopted by the United Nations General Assembly on 13 December 2006.
http://www.un.org/disabilities/documents/convention/convoptprot-e.pdf

Convention on the rights of the child (1989). Adopted by United Nations General Assembly Resolution 44/25 of 20 November 1989. http://www2.ohchr.org/english/law/crc.htm

International covenant on civil and political rights (1966). Adopted by UN General Assembly Resolution 2200A (XXI) of 16 December 1966.
Http://www2.ohchr.org/english/law/ccpr.htm

International covenant on economic, social and cultural rights (1966). Adopted by UN General Assembly Resolution 2200A (XXI) of 16 December 1966.
Http://www2.ohchr.org/english/law/cescr.htm

MC

mhGAP-IG Master Chart: Which priority condition(s) should be assessed?

1. These common presentations indicate the need for assessment.
2. If people present with features from more than one condition, then all relevant conditions need to be assessed.
3. All conditions apply to all ages, unless otherwise specified.

COMMON PRESENTATION	CONDITION TO BE ASSESSED	GO TO	
• Low energy; fatigue; sleep or appetite problems<br>• Persistent sad or anxious mood; irritability<br>• Low interest or pleasure in activities that used to be interesting or enjoyable<br>• Multiple symptoms with no clear physical cause (e.g. aches and pains, palpitations, numbness)<br>• Difficulties in carrying out usual work, school, domestic or social activities.	Depression*	DEP	10
• Abnormal or disorganized behaviour (e.g. incoherent or irrelevant speech, unusual appearance, self - neglect, unkempt appearance).<br>• Delusions (a false firmly held belief or suspicion)<br>• Hallucinations (hearing voices or seeing things that are not there)<br>• Neglecting usual responsibilities related to work, school, domestic or social activities<br>• Manic symptoms (several days of being abnormally happy, too energetic, too talkative, very Irritable, not sleeping, reckless behaviour)	Psychosis*	PSY	18
• Convulsive movement or fits/seizures<br>• During the convulsion:<br>- loss of consciousness or impaired consciousness<br>- stiffness, rigidity<br>- tongue bite, injury, incontinence of urine or faeces<br>• After the convulsion: fatigue, drowsiness, sleepiness, confusion, abnormal behaviour, headache, muscle aches, or weakness on one side of the body	Epilepsy / Seizures	EPI	32
• Delayed development: much slower learning than other children of same age in activities such as: smiling, sitting, standing walking, talking/communicating and other areas of development, such as reading and writing.<br>• Abnormalities in communication; restricted, repetitive behaviour<br>• Difficulties in carrying out everyday activities normal for that age	Developmental Disorders<br>Children and adolescents	DEP	40

Behavioural Disorders — BEH — 44

Children and adolescents

- Excessive inattention and absent-mindedness, repeatedly stopping tasks before completion and switching to other activities
- Excessive over-activity: excessive running around, extreme difficulties remaining seated, excessive talking or fidgeting
- Excessive impulsivity: frequently doing things without forethought
- Repeated and continued behaviour that disturbs others (e.g. unusually frequent and severe temper tantrums, cruel behaviour, persistent and severe disobedience, stealing)
- Sudden changes in behaviour or peer relations, including withdrawal and anger

Dementia — BEH — 50

Older people

- Decline or problems with memory (severe forgetfulness) and orientation (awareness of time, place and person)
- Mood or behavioural problems such as apathy (appearing uninterested) or irritability
- Loss of emotional control – easily upset, irritable or tearful
- Difficulties in carrying out usual work, domestic or social activities

Alcohol Use Disorders — ALC — 58

- Appearing to be under the influence of alcohol (e.g. smell of alcohol, looks intoxicated, hangover)
- Presenting with an injury
- Somatic symptoms associated with alcohol use (e.g. insomnia, fatigue, anorexia, nausea, vomiting, indigestion, diarrhoea, headaches)
- Difficulties in carrying out usual work, school, domestic or social activities

Drug Use Disorders — DRU — 66

- Appearing drug-affected (e.g. low energy, agitated, fidgeting, slurred speech)
- Signs of drug use (injection marks, skin infection, unkempt appearance)
- Requesting prescriptions for sedative medication (sleeping tablets, opioids)
- Financial difficulties or crime-related legal problems
- Difficulties in carrying out usual work, domestic or social activities

Self-harm / Suicide — SUI — 74

- Current thoughts, plan or act of self-harm or suicide
- History of thoughts, plan or act of self-harm or suicide

* *The **Bipolar Disorder (BPD)** module is accessed through either the Psychosis module or the **Depression** module.*
*The **Other Significant Emotional or Medically Unexplained Complaints (OTH)** module is accessed through the **Depression** module.*

DEP

Depression

Moderate-Severe Depression

In typical depressive episodes, the person experiences depressed mood, loss of interest and enjoyment, and reduced energy leading to diminished activity for at least 2 weeks. Many people with depression also suffer from anxiety symptoms and medically unexplained somatic symptoms.

This module covers moderate-severe depression across the lifespan, including childhood, adolescence, and old age.

A person in the mhGAP category of Moderate-Severe Depression has difficulties carrying out his or her usual work, school, domestic or social activities due to symptoms of depression.

The management of symptoms not amounting to moderate-severe depression is covered within the module on Other Significant Emotional or Medically Unexplained Somatic Complaints. OTH

Of note, people currently exposed to severe adversity often experience psychological difficulties consistent with symptoms of depression but they do not necessary have moderate-severe depression. When considering whether the person has moderate-severe depression, it is essential to assess whether the person not only has symptoms but also has difficulties in day-today functioning due to the symptoms.

Depression

Assessment and Managements Guide

1. Does the person have moderate-severe depression?

» **For at least 2 weeks, has the person had at least 2 of the following core depression symptoms:**
- Depressed mood (most of the day, almost every day), (for children and adolescents: either irritability or depressed mood)
- Loss of interest or pleasure in activities that are normally pleasurable
- Decreased energy or easily fatigued

» **During the last 2 weeks has the person had at least 3 other features of depression:**
- Reduced concentration and attention
- Reduced self-esteem and self-confi dence
- Ideas of guilt and unworthiness
- Bleak and pessimistic view of the future
- Ideas or acts of self-harm or suicide
- Disturbed sleep
- Diminished appetite

» **Does the person have dif? culties carrying out usual work, school, domestic, or social activities?**

YES

If YES to all 3 questions then: **moderate-severe depression** is likely

- » Psychoeducation. » DEP 2.1
- » Address current psychosocial stressors. » DEP 2.2
- » Reactivate social networks. » DEP 2.3
- » Consider antidepressants. » DEP 3
- » If available, consider interpersonal therapy, behavioural activation or cognitive behavioural therapy. » INT
- » If available, consider adjunct treatments: structured physical activity programme » DEP 2.4, relaxation training or problem-solving treatment. » INT
- » DO NOT manage the complaint with injections or other ineffective treatments (e.g. Sleeping tablets and blood tonic).
- » Offer regular follow-up. » DEP 2.5

NO

If NO to some or all of the three questions and if no other priority conditions have been identifi ed on the mhGAP-IG Master Chart

- » Exit this module, and assess for Other signi? cant Emotional or Medically unexplained somatic Complaints » OTH

Check for recent bereavement or other major loss in prior 2 months.

In case of recent bereavement or other recent major loss

Follow the above advice but DO NOT consider antidepressants or psychotherapy as ? rst line treatment. Discuss and support culturally appropriate mourning / adjustment.

Depression

DEP 1

Assessment and Managements Guide

2. Does the person have bipolar depression?

» Ask about prior episode of manic symptoms such as extremely elevated, expansive or irritable mood, increased activity and extreme talkativeness, flight of ideas, extreme decreased need for sleep, grandiosity, extreme distractibility or reckless behaviour. See Bipolar Disorder Module. » BPD

YES

Bipolar depression is likely if the person had:

» 3 or more manic symptoms lasting for at least 1 week OR

» A previously established diagnosis of bipolar disorder

» Manage the bipolar depression. See Bipolar Disorder Module. **» BPD**

NOTE: People with bipolar depression are at risk of developing mania. Their treatment is different!

3. Does the person have depression with psychotic features (delusions, Hallucinations, stupor)?

YES

If YES

» Augment above treatment for moderate-severe depression With an antipsychotic in consultation with a specialist. See Psychosis Module. » PsY

4. Concurrent conditions

» (Re)consider risk of suicide / self-harm (see mhGAP-IG Master Chart)

» (Re)consider possible presence of alcohol use disorder or Other substance use disorder (see mhGAP-IG Master Chart)

» look for concurrent medical illness, especially signs / symptoms suggesting hypothyroidism, anaemia, tumours, stroke, hypertension, diabetes, HIV / AIDS, malaria or medication use, that can cause or exacerbate depression.

YES

If a concurrent condition is present

» Manage both the moderate-severe depression and the concurrent condition.

» Monitor adherence to treatment for concurrent medical illness, Because depression may reduce adherence.

5. Person is female of child-bearing age

Ask about:

- Current known or possible pregnancy
- Last menstrual period, if pregnant
- Whether person is breastfeeding

YES

If pregnant or breastfeeding

Follow above treatment advice for the management of moderate-severe depression, but

- During pregnancy or breast-feeding antidepressants should be avoided as far as possible.
- If no response to psychosocial treatment, consider using lowest effective dose of antidepressants.
- **REFER TO NEXT LEVEL OF CARE**
- If breast feeding, avoid long acting medication such as fluoxetine

If younger than 12 years

- DO NOT prescribe antidepressant medication.
- Provide psychoeducation to parents. » DEP 2.1
- Address current psychosocial stressors. » DEP 2.2
- Offer regular follow-up. » DEP 2.5

If 12 years or older

- DO NOT consider antidepressant as first-line treatment.
- Psychoeducation. » DEP 2.1
- Address current psychosocial stressors. » DEP 2.2
- If available, consider interpersonal psychotherapy (IPT) or cognitive behavioural therapy (CBT), behavioural activation. » INT
- If available, consider adjunct treatments: structured physical activity programme » DEP 2.4, relaxation training or problemsolving treatment. » INT
- When psychosocial interventions prove ineffective, refer to next level of care.
- Offer regular follow-up. » DEP 2.5

5. Person is female of child-bearing age

Depression

DEP 2

Intervention Details

Psychosocial / Non-Pharmacological Treatment and Advice

2.1 Psychoeducation
(for the person and his or her family, as appropriate)

» Depression is a very common problem that can happen to anybody.

» Depressed people tend to have unrealistic negative opinions about themselves, their life and their future.

» Effective treatment is possible. It tends to take at least a few weeks before treatment reduces the depression. Adherence to any prescribed treatment is important.

» The following need to be emphasized:
– the importance of continuing, as far as possible, activities that used to be interesting or give pleasure, regardless of whether these currently seem interesting or give pleasure;
– the importance of trying to maintain a regular sleep cycle (i.e., going to be bed at the same time every night, trying to sleep the same amount as before, avoiding sleeping too much);
– the benefit of regular physical activity, as far as possible;
– the benefit of regular social activity, including participation in communal social activities, as far as possible;
– recognizing thoughts of self-harm or suicide and coming back for help when these occur;
– in older people, the importance of continuing to seek help for physical health problems.

2.2 Addressing current psychosocial stressors

» Offer the person an opportunity to talk, preferably in a private space. Ask for the person's subjective understanding of the causes of his or her symptoms.

» Ask about current psychosocial stressors and, to the extent possible, address pertinent social issues and problem-solve for psychosocial stressors or relationship difficulties with the help of community services / resources.

» Assess and manage any situation of maltreatment, abuse (e.g. domestic violence) and neglect (e.g. of children or older people). Contact legal and community resources, as appropriate.

» Identify supportive family members and involve them as much as possible and appropriate.

» **In children and adolescents:**
– Assess and manage mental, neurological and substance use problems (particularly depression) in parents (see mhGAP-IG Master Chart).
– Assess parents' psychosocial stressors and manage them to the extent possible with the help of community services / resources.
– Assess and manage maltreatment, exclusion or bullying (ask child or adolescent directly about it).
– If there are school performance problems, discuss with teacher on how to support the student.
– Provide culture-relevant parent skills training if available. » INT

2.3 Reactivate social networks

» Identify the person's prior social activities that, if reinitiated, would have the potential for providing direct or indirect psychosocial support (e.g. family gatherings, outings with friends, visiting neighbours, social activities at work sites, sports, community activities).

» Build on the person's strengths and abilities and actively encourage to resume prior social activities as far as is possible.

2.4 Structured physical activity programme
(adjunct treatment option for moderate-severe depression)

» Organization of physical activity of moderate duration (e.g. 45 minutes) 3 times per week.

» Explore with the person what kind of physical activity is more appealing, and support him or her to gradually increase the amount of physical activity, starting for example with 5 minutes of physical activity.

2.5 Offer regular follow-up

» Follow up regularly (e.g. in person at the clinic, by phone, or through community health worker).

» Re-assess the person for improvement (e.g. after 4 weeks).

Antidepressant Medication

3.1 Initiating antidepressant medication

» **Select an antidepressant**
- Select an antidepressant from the National or WHO Formulary. Fluoxetine (but not other selective serotonin reuptake inhibitors (SSRIs)) and amitriptyline (as well as other tricyclic antidepressants (TCAs)) are antidepressants mentioned in the WHO Formulary and are on the WHO Model List of Essential Medicines. See » DEP 3.5
- In selecting an antidepressant for the person, consider the symptom pattern of the person, the side-effect profile of the medication, and the efficacy of previous antidepressant treatments, if any.
- For co-morbid medical conditions: Before prescribing anti-depressants, consider potential for drug-disease or drug-drug interaction. Consult the National or the WHO Formulary.
- Combining antidepressants with other psychotropic medication requires supervision by, or consultation with, a specialist.

» **Tell person and family about:**
- the delay in onset of effect;
- potential side-effects and the risk of these symptoms, to seek help promptly if these are distressing, and how to identify signs of mania;
- the possibility of discontinuation / withdrawal symptoms on missing doses, and that these symptoms are usually mild and self-limiting but can occasionally be severe, particularly if the medication is stopped abruptly. However, antidepressants are not addictive;
- the duration of the treatment, noting that antidepressants are effective both for treating depression and for preventing its recurrence.

3.2 Precautions to be observed for antidepressant medication in special Populations

» **People with ideas, plans or acts of self-harm or suicide**
- SSRIs are first choice.
- Monitor frequently (e.g. once a week).
- To avoid overdoses in people at imminent risk of selfharm / suicide, ensure that such people have access to a limited supply of antidepressants only (e.g. dispense for one week at a time). See Self-harm / Suicide Module. **» SUI**

1

» **Adolescents 12 years and older**
- When psychosocial interventions prove ineffective, consider fluoxetine (but not other SSRIs or TCAs).
- Where possible, consult mental health specialist when treating adolescents with fluoxetine.
- Monitor adolescents on fluoxetine frequently (ideally once a week) for emergence of suicidal ideas during the first month of treatment. Tell adolescent and parent about increased risk of suicidal ideas and that they should make urgent contact if they notice such features.

» **Older people**
- TCAs should be avoided, if possible. SSRIs are first choice.
- Monitor side-effects carefully, particularly of TCAs.
- Consider the increased risk of drug interactions, and give greater time for response (a minimum of 6 – 12 weeks before considering that medication is ineffective, and 12 weeks if there is a partial response within this period).

» **People with cardiovascular disease**
- SSRIs are first choice.
- DO NOT prescribe TCAs to people at risk of serious cardiac arrhythmias or with recent myocardial infarction.
- In all cardio-vascular cases, measure blood pressure before prescribing TCAs and observe for orthostatic hypotension once TCAs are started.

3.3 Monitoring people on antidepressant Medication

» If symptoms of mania emerge during treatment: immediately stop antidepressants and assess for and manage the mania and bipolar disorder. **» BPD**

» If people on SSRIs show marked / prolonged akathisia (inner restlessness or inability to sit still), review use of the medication. Either change to TCAs or consider concomitant use of diazepam (5 – 10 mg / day) for a brief period (1 week). In case of switching to TCAs, be aware of occasional poorer tolerability compared to SSRIs and the increased risk of cardio-toxicity and toxicity in overdose.

» If poor adherence, identify and try to address reasons for poor adherence (e.g. side-effects, costs, person's beliefs about the disorder and treatment).

» If inadequate response (symptoms worsen or do not improve after 4 – 6 weeks): review diagnosis (including co-morbid diagnoses) and check whether medication has been taken regularly and prescribed at maximum dose. Consider increasing the dose. If symptoms persist 4 – 6 weeks at prescribed maximum dose, then consider switching to another treatment (i.e., Psychological treatment **» INT,** different class of antidepressants **» DEP 3.5).**
Switch from one antidepressant to another with care, that is: stop the first drug; leave a gap of a few days if clinically possible; start the second drug. If switching is from fluoxetine to TCA the gap should be longer, for example one week.

Depression

DEP 3

Intervention Details

» If no response to adequate trial of two antidepressant medications or if no response on one adequate trial of antidepressants **CONSULT A SPECIALIST**

3.4 Terminating antidepressant medication

» **Consider stopping** antidepressant medication when the person (a) has no or minimal depressive symptoms for 9 – 12 months and (b) has been able to carry out routine activities for that time period.

» **Terminate contact** as follows:
- In advance, discuss with person the ending of the treatment.
- For TCAs and most SSRIs (but faster for fluoxetine): Reduce doses gradually over at least a 4-week period; some people may require longer period.
- Remind the person about the possibility of discontinuation / withdrawal symptoms on stopping or reducing the dose, and that these symptoms are usually mild and self-limiting but can occasionally be severe, particularly if the medication is stopped abruptly.
- Advise about early symptoms of relapse (e.g. alteration in sleep or appetite for more than 3 days) and when to come for routine follow-up.
- Repeat psychoeducation messages, as relevant. » **DEP 2.1**

» **Monitor and manage antidepressant withdrawal symptoms** (common: dizziness, tingling, anxiety, irritability, fatigue, headache, nausea, sleep problems)
- Mild withdrawal symptoms: reassure the person and monitor symptoms.
- Severe withdrawal symptoms: reintroduce the antidepressant at the effective dose and reduce more gradually.
- **CONSULT A SPECIALIST** if significant discontinuation / withdrawal symptoms persist.

» **Monitor re-emerging depression** symptoms during withdrawal of antidepressant: prescribe the same antidepressant at the previous effective dose for another 12 months if symptoms re-emerge.

Intervention Details

This information is for quick reference only and is not intended to be an exhaustive guide to the medications, their dosing and side-effects. Additional details are given in "Pharmacological Treatment of Mental Disorders in Primary Health Care" (WHO, 2009) (http://www.who.int/mental_health/management/psychotropic/en/index.html).

3.5 Information on SSRIs and TCAs

Selective serotonin Reuptake Inhibitors (ssRIs; e.g. Fluoxetine)

Serious side-effects (these are rare)
- marked / prolonged akathisia (inner restlessness or inability to sit still);
- bleeding abnormalities in those who regularly use aspirin and other non-steroidal anti-inflammatory drugs.

Common side-effects
(most side-effects diminish after a few days; none are permanent)
- restlessness, nervousness, insomnia, anorexia and other gastrointestinal disturbances, headache, sexual dysfunction.

Cautions
- risk of inducing mania in people with bipolar disorder.

Time to response after initiation of adequate dose
- 4 – 6 weeks.

Dosing fluoxetine in healthy adults
- Initiate treatment with 20 mg daily (to reduce risk of side effects that undermine adherence, one may start at 10 mg (e.g. half a tablet) once daily and increase to 20 mg if the medication is tolerated).
- If no response in 4 – 6 weeks or partial response in 6 weeks, increase dose by 20 mg (maximum dose 60 mg) according to tolerability and symptom response.

Dosing fluoxetine in adolescents
- Initiate treatment with 10 mg (e.g. half a tablet) once daily and increase to 20 mg after 1 – 2 weeks (maximum dose 20 mg)
- If no response in 6 – 12 weeks or partial response in 12 weeks, consult a specialist.

Dosing fluoxetine in elderly or medically ill
- Initiate treatment with 10 mg tablet (if available) once daily or 20 mg every other day for 1 – 2 weeks and then increase to 20 mg if tolerated.
- If no response in 6 – 12 weeks or partial response in 12 weeks, increase dose gradually (maximum dose 60 mg). Increase dose more gradually than in healthy adults.

Tricyclic antidepressants (TCAs; e.g. amitriptyline)

Serious side-effects (these are rare)
- cardiac arrhythmia.

Common side-effects
(most side-effects diminish after a few days; none are permanent)
- orthostatic hypotension (fall risk), dry mouth, constipation, difficulty urinating, dizziness, blurred vision and sedation.

Cautions
- risk of switch to mania, especially in people with bipolar disorder;
- impaired ability to perform certain skilled tasks (e.g. driving) – take precautions until accustomed to medication;
- risk of self-harm (lethal in overdose);
- less effective and more severe sedation if given to regular alcohol users.

Time to response after initiation of adequate dose
- 4 – 6 weeks (pain and sleep symptoms tend to improve in a few days).

Dosing amitriptyline in healthy adults
- Initiate treatment with 50 mg at bedtime.
- Increase by 25 to 50 mg every 1 – 2 weeks, aiming for 100 – 150 mg by 4 – 6 weeks depending on response and tolerability.
- If no response in 4 – 6 weeks or partial response in 6 weeks, increase dose gradually (maximum dose 200 mg) in divided doses (or a single dose at night).

Dosing amitriptyline in adolescents
- DO NOT prescribe amitriptyline in adolescents.

Dosing amitriptyline in elderly or medically ill
- Initiate with 25 mg at bedtime.
- Increase by 25 mg weekly, aiming for a target dose of 50 – 75 mg by 4 – 6 weeks.
- If no response in 6 – 12 weeks or partial response in 12 weeks, increase dose gradually (maximum dose 100 mg) in divided doses.
- Monitor for orthostatic hypotension.

Psychosis

PSY

Psychosis is characterized by distortions of thinking and perception, as well as inappropriate or narrowed range of emotions. Incoherent or irrelevant speech may be present. Hallucinations (hearing voices or seeing things that are not there), delusions (fixed, false idiosyncratic beliefs) or excessive and unwarranted suspicions may also occur. Severe abnormalities of behaviour, such as disorganized behaviour, agitation, excitement and inactivity or overactivity, may be seen. Disturbance of emotions, such as marked apathy or disconnect between reported emotion and observed affect (such as facial expressions and body language), may also be detected. People with psychosis are at high risk of exposure to human rights violations.

Psychosis

PSY 1

Assessment and Managements Guide

1. Does the person have acute psychosis?

- Incoherent or irrelevant speech
- Delusions
- Hallucinations
- Withdrawal, agitation, disorganized behaviour
- Beliefs that thoughts are being inserted or broadcast from one's mind
- Social withdrawal and neglect of usual responsibilities related to work, school, domestic or social activities

Ask the person or carer

- When this episode began
- Whether any prior episodes occurred
- Details of any previous or current treatment

Rule out psychotic symptoms due to:

- Alcohol or drug intoxication or withdrawal (Refer to Alcohol use disorder / Drug use disorder module » **ALC** and » **DRU**)
- Delirium due to acute medical conditions such as cerebral malaria, systemic infections / Sepsis, head injury

YES

If multiple symptoms are present, **psychosis** is likely.

If this episode is:
- the first episode OR
- a relapse OR
- worsening of psychotic symptoms

it is an **acute psychotic episode**

- Provide education to the person and carers about psychosis and its treatment. **» PSY 2.1**
- Begin antipsychotic medication. **» PSY 3.1**
- If available, provide psychological and social interventions, such as family therapy or social skills therapy. **» INT**
- Facilitate rehabilitation. **» PSY 2.2**
- Provide regular follow-up. **» PSY 2.3**
- Maintain realistic hope and optimism.

NOTE: **DO NOT** prescribe anticholinergic medication routinely to prevent antipsychotic side-effects.

2. Does the person have chronic psychosis?

YES

If symptoms persist for more than 3 months

chronic psychosis is likely

- Provide education to the person and carers. » pSY 2.1
- **IF THE PERSON IS NOT ON ANY TREATMENT, START TREATMENT AS FOR ACUTE PSYCHOTIC EPISODE.**
- Review and ensure treatment adherence.
- If the person is not responding adequately, consider increasing current medication or changing it. **» PSY 3.1 and 3.2**
- If available, provide psychological and social interventions such as family therapy or social skills therapy. Consider adding a psychosocial intervention not offered earlier, e.g. cognitive behavioural therapy if available. **» INT**
- Provide regular follow-up. **» PSY 2.3**
- Maintain realistic hope and optimism.
- Facilitate rehabilitation. **» PSY 2.2**

Psychosis

PSY 1

Assessment and Managements Guide

3. Is the person having an acute manic episode?

Look for:

- Several days of:
  - Markedly elevated or irritable mood
  - Excessive energy and activity
  - Excessive talking
  - Recklessness
- Past history of:
  - Depressed mood
  - Decreased energy and activity

(see Depression Module for details). » **DEP**

NOTE:

- People who suffer only manic episodes (without depression) are also classified as having bipolar disorder.
- Complete recovery between episodes is common in bipolar disorder.

YES

If yes, this could be bipolar disorder

- Exit this module and go to **Bipolar Disorder Module.** **» BPD**

4. Look for concurrent conditions

- Alcohol use or drug use disorders
- Suicide / self-harm
- Dementia
- Concurrent medical illness: Consider especially signs/symptoms suggesting stroke, diabetes, hypertension, HIV/AIDS, cerebral malaria, typhoid psychosis or medications (e.g. steroids)

YES

If yes, then

- Manage both the psychosis and the concurrent condition.

Woman of child-bearing age?

- In the case of a pregnant woman, liaise with the maternal health specialist, if available, to organize care.
- Explain the risk of adverse consequences for the mother and her baby, including the risk of obstetric complications and psychotic relapse (particularly if medication is changed or stopped).
- Women with psychosis who are planning a pregnancy, pregnant, or breastfeeding should be treated with low-dose oral haloperidol or chlorpromazine.
- Avoid routine use of depot antipsychotics.

Intervention Details

Psychosocial Interventions

2.1 Psychoeducation

» **Messages to the person with psychosis**
- the person's ability to recover;
- the importance of continuing regular social, educational and occupational activities, as far as possible;
- the suffering and problems can be reduced with treatment;
- the importance of taking medication regularly;
- the right of the person to be involved in every decision that concerns his or her treatment;
- the importance of staying healthy (e.g. healthy diet, staying physically active, maintaining personal hygiene).

» **Additional messages to family members of people with psychosis**
- The person with psychosis may hear voices or may firmly believe things that are untrue.
- The person with psychosis often does not agree that he or she is ill and may sometimes be hostile.
- The importance of recognizing the return/worsening of symptoms and of coming back for re-assessment should be stressed.
- The importance of including the person in family and other social activities should be stressed.
- Family members should avoid expressing constant or severe criticism or hostility towards the person with psychosis.
- People with psychosis are often discriminated against but should enjoy the same rights as all people.
- A person with psychosis may have difficulties recovering or functioning in high-stress working or living environments.
- It is best for the person to have a job or to be otherwise meaningfully occupied.
- In general, it is better for the person to live with family or community members in a supportive environment outside hospital settings. Long-term hospitalization should be avoided.

2.2 Facilitate rehabilitation in the community

Involve people with psychosis and their carers actively in the design, implementation and evaluation of these Interventions.

» Coordinate interventions with health staff and with colleagues working in social services, including organizations working on disabilities.

» Facilitate liaison with available health and social resources to meet the family's physical, social and mental health needs.

» Actively encourage the person to resume social, educational and occupational activities as appropriate and advise family members about this. Facilitate inclusion in economic and social activities, including socially and culturally appropriate supported employment. People with psychosis are often discriminated against, so it is important to overcome internal and external prejudices and work toward the best quality of life possible. Work with local agencies to explore employment or educational opportunities, based on the person's needs and skill level.

2.3 Follow-up

» People with psychosis require regular follow-up.

» Initial follow-up should be as frequent as possible, even daily, until acute symptoms begin to respond to treatment. Once the symptoms have responded, monthly to quarterly follow-up is recommended based on clinical need and feasibility factors such as staff availability, distance from clinic, etc.

» Maintain realistic hope and optimism during treatment.

» At each follow-up, assess symptoms, side-effects of medications and adherence. Treatment non-adherence is common and involvement of carers is critical during such periods.

» Assess for and manage concurrent medical conditions.

» Assess for the need of psychosocial interventions at each Follow-up.

Psychosis

PSY 3

Intervention Details

Psychosocial Interventions

3.1 Initiating antipsychotic medications

» For prompt control of acute psychotic symptoms, health-care providers should begin antipsychotic medication immediately after assessment. Consider acute intramuscular treatment only if oral treatment is not feasible. Do not prescribe depot / long-term injections for prompt control of acute psychotic symptoms.

» Prescribe one antipsychotic medication at a time.

» "Start low, go slow": Start with a low dose within the therapeutic range (see the antipsychotic medication table for details) and increase slowly to the lowest effective dose, in order to reduce the risk of side-effects.

» Try the medication at an optimum dose for at least 4 – 6 weeks before considering it ineffective.

» Oral haloperidol or chlorpromazine should be routinely offered to a person with psychotic disorder.

Table: Antipsychotic Medications

Medication:	Chlorpromazine
Starting dose:	50 mg
Typical effective dose (mg):	50 – 100 mg / day
Route:	oral
Significant side-effects:	
Sedation:	+++
Urinary hesitancy:	++
Orthostatic hypotension:	+++
Extrapyramidal side-effects:	+
Neuroleptic malignant Syndrome: ***	rare
Tardive dyskinesia: ****	+
ECG changes:	+
Contraindications:	impaired consciousness, bone marrow depression, Pheochromocytoma

This table is for quick reference only and is not intended to be an exhaustive guide to the medications, their dosing and side-effects. Additional details are given in "Pharmacological Treatment of Mental Disorders in Primary Health Care" (WHO, 2009) (http://www.who.int/mental_health/management/psychotropic/en/index.html).

* Up to 1 g maybe necessary in severe cases.
** Extrapyramidal symptoms include acute dystonic reactions, tics, tremor, and cogwheel and muscular rigidity.
*** Neuroleptic malignant syndrome is a rare but potentially life-threatening disorder characterized by muscular rigidity, elevated temperature and high blood pressure.
**** Tardive dyskinesia is a long-term side-effect of antipsychotic medications characterized by involuntary muscular movements, particularly of the face, hands and trunk.

Intervention Details

Psychosocial Interventions

3.2 Monitoring people on antipsychotic Medication

» **If the response is inadequate to more than one antipsychotic medication using one medicine at a time at adequate dosage for adequate duration:**

– Review diagnosis (and any co-morbid diagnoses).
– Rule out psychosis induced by alcohol or psychoactive substance use (even if it was ruled out initially).
– Ensure treatment adherence; consider depot / long-acting injectable antipsychotic with a view to improve adherence.
– Consider increasing current medication or switching to another medication.
– Consider second-generation antipsychotics (with the exception of clozapine), if cost and availability is not a constraint, as an alternative to haloperidol or chlorpromazine.
– Consider clozapine for those who have not responded to other antipsychotic agents at adequate dosages for adequate duration. Clozapine may be considered by non-specialist health-care providers, preferably under the supervision of mental health professionals. It should only be considered if routine laboratory monitoring is available, because of the risk of life-threatening agranulocytosis.

» **If extrapyramidal side-effects (such as parkinsonism or dystonia) occur:**

– Reduce the dose of antipsychotic medication, and
– Consider switching to another antipsychotic (e.g. switching from haloperidol to chlorpromazine).
– Consider anticholinergic medications for short-term use if these strategies fail or extrapyramidal side-effects are acute, severe or disabling.

Anticholinergic medications

Biperiden, if needed, should be started at 1 mg twice daily, increasing to a target dose of 3 – 12 mg per day, oral or intravenous. Side-effects include sedation, confusion and memory disturbance, especially in the elderly. Rare side-effects include angle-closure glaucoma, myasthenia gravis, gastrointestinal obstruction.

Trihexyphenidyl (Benzhexol) can be used as an alternate medicine at 4 – 12 mg per day. Side-effects are similar to those of biperiden.

3.3 Discontinuation of antipsychotic medications

» **For acute psychosis,** continue antipsychotic treatment for 12 months after full remission.

» **For people with chronic psychosis,** consider treatment discontinuation if the person has been stable for several years, weighing the increased risk of relapse following discontinuation against possible medication side-effects, while taking into account patient preferences in consultation with the family.

» If possible, consult a psychiatrist regarding the decision to discontinue antipsychotic medication.

BPD

Bipolar Disorder

BPD

Bipolar disorder is characterized by episodes in which the person's mood and activity levels are significantly disturbed. This disturbance consists on some occasions of an elevation of mood and increased energy and activity (mania), and on others of a lowering of mood and decreased energy and activity (depression). Characteristically, recovery is complete between episodes. People who experience only manic episodes are also classified as having bipolar disorder.

Bipolar Disorder

BPD 1

Assessment and Managements Guide

1. Is the person in a manic state?

look for:

» Elevated, expansive or irritable mood
» Increased activity, restlessness, excitement
» Increased talkativeness
» Loss of normal social inhibitions
» Decreased need for sleep
» In? ated self-esteem
» Distractibility
» Elevated sexual energy or sexual indiscretion

ask about:

» Symptom duration
» Whether symptoms interfered with usual responsibilities related to work, school, domestic or social activities
» Whether hospitalization was required

YES

if the person has:

» multiple symptoms
» lasting for at least 1 week
» severe enough to interfere significantly with work and social activities or requiring hospitalization

mania is likely

» Begin treatment of acute antipsychotics (Chlopromaxine) and refer to a physician for further management
» Consider a short-term benzodiazepine (such as diazepam) for behavioural disturbance or agitation. **» BPD 3.2**
» Discontinue any antidepressants. **» BPD 3.3**
» Advise the person to modify lifestyle; provide information about bipolar disorder and its treatment. **» BPD 2.1**
» Provide regular follow-up. **» BPD 2.4**

! **if manic symptoms are associated with drug intoxication, refer to Drug use Disorders Module » DRU**

2. Does the person have a known prior episode of mania but now has depression?
(Assess according to Depression Module » Dep)

YES

If YES, then **bipolar depression** is likely.

» Begin treatment with antipsychotics (chlopromaxine) and refer to a physician for further management
» Consider antidepressant combined **with mood stabilizer** for moderate/severe depression according to suggestions in Depression Module »**DEP**

! **Inform the person about the risk of switching to mania before starting antidepressant medication**

» Advise the person to modify lifestyle; provide information about bipolar disorder and its treatment. »**BPD 2.1**
» Reactivate social networks. »**BPD 2.2**
» If available, consider psychological interventions. » **INT**
» Pursue rehabilitation, including appropriate economic and educational activities, using formal and informal systems.»BPD 2.3
»Provide regular follow-up. >> **BPD 2.4**

Bipolar Disorder

BPD 1

Assessment and Managements Guide

3. Look for presence of concurrent conditions

- Alcohol use or drug use disorders
- Dementia
- Suicide / self-harm
- Concurrent medical illness, especially hyper- or hypothyroidism, renal or cardiovascular disease

YES

If YES

- Manage **both** the bipolar and the concurrent condition, except for malaria: provide appropriate treatment and follow-up for malaria-Typhoid fever: provide appropriate treatment and follow-up (I.e one treats the cause and does not add mood stabilizers, although short course of antipsychotics is an option)

4. Is the person not currently manic or depressed but has a history of mania?

YES

This person most likely has bipolar disorder and is currently between episodes.

Relapse prevention is needed if the person has had:

- 2 or more acute episodes (e.g. 2 episodes of mania, or one episode of mania and one episode of depression)
  OR
- a single manic episode involving signifi cant risk and adverse consequences

- If the person is not on a mood stabilizer then begin one. **BPD 4**
- Advise person to modify lifestyle; provide information about bipolar disorder and its treatment. **» BPD 2.1**
- Reactivate social networks. **» BPD 2.2**
- Pursue rehabilitation, including appropriate economic and educational activities, using formal and informal systems. **» BPD 2.3**
- Provide regular follow-up; monitor side-effects and adherence. **» BPD 2.4**

Bipolar Disorder

Assessment and Managements Guide

5. Is the person in a special group?

YES » **pregnant or breast-feeding**

- **Consult a specialist, if available.**
- **Avoid starting treatment with a mood stabilizer.** !
- Consider low-dose haloperidol **(with caution).**
- If a pregnant woman develops acute mania while taking a mood Stabilizer, consider changing to low-dose haloperidol.

YES » **Elderly**

- Use lower doses of medication.
- Anticipate increased risk of drug interactions.

YES » **Adolescent**

- Presenting symptoms may be atypical.
- Take special care to ensure adherence to treatment.
- **CONSULT A SPECIALIST,** if available.

Bipolar Disorder

BPD 2

Intervention Details

Psychosocial Interventions

2.1 Psychoeducation

Messages to people with bipolar disorder (not currently in acute manic state) and to family members of people with bipolar disorder

- **Explanation:** Bipolar disorder is a mental health condition that tends to involve extreme moods, which may go from feeling very depressed and fatigued to feeling extremely energetic, irritated and overly excited.
- There needs to be some **method for monitoring mood,** such as keeping a daily mood log in which irritability, anger or euphoria are recorded.
- It is important to maintain a regular sleep cycle (e.g. Going to bed at the same time every night, trying to sleep the same amount as before illness, avoiding sleeping much less than usual).
- Relapses need to be prevented, by recognizing when symptoms return, such as sleeping less, spending more money or feeling much more energetic than usual, and coming back for treatment when these occur.
- A person in a manic state lacks insight into the illness and may even enjoy the euphoria and improved energy, so carers must be part of relapse prevention.
- Alcohol and other psychoactive substances should be avoided.
- Since lifestyle changes should be continued as long as needed, potentially indefinitely, they should be planned and developed for sustainability.
- The person should be encouraged to **seek support** after significant life events (e.g. bereavement) and to talk to family and friends.
- General coping strategies, such as planning a regular work or school schedule that avoids sleep deprivation, improving social support systems, discussing and soliciting advice about major decisions (especially ones involving money or major commitments) need to be enhanced.
- The family's physical, social and mental health needs should be considered.
- Build rapport: Mutual trust between the person and the health-care staff is critical for a person with bipolar disorder, since a positive therapeutic alliance may improve the longterm outcome, especially by improving treatment adherence.

2.2 Reactivate social networks

- Identify the person's prior social activities that, if reinitiated, would have the potential for providing direct or indirect psychosocial support (e.g. family gatherings, outings with friends, visiting neighbours, social activities at work sites, sports, community activities).
- Actively encourage the person to resume these social activities and advise family members about this.

2.3 Rehabilitation

- Facilitate opportunities for people and their carers to be included in economic, educational and cultural activities appropriate to their cultural environment, using available formal and informal systems.
- Consider supported employment for those who have difficulty obtaining or retaining normal employment.

2.4 Follow-up

- Regular follow-up is required. The relapse rate is high and those in a manic state are often unable to see the need for treatment, so treatment non-adherence is common and involvement of carers is critical during such periods.
- At each follow-up, assess symptoms, side-effects of medications, adherence and the need for psychosocial interventions.
- A person with mania should return for evaluation as frequently as warranted. The evaluation should be more frequent until the manic episode is over.
- Provide information about the illness and treatment to the person and their carers, particularly regarding the signs and symptoms of mania, the importance of regular adherence to medication, even in the absence of symptoms, and the characteristic difficulty the person may sometimes have in understanding the need for treatment. If a person has no carer or person at least to check on them periodically, encourage recruiting someone from the person's community, ideally someone from their network of friends and family.

Intervention Details

Treatment of acute mania

3.1 Lithium, valproate, carbamazepine or Antipsychotics

When appropriate, the primary care physician may consider lithium, valproate, carbamazepine or antipsychotics for treatment of acute mania. Lithium may be considered if clinical and laboratory monitoring are available. If symptoms are severe, consider using an antipsychotic, since onset of effectiveness is more rapid than with mood stabilizers.

For details regarding dose, monitoring, adverse effects, etc, see the section on mood stabilizers in the maintenance treatment of bipolar disorders and the table on mood stabilizers. » **BPD 3**

For details of the use of antipsychotics, refer to the Psychosis Module, pharmacological interventions. » **PSY 3**

3.2 Benzodiazepines

Person in a manic state who is experiencing agitation may benefit from short-term use of a benzodiazepine such as diazepam.

Benzodiazepines should be discontinued gradually as soon as symptoms improve, as tolerance can develop.

3.3 Antidepressants

If mania develops in a person on antidepressants, stop the antidepressants as soon as possible, abruptly or gradually, weighing the risk of discontinuation symptoms (refer to Depression Module, pharmacological interventions, see **» DEP)** against the risk of the antidepressant worsening the mania.

People with bipolar disorder should not receive antidepressants alone because of the risk of inducing mania, particularly with tricyclic antidepressants. Antidepressants are less likely to induce mania when prescribed in conjunction with lithium, antipsychotic therapy or valproate.

3.4 Monitoring

Treatment should be regularly monitored and its effect assessed after 3 and 6 weeks.

If the person has not improved after 6 weeks, consider switching to a medication that has not been tried, or adding another medication in combination therapy, such as an antipsychotic plus a mood stabilizer. If combination therapy proves ineffective, **CONSULT A SPECIALIST.**

Bipolar Disorder

BPD 2

Intervention Details

Maintenance treatment of bipolar disorders

Choosing a mood stabilizer
(lithium, valproate, carbamazepine)

4.1 Lithium

» Consider lithium only if clinical and laboratory monitoring is available.

» Lithium monotherapy is effective against the relapse of both mania and depression, although it is most effective as an antimanic agent.

» Before beginning lithium therapy, obtain renal function tests, thyroid function tests, complete blood count, electrocardiogram and a pregnancy test, if possible.

NOTE: Lithium treatment requires close monitoring of serum level, since the medication has a narrow therapeutic range. In addition, thyroid function must be checked every 6 – 12 months. If laboratory examinations are not available or feasible, lithium should be avoided. Erratic compliance or stopping lithium treatment suddenly may increase the risk of relapse. Do not prescribe lithium where the lithium supply may be frequently interrupted.

» Start with a low dose (300 mg at night) increasing gradually while monitoring the blood concentration every 7 days until it is 0.6 – 1.0 mEq / litre. Once therapeutic blood levels have been achieved, check the blood levels every 2 – 3 months.

» It takes at least 6 months to determine lithium's full effectiveness as maintenance treatment in bipolar disorder.

» Advise the person to maintain fluid intake, particularly after sweating, or if immobile for long periods or febrile.

» Seek medical attention if the person develops diarrhoea or vomiting.

» A person taking lithium should avoid over-the-counter nonsteroidal anti-inflammatory drugs.

» If a severe metabolic or respiratory disturbance occurs, consider stopping lithium treatment for up to 7 days.

4.2 Valproate

» Before beginning valproate treatment, take a history of cardiovascular, renal or hepatic disease.

» Start with a low dose (500 mg / day), increasing (as tolerated) to the target dose.

» Monitor closely for response, side-effects and adherence. Explain the signs and symptoms of blood and liver disorders, and advise the person to seek immediate help if these develop.

» Reduce the dose of medication if intolerable side-effects persist. If reduction in dose does not help, consider switching to another antimanic agent.

4.3 Carbamazepine

» If lithium and valproate are ineffective or poorly tolerated, or if therapy with one of these agents is not feasible, consider carbamazepine.

» Before and during carbamazepine therapy, take a history of cardiovascular, renal or hepatic disease.

» Start with a low dose (200 mg / day at bedtime) and slowly increase until a dose of 600 – 1000 mg / day is achieved.

» Health-care providers should consider that the dose may need to be adjusted after 2 weeks due to hepatic enzyme induction.

» Reduce the dose of medication if intolerable side-effects persist. If reduction in dose does not help, consider switching to another antimanic agent.

Avoid lithium, valproate and carbamazepine in pregnant women, and weigh the risks and benefits in women of childbearing age.

If the person has frequent relapses or continuing functional impairment, consider switching to a different mood stabilizer or adding a second mood stabilizer.
CONSULT A PSYCHIATRIST.

Intervention Details

Maintenance treatment of bipola disorders

4.4 Discontinuation of mood stabilizers

- In a person not currently in a manic or depressed state (bipolar disorder between episodes), follow up every 3 months. Continue treatment and monitor closely for relapse.
- Continue maintenance treatment with the mood stabilizer for at least 2 years after the last bipolar episode.
- However, if a person has had severe episodes with psychotic symptoms or frequent relapses, **CONSULT A PSYCHIATRIST** regarding the decision to discontinue maintenance treatment after 2 years.
- When discontinuing medications, reduce gradually over a period of weeks or months.
- If switching to another medication, begin that medication first and treat with both medications for 2 weeks before tapering off the first medication.

Table: Mood Stabilizers

This table is for quick reference only and is not intended to be an exhaustive guide to the medications, their dosing and side-effects. Additional details are given in "Pharmacological Treatment of Mental Disorders in Primary Health Care" (WHO, 2009) (http://www.who.int/mental_health/management/psychotropic/en/index.html).

Medication:	Lithium	Valproate	Carbamazepine
starting dose (mg):	300	500	200
typical effective dose (mg):	600 – 1200	1000 – 2000	400 – 600
Route:	oral	oral	oral
Target blood level:	0.6 – 1.0 mEq / litre<br>mania: 0.8 – 1.0 mEq / litre;<br>maintenance: 0.6 – 0.8 mEq / litre.<br>*Regular serum level monitoring is critical.*	Not routinely recommended	Not routinely recommended
Noteworthy side effects:	Impaired coordination, polyuria, polydypsia, cognitive problems, cardiac arrhythmias, diabetes insipidus, hypothyroidism	Caution if there is underlying hepatic disease. Hair loss and, rarely, pancreatitis are possible.	Diplopia, impaired coordination, rash, liver enzyme elevations; Rarely: Stevens-Johnson syndrome, aplastic anaemia.
Sedation:	++	++	++
Tremor:	++	++	++
Weight gain:	++	++	++
Hepatotoxicity:	-	++	+
Thrombocytopenia:	-	+	+
Leucopoenia, mild Asymptomatic:	-	+	+

EPI

Epilepsy/Seizures

Epilepsy is a chronic condition, characterized by recurrent unprovoked seizures. It has several causes; it may be genetic or may occur in people who have a past history of birth trauma, brain infections or head injury. In some cases, no specific cause can be identified. Seizures are caused by abnormal discharges in the brain and can be of different forms; people with epilepsy can have more than one type of seizure. The two major forms of seizures are convulsive and non-convulsive. Non-convulsive epilepsy has features such as change in awareness, behaviour, emotions or senses (such as taste, smell, vision or hearing) similar to mental health conditions, so may be confused with them. Convulsive epilepsy has features such as sudden muscle contraction, causing the person to fall and lie rigidly, followed by the muscles alternating between relaxation and rigidity, with or without loss of bowel or bladder control. This type is associated with greater stigma and higher morbidity and mortality. This module covers only convulsive epilepsy.

Epilepsy/Seizures

Assessment and Management Guide >> for Emergency Cases

ASSESSMENT AND MANAGEMENT IN EMERGENCY CASES SHOULD PROCEED SIMULTANEOUSLY!

1. Is the person convulsing or Unconscious?

Measure
- Blood pressure, temperature and respiratory rate

Look for:
- Signs of serious head and spine trauma
- Pupils: dilated or pinpoint? not equal? not reactive to light?
- Signs of meningitis
- Focal deficits

Ask about:
- If unconscious, ask accompanying person: "Has there been a recent convulsion?"
- Duration of impaired consciousness / convulsion
- Number of convulsions
- History of head trauma or neck injury
- Other medical problems, medications or poisons (e.g. organophosphate poisoning), substance use (such as stimulant intoxication, benzodiazepine or alcohol withdrawal **» ALC and » DRU)**
- A history of epilepsy

YES — In all cases

- Check airway, breathing and circulation.
- Protect person from injury: make sure they are in a safe place away from fi re or other things that might injure them.
- DO NOT leave the person alone. Seek help if possible.
- Put the person on their side to prevent aspiration.
- DO NOT put anything into the mouth.

YES — If they are convulsing

- Give diazepam rectally (when available). Ideally, an iv route is preferred (at same dose) if an authorized person is available
- Give glucose i.v. (5ml of 50% glucose in adults; 2-5ml/kg of 10% glucose in children).
- Give diazepam i.v. 10mg slowly (child: 1mg/year of age)
- Give diazepam rectally (same dose as above) if i.v. line is difficult to establish.
- DO NOT give intramuscular (i.m.) diazepam
- ! If convulsion does not stop after 10 minutes of fi rst dose of diazepam, give second dose of diazepam or lorazepam (same dose as fi rst) and **REFER PERSON URGENTLY TO HOSPITAL.**
- DO NOT give more than two doses of diazepam.

Rule out pregnancy

If in second half of pregnancy or up to 1 week post partum **AND** no past history of epilepsy, **suspect eclampsia**

- Give magnesium sulfate 10 g i.m.: Give 5 g (10 ml of 50 % solution) i.m. deep in upper outer quadrant of each buttock with 1 ml of 2 % lignocaine in the same syringe.
- If diastolic blood pressure is > 110 mmHg: Give hydralazine 5 mg i.v. Slowly (3 – 4 minutes). If i.v. is not possible, give i.m. If diastolic blood pressure remains > 90 mmHg, repeat the dose at 30 minute intervals until diastolic blood pressure is around 90 mmHg. Do not give more than 20 mg in total. **REFER PERSON URGENTLY TO HOSPITAL** and follow local guidelines for management of pregnancy, childbirth and postpartum care.

YES — If head or neck injury, or neuroinfection Suspected

- Manage the convulsions as above. **REFER PERSON URGENTLY TO HOSPITAL.**
- Head or neck injury: DO NOT move neck because of possible cervical spine injury. Log-roll person when moving.
- Neuroinfection: Manage the infection according to local guidelines.

Epilepsy/Seizures

EPI 1

Assessment and Management Guide >> for Emergency Cases

2. Are convulsions still continuing?

If convulsions

» last for more than 30 minutes OR

» occur so frequently that the person does not recover consciousness between convulsions OR

» are not responsive to two doses of diazepam

SUSPECT STATUS EPILEPTICUS

REFER PERSON URGENTLY TO HOSPITAL and manage in hospital.

» Check airway, breathing, circulation.
» Administer oxygen.
» Check need for intubation / ventilation when multiple doses of various medications are being administered.
» Put the person on their side to prevent aspiration
» DO NOT put anything into the mouth during a convulsion.

A doctor to give:

» Phenobarbital 10-15mg/kg, i.v. (rate of 100mg/minute).
OR
» Phenytoin 15-18mg/kg i.v. (through different line to diazepam) over 60 minutes. It is critical to have a very good i.v. line as the drug is very caustic and will cause significant local damage if it extravasates. (Patient's vital signs to be manually monitored during administration)

If seizures continue:

» Give the other drug (if available) **OR** additional phenytoin 10 mg / kg i.v. (through different line to diazepam) Over 30 minutes.
» Monitor the person for respiratory depression.

ASSESSMENT AND MANAGEMENT IN EMERGENCY CASES SHOULD PROCEED SIMULTANEOUSLY!

Epilepsy/Seizures

Assessment and Management Guide

1. Does the person have convulsive seizures?

Ask about the following criteria:

- Loss of or impaired consciousness
- Stiffness, rigidity lasting longer than 1 – 2 minute
- Convulsive movements lasting longer than 1 – 2 minute
- Tongue bite or self-injury
- Incontinence of urine and / or faeces
- After the abnormal movement: fatigue, drowsiness, sleepiness Confusion, abnormal behavior, headache or muscles aches

NO

If person experiences 2 or fewer criteria suspect non-convulsive seizures or other medical Condition.

- Follow up after 3 months
- In case of recurrent episodes, **CONSULT OR REFER** To the next level of care

YES

If person experiences convulsive movements and 2 other criteria, these could be convulsive seizures and could have an acute cause or be due to Epilepsy.

2. If convulsive seizures are present, Do they have an acute cause?

Ask and look for:

- Fever, headache, signs of meningeal irritation, e.g. stiff neck
- If they started immediately after head injury
- Metabolic abnormality (hypoglycaemia, hyponatraemia)
- Substance use or withdrawal

YES

Suspect an acute etiology

- Neuroinfection (meningitis / encephalitis)
- Cerebral malaria
- Head injury
- Hypoglycaemia or hyponatraemia
- Substance use / withdrawal

→

- Treat the medical condition
- Antiepileptic maintenance treatment is not required.
- Follow up after 3 months to assess if the person has Epilepsy.

IF YES and a child aged 6 months to 6 years with fever and seizures have one of the following 3 criteria:

- Focal – starts in one part of the body
- Prolonged – more than 15 minutes
- Repetitive – more than 1 episode during the current illness

→

Suspect **complex febrile Seizure.**

Refer for hospital admission.

- Look for neuroinfection (suspect cerebral malaria in high endemic settings).
- Follow up.

If none of the 3 criteria present in a febrile child

→

Suspect **simple febrile seizure**

- Manage fever and look for its cause according to the local IMCI guidelines.
- Observe for 24 hours.
- Follow up.

NO

If no acute cause

Epilepsy/Seizures

Assessment and Management Guide

If no acute cause

3. Has the person had at least 2 convulsive seizures in the last year On 2 different days?

Ask about:

- Severity:
  - How often do they occur?
  - How many did they have in the last year?
  - When was the last episode?
- Possible etiology of the epilepsy (any history of birth asphyxia or trauma, head injury, infection of the brain, family history of seizures)

NO

If there is no clear cause and the person had a single convulsive Seizure

Not epilepsy

- Maintenance of antiepileptic drugs is not required.
- Follow up after 3 months. If there are additional abnormal movements suggestive of a seizure, assess for possible epilepsy.

YES

If yes, **consider epilepsy**

- Initiate antiepileptic drug » **EPI 2.1;** either phenobarbital, phenytoin, carbamazepine or valproate. » **EPI 2.3**
- Educate about condition, lifestyle and safety issues, and importance of adherence and regular follow-up. » **EPI 3.1**
- Follow up regularly. » **EPI 2.2**

Assessment and Management Guide

4. Person in a special group

In children, look for presence of associated intellectual disability (see Developmental Disorders Module) » **DEV** or behavioural problems (see Behavioural Disorders Module) » **BEH**

YES — If associated with **intellectual disability or behavioural** Disorders

» If available, consider carbamazepine or valproate **» EPI 2.3** (avoid phenobarbital and phenytoin)
» Manage associated intellectual disability. » **DEV** or behavioural disorder. » **BEH**

Women of child bearing age

» Advise folate (5 mg / day) in all women of child bearing age.
» Avoid valproate in all women of child bearing age.

YES — If **pregnant**

» Avoid valproate in pregnant women.
» Avoid polytherapy in pregnant women.
» Advise delivery in a hospital.
» At delivery, give 1 mg vitamin K i.m. to the newborn to prevent haemorrhagic disease of the newborn.

YES — If **breastfeeding**

» Initiate either phenobarbital, phenytoin, carbamazepine or valproate, or continue antiepileptic drug therapy if this Has already started.

5. Concurrent conditions

Consider possible presence of **depression, psychosis** or self-harm (see mhGAP-IG Master Chart)

YES — If a concurrent condition is present

» Manage both the epilepsy and the concurrent Condition.

Epilepsy/Seizures

EPI 2

Intervention Details

Pharmacological Treatment and Advice

2.1 Initiate Antiepileptic Drug Therapy

The vast majority of seizures can be controlled (stopped or significantly reduced) by antiepileptic drugs.

- Prescribe phenobarbital, phenytoin, carbamazepine or valproate, based on country-specific availability.
- Start treatment with only one antiepileptic drug.
- Initiate treatment with the lowest dose and build up slowly until complete seizure control is obtained.
- The aim of treatment is to achieve the lowest maintenance dose that provides complete seizure control.
- Tell person and family about:
  - the delay in onset of effect and the time course of treatment;
  - potential side-effects and the risk of these symptoms; to seek help promptly if these are distressing;
  - the risk of abrupt discontinuation / withdrawal symptoms on missing doses;
  - the need for regular follow-up.
- Ask person and the family to keep a simple seizure diary.
- For co-morbid medical conditions: Before prescribing antiepileptic drug, consider potential for drug-disease or drug-drug interaction. Consult the National or the WHO Formulary.

2.2 Follow-up

- Ask and look for symptom response, adverse effects and adherence.
- The adverse effects could be because of high dose of the antiepileptic drug (such as drowsiness, nystagmus, diplopia and ataxia) or these could be idiosyncratic effects (such as allergic reactions, bone marrow depression, hepatic failure). For details of individual antiepileptic drugs, see » EPI 2.3
- The correct antiepileptic drug dose is the smallest one at which complete seizure control is achieved without adverse effects.
- If there are dose-determined adverse effects, decrease the dose of the medication. In case of idiosyncratic reactions, stop the antiepileptic drug the person is on and switch to any of the other antiepileptic drugs.
- If response is poor (less than 50% reduction in frequency of seizures) despite good adherence, increase to maximum tolerated dose.
- If response still poor, try monotherapy with another drug. Start the second drug and build up to an adequate or maximum-tolerated dose and only then taper slowly off the fi rst drug.
- **If seizures are very infrequent and higher doses of medications produce side-effects, less than complete seizure freedom may be the goal.**
- If seizures continue after attempts with two mono-therapies, Review diagnosis (including co-morbidity), treatment adherence And, if necessary, **refer to neurologist or psychiatrist.**
- If there are adverse effects or response is poor, follow up monthly.
- Continue to meet every 3 months if seizures are well controlled.

How and when to stop antiepileptic drugs

- No seizures within the last 2 years.
- Discuss decision with person / carer, weighing up the risk of the seizures starting again.
- In some cases of epilepsy, long-term antiepileptic drug therapy might be required, e.g. in cases of epilepsy secondary to head trauma or neuroinfections, or if the seizures were diffi cult to control.
- Reduce treatment gradually over 2 months.

Intervention Details

2.3 Pharmacological Treatment and Advice

Phenobarbital

- Only needs to be given once a day.
- Give at bedtime (reduces drowsiness during the day).

  Adults: Initiate with 1 mg / kg / day (60 mg tablet) for 2 weeks. If poor response, increase to 2 mg / kg / day (120 mg) for 2 months. If seizures persist, increase the dose to 3 mg / kg / day (180 mg).

  Children: Initiate with 2 mg / kg / day for 2 weeks. If poor response increase the dose to 3 mg / kg / day for 2 months. If seizures persist, increase the dose to maximum of 6 mg / kg / day.

- Continuous administration for 14 – 21 days is needed to obtain steady levels of phenobarbital in the blood; therefore occurrence of seizures during this period should not be considered as treatment failures.
- Side-effects: Dose-determined: drowsiness, lethargy, and hyperactivity in children; idiosyncratic: skin rash, bone marrow depression, hepatic failure.

Carbamazepine

- Give the medication twice daily.
- Steady state is reached in up to 8 days.
- Side effects: allergic skin reactions (which can be severe); blurred vision, diplopia (double vision), ataxia (staggering gait) and nausea: the latter symptoms are usually seen at the start of treatment or at high doses.

Phenytoin

- In children ,give twice daily; in adults , it can be given once daily.
- Small dose increments may lead to big changes in concentration, therefore, increments should be by 25 – 30 mg.
- Dose related: drowsiness, ataxia and slurred speech, motor twitching and mental confusion, coarsening of facial features, gum hyperplasia and hirsutism (uncommon); idiosyncratic: anaemia and other haematological abnormalities, hypersensitivity reactions including skin rash, hepatitis.

Sodium valproate

- Different preparations are available; usually given 2 or 3 times daily.
- Side-effects: sedation and tremor (dose-determined), transient hair loss (re-growth normally begins within 6 months), increase in body weight, impaired hepatic function (idiosyncratic).

	Child		Adolescent / adult	
	**Starting dose**	**Maintenance dose**	**Starting dose**	**Maintenance dose**
Carbamazepine	5 mg / kg / day	10 – 30 mg / kg / day	100 – 200 mg / day	400 – 1400 mg / day
Phenobarbital	2 – 3 mg / kg / day	2 – 6 mg / kg / day	60 mg / day	60 – 180 mg / day
Phenytoin	3 – 4 mg / kg / day	3 – 8 mg / kg / day (Maximum 300 mg daily)	150 – 200 mg / day	200 – 400 mg / day
Sodium valproate	15 – 20 mg / kg / day	15 – 30 mg / kg / day	400 mg / day	400 – 2000 mg / day

Epilepsy/Seizures

EPI 3

Intervention Details

Psychosocial Treatment and Advice

3.1 Provide education to people with seizures / epilepsy and carers

Explain:

- What is a seizure / epilepsy (e.g. "A seizure or fit is a problem related to the brain.
- Epilepsy is an illness involving recurrent seizures
- Epilepsy is not a contagious disease and Epilepsy is not caused by witchcraft or spirits").
- The nature of the person's seizure and its possible cause.
- That it is a chronic condition, but seizures can be fully controlled in 75 % of individuals, after which they may live without medication for the rest of their lives.
- Different treatment options.
- Reasons for referral (when applicable).

Precautions to be taken at home during seizures:

- Lay the person down, on their side, with their head turned to the side to help with the breathing and prevent aspirating secretions and vomit.
- Make sure that the person is breathing properly.
- Do not try to restrain or put anything in the person's mouth.
- Stay with the person until the seizure stops and they wake up.
- Sometimes people with epilepsy know or feel that the seizures are coming. In that case they should lie down somewhere safe to protect themselves from falling.
- Remember that epilepsy is not contagious, so no one will catch seizures by helping.

Explore lifestyle issues:

- People with epilepsy can lead normal lives. They can marry and have children.
- Parents should never remove children with epilepsy from school.
- People with epilepsy can work in most jobs. However they should avoid certain jobs such as working with or near heavy machinery.
- People with epilepsy should avoid cooking on open fires and swimming alone.
- People with epilepsy should avoid excessive alcohol and any recreational substances, sleeping much less than usual or going to places where there are flashing lights.
- Patient should be educated on safety pre-cautions such as driving, working with machinery, swimming or cooking alone.

Development Disorders

Developmental disorder is an umbrella term covering disorders such as intellectual disability / mental retardation as well as pervasive developmental disorders including autism. These disorders usually have a childhood onset, impairment or delay in functions related to central nervous system maturation, and a steady course rather than the remissions and relapses that tend to characterize many other mental disorders. Despite a childhood onset, the developmental disorders tend to persist into adulthood. People with developmental disorders are more vulnerable to physical illness and to develop other priority conditions mentioned in the mhGAP-IG and require additional attention by health-care providers.

Intellectual disability

Intellectual disability is characterized by impairment of skills across multiple developmental areas (i.e., cognitive, language, motor and social) during the developmental period. Lower intelligence diminishes the ability to adapt to the daily demands of life. Intelligence Quotient (IQ) tests can provide guidance to the person's abilities, but should be used only if the tests have been validated for use in the population in which they are being applied.

Pervasive developmental disorders including autism

The features are impaired social behaviour, communication and language, and a narrow range of interests and activities that are both unique to the individual and carried out repetitively. They originate in infancy or early childhood. Usually, but not always, there is some degree of intellectual disability. Behaviours mentioned above are often seen in individuals with intellectual disabilities as well.

Development Disorders

Assessment and Management Guide

1. Does the child have a delay In development?

Assess child's development using local developmental milestones or comparing the child with other children of the same age in the same country.

(For example determine the age at which the child started smiling, sitting up, standing up alone, walking, talking, understanding instructions, and communicating with others.)

For older children *in addition to the above, note how they are managing school work or everyday household activities.*

Look for:

» Oddities in communication (e.g. lack of social usage of the language skills, lack of flexibility in language usage)
» Restricted, repetitive (stereotyped) patterns of behaviour, interests, and activities
» The time, sequence and course of these features
» Loss of previously acquired skills
» Family history of developmental disorder
» Presence of visual and hearing impairment
» Associated epilepsy
» Associated signs of motor impairment or cerebral palsy

YES

If there is a **delay in development** or the mentioned **oddities in communication or Behaviour**

Are there any nutritional deficiencies including iodine deficiency and / or medical conditions?

YES

» Manage nutrition problems, including iodine deficiency and medical conditions, using IMCI guidelines.

IN ALL CASES

» Start family psychoeducation. >>**DEV 2.1**
» Consider parent skills training, when available. >>**INT**
» Inform about available educational and social services and collaborate with them.
» Contact person's school after receiving person and carer consent and provide advice. >>**DEV 2.2**
» Assess current level of adaptive functioning by referring to a psychiatrist if available.
» Manage associated conditions such as visual and hearing impairment.
» Provide support for anticipated difficult situations in life.
» Facilitate and collaborate with community-based rehabilitation services. >>**DEV 2.3**
» Help promote and protect the human rights of the child and the family. >>**DEV 2.4**
» Provide support for carers. >> **DEV 2.5**
» Refer to a pediatrician if available, for further etiological assessment
» Follow up regularly. >>**DEV 2.6**

Development Disorders

DEV 1

Assessment and Management Guide

2. Is the delay in development due to non-stimulating environment or Maternal depression?

YES

If non-stimulating environment or mother with depression

» Provide family psychoeducation » **DEV 2.1** and train parents on how to provide a stimulating environment for the child.
» Treat maternal depression. See Depression Module » **DEP** and other WHO documents for maternal depression and early childhood development.

3. Look for another priority mental, neurological, or substance use disorder *(see mhGAP-IG Master **Chart**).*

Consider especially:

» Epilepsy
» Depression
» Behavioural disorders

YES

If YES

» **Treat according to relevant modules**
– Epilepsy » EPI
– Depression » DEP
– Behavioural disorders » BEH

Use care when prescribing psychotropic medication to people with developmental disorders as they are often more sensitive to their side-effects.

4. Does the child have problem Behaviours?

YES

If YES

» Manage any underlying treatable acute physical problem exacerbating behavioural problems.
» Consider additional family psychoeducation (See Behavioural Disorders Module) » BEH and parent skills training for the specifi c problem behaviour. » INT
» Consider cognitive behavioural therapy (CBT), if trained resources are available » INT
» Provide further support for carers » DEV 2.5

Intervention Details

Psychosocial Treatment and Advice

2.1 Family psychoeducation

Psychoeducation involves the person with developmental disorder and the family, depending on the severity of the condition and availability and significance of the family member role in daily life. Parent or significant family member needs to be trained to:

» Accept and care for the child with developmental disorder.

» Learn what is stressful to the child and what makes them happy; what causes their problem behaviours and what prevents them; what are the child's strengths and weaknesses and how best they learn.

» Understand that people with developmental disorders may have difficulties when they face new situations.

» Schedule the day in terms of regular times for eating, playing, learning and sleeping.

» Involve them in everyday life, starting with simple tasks one at A time.

» Keep them in schools as far as possible; attending mainstream schools even if it is part time is preferable.

» Be careful about their general hygiene and train them in self-care.

» Reward their good behaviour after the act and give no reward when the behaviour is problematic. » BEH 2.1

» Protect them from abuse.

» Respect their right to have a safety zone within a visible boundary where they can feel safe, comfortable and move around and play freely the way they like.

» Communicate and share information with other parents who have children with similar conditions.

2.2 Advice to teachers

» **Make a plan on how to address the child's special educational needs. Simple tips include:**

– Ask the child to sit at the front of the class.
– Give the child extra time to understand assignments.
– Break long assignments into smaller pieces.
– Look for bullying and take appropriate action to stop it.

2.3 Community-based rehabilitation (CBR)

CBR focuses on involving the communities in enhancing the quality of life for people with disabilities and their families, meeting basic needs and ensuring social integration and participation. CBR gives people with disabilities access to rehabilitation in their own communities using predominantly local resources. It is carried out in homes, schools and other central places in the community. It is implemented through the combined efforts of children with developmental disorders, their families and communities, and appropriate health, educational, vocational and social services. It involves promoting a climate that respects and protects the rights of people with disabilities, training the child, empowering families, helping to reduce stress, and improve coping with the developmental disorder. Interventions may range from activities of daily living, school referral, vocational training and parental support.

2.4 Promoting and protecting the human rights of the child and the family

» Review the general principles of care. Be vigilant about issues of human rights and dignity. For example:

– Do not start interventions without informed consent; prevent maltreatment.
– Avoid institutionalization.
– Promote access to schooling and other forms of education.

2.5 Support for carers

» Identify psychosocial impact on carers.
» Assess the carer's needs and promote necessary support and resources for their family life, employment, social activities and health. Arrange for respite care, which means a break now and then when other trustworthy caregivers take over Temporarily.

Developmental Disorders

DEV 2

Intervention Details

Psychosocial Treatment and Advice

2.6 Follow-up

» **Follow up regularly.**

» **For early childhood use IMCI guidelines for follow-up.**

» **Referral criteria:**
– if you notice no improvement or further deterioration in development And / or b ehaviour;
– if you predict danger to the child or others;
– if physical health is affected (such as nutrition problems).

Where to refer

» If the person (a) meets the above referral criteria and (b) has symptoms consistent with intellectual disability, characterized by general delay across multiple developmental areas, such as cognitive, language, motor and social, then:
– Avoid institutionalized care.
– Refer to outpatient intellectual disability specialized services, if available.
– Refer to outpatient speech / language therapy services, if available.

» If the person (a) meets the above referral criteria and has (b) symptoms consistent with a pervasive developmental disorder, characterized by problems in social interaction, communication and behaviour (restricted / repetitive), then:

– Avoid institutionalized care.
– Refer to outpatient specialized services for pervasive developmental disorders or autism if available.

Prevention of Developmental Disorders

» Provide care, prevent and treat malnutrition and infection in pregnant women. Provide safe delivery and care for the newborn after delivery. Prevent head injury, neuroinfections and malnutrition in infants and young children.

» Identify the poorly nourished and frequently ill and other groups of children who are at risk and provide care.Arrange meetings with parents or visit their homes.

» Train parents on how to improve mother-infant interactions and how to provide psychosocial stimulation to the child.

» In case you fi nd that the mother has depression, start treatment of the mother preferably through psychosocial interventions.

» Offer additional psychosocial support to mothers with depression or with any other mental, neurological or substance use condition. This may include home visiting, psychoeducation, improving mothers' knowledge on child rearing practices.

» Women who are pregnant, or planning to become pregnant, should consider avoiding any alcohol consumption, as the developing brain is particularly sensitive to the effects of alcohol, even in the fi rst few weeks post-conception.

Further WHO resources:

» IMCI : http://www.who.int/child_adolescent_health/topics/prevention_care/child/imci/en/index.html

Behavioural Disorders

“Behavioural disorders should take into account the child or adolescent’s developmental level and duration of problem behaviours (at least 6 months). “Behavioural disorders” is an umbrella term that includes more specific disorders, such as hyperkinetic disorder or attention deficit hyperactivity disorder (ADHD) or other behavioural disorders. Behavioural symptoms of varying levels of severity are very common in the population. Only children and adolescents with a moderate to severe degree of psychological, social, educational or occupational impairment in multiple settings should be diagnosed as having behavioural disorders. For some children with behavioural disorders, the problem persists into adulthood.

Hyperkinetic disorder / attention deficit hyperactivity disorder (ADHD)

The main features are impaired attention and overactivity. Impaired attention shows itself as breaking off from tasks and leaving activities unfinished. The child or adolescent shifts frequently from one activity to another. These deficits in persistence and attention should be diagnosed as a disorder only if they are excessive for the child or adolescent’s age and intelligence, anwd affect their normal functioning and learning. Overactivity implies excessive restlessness, especially in situations requiring relative calm. It may involve the child or adolescent running and jumping around, getting up from a seat when he or she was supposed to remain seated, excessive talkativeness and noisiness, or fidgeting and wriggling. The characteristic behavioural problems should be of early onset (before age 6 years) and long duration (> 6 months), and not limited to only one setting.

Other behavioural disorders

Unusually frequent and severe temper tantrums and persistent severe disobedience may be present. Disorders of conduct may be characterized by a repetitive and persistent pattern of dissocial, aggressive or defiant conduct. Such behaviour, when at its most extreme for the individual, should be much more severe than ordinary childish mischief or adolescent rebelliousness. Examples of the behaviours may include: excessive levels of fighting or bullying; cruelty to animals or other people; fire-setting; severe destructiveness to property; stealing; repeated lying and running away from school or home. Judgements concerning the presence of other behavioural

Behavioural Disorders

Assessment and Management Guide

1. Does the person have problems with Inattention and overactivity?

Ask person and carer about:

» Inattention
» Premature breaking off from tasks
» Leaving tasks unfinished
» Frequent changing from activity to activity
» Hyperkinesis:
– Over-activity (excessive for the context or situation)
– Difficulty sitting still
– Excessive talking or noisiness
– Fidgeting or wriggling
» Age of onset, and persistence in different settings: They may have trouble with parents, teachers, siblings, peers, or in all domains of function. If the problems are only in one domain consider causes specifi c to that domain.

» **Assess if the symptoms are appropriate for the child's developmental level.**

» **Explore the impact of:**
– Social, familial and educational or occupational factors
– Medical problems

RULE OUT:

» **Medical conditions or other priority conditions that can potentially cause behavioural disorders** (e.g. Hyperthyroidism, depression or alcohol or drug use)

YES

If several symptoms are present and

» Persist in multiple settings
» Exceed those of other children of the same age and intelligence level
» Started before age 6
» Lasted at least 6 months
» Cause signifi cant disruption in child functioning consider **Attention De? Cit Hyperactivity Disorder (ADHD)**

» Provide family psychoeducation. » BEH 2.1
» Consider parent skills training, when available. » INT
» Contact the person's teacher (if the person goes to school and consent is given by the person and carer), provide advice and plan for special educational needs. » BEH 2.2
» Consider psychosocial interventions such as cognitive behavioural therapy (CBT) and social skills training based on availability. » INT
» Support family to handle social and familial problems affecting child's behaviour. When available, use problem-solving techniques. » INT
» Assess carers regarding the impact of behavioural disorders and offer them support for their personal, social And mental health needs. » BEH 2.3
» If the carer has depression, use the relevant module of mhGAP-IG. » DEP

» CONSULT A SPECIALIST for methylphenidate » BEH 3
Only if the:
– above interventions fail
– child has been carefully assessed
– child is at least 6 years old.
» **Methylphenidate should NOT be used if child is younger than 6.**
» Monitor side-effects and potential for misuse and diversion.
» Record prescription details and side-effects regularly and Carefully in the case notes.

Behavioural Disorders

Assessment and Management Guide

2. Does the person show evidence of Other behavioural disorders?

Ask about:

» Repeated and persistent dissocial, aggressive, or defiant conduct, e.g.
- Excessive levels of fighting or bullying
- Cruelty to animals or other people
- Severe destructiveness to property
- Fire-setting
- Stealing
- Repeated lying
- Truancy from school
- Running away from home
- Frequent and severe temper tantrums
- Defiant provocative behaviour
- Persistent severe disobedience

» The age of onset of symptoms and the duration

Assess if the symptoms are appropriate for the child's developmental level.

Explore the impact of:

» Social, familial and educational or occupational factors

» Medical problems

» Alcohol or drug use

RULE OUT:

» Medical conditions or other priority conditions that can potentially cause behavioural disorders (e.g. hyperthyroidism, depression or alcohol or drug use)

YES

If several symptoms are present and

» are much more severe than ordinary childish mischief or adolescent rebelliousness

» have lasted at least 6 months

» are inappropriate for the child's developmental level

» are not only in response to severe social, family or educational stressors consider a diagnosis of other behavioural disorders

» Provide family psychoeducation. » BEH 2.1

» Consider parent skills training, when available. » INT

» Contact person's teacher (if person goes to school and consent is given by the person and carer), provide advice and plan for special educational needs. » BEH 2.2

» Anticipate major life changes (such as puberty, starting school, or birth of a sibling) and arrange personal and social support.

» Consider psychosocial interventions such as CBT and social skills training based on availability. » INT

» Assess carers regarding the impact of behavioural disorders and offer them support for their personal, social and mental health needs. » BEH 2.3.

» DO NOT use medicines for behavioural disorders in children and adolescents. Consider methylphenidate only under conditions mentioned above for hyperkinetic Disorder.

3. Is the person's problem behaviour a Reaction to fear or trauma?

Explore:

- Is the child being bullied or harmed outside the home?
- Is there any concern about injury or threat within the home?
- Do the parents have any priority conditions for which they require services? (see mhGAP-IG Master Chart)

YES

If YES

- Consult with parents.
- Provide parent education and home visits. In case of serious risk or non-response, involve other available resources and specialists.
- Consider legal interventions according to local legislation.
- Provide advice and offer services to those parents who need mental health services and consent to receive them.

4. Does the person have any other priority conditions?

(see mhGAP-IG Master Chart)

Look for the presence of:

- Developmental disorders
- Depression
- Alcohol or drug use
- Epilepsy
- Psychosis
- Suicidal behaviour

YES

If YES

Manage the concurrent condition according to the appropriate module for that disorder:

- Developmental Disorders » DEV
- Depression » DEP
- Alcohol Use Disorders » ALC
- Drug Use Disorders » DRU
- Epilepsy » EPI
- Psychosis » PSY
- Suicide / Self-harm » SUI

Behavioural Disorders

Intervention Details

Psychosocial Interventions

2.1 Family psychoeducation

- Accept and care for the child with a behavioural disorder.
- Be consistent about what the child is allowed and not allowed to do.
- Praise or reward the child after you observe good behaviour and respond only to most important problem behaviours; find ways to avoid severe confrontations or foreseeable difficult situations.
- Start behavioural change by focusing on a few very observable behaviours that you think the child can do.
- Give clear, simple and short commands that emphasize what the child should do rather than not do.
- Never physically or emotionally abuse the child. Make punishment mild and infrequent compared to praise. For example, withhold rewards (e.g. treats or fun activities) after a child does not behave properly.
- As a replacement for punishment, use short and clear-cut “time out” after the child shows problem behaviour. “Time out” is temporary separation from a rewarding environment, as part of a planned and recorded programme to modify behaviour. Brief the parents how to apply it when required.
- Put off discussions with the child until you are calm.

2.2 Advice to teachers

Make a plan on how to address the child’s special educational needs. Simple tips include:

- Ask the child to sit at the front of the class.
- Give the child extra time to understand assignments.
- Break long assignments into smaller pieces.
- Look for bullying and take appropriate action to stop it.

2.3 Support for carers

- Identify psychosocial impact on carers.
- Assess the carer’s needs and promote necessary support and resources for their family life, employment, social activities and health. Arrange for respite care, which means a break now and then when other trustable caregivers take over temporarily.

Intervention Details

Pharmacological interventions: Methylphenidate

» DO NOT use medication in primary care without consulting a specialist.

» DO NOT use medicines for general behavioural disorders in children and adolescents. Consider methylphenidate for hyperkinetic disorder only if psychosocial interventions have failed, child has been carefully assessed and is at least 6 years old, and conditions whose management can be complicated by methylphenidate have been ruled out.

» Use of stimulant medication must always be part of a comprehensive treatment plan that includes psychological, behavioural and educational interventions.

» Record the quantity dispensed and date of prescription, as well as response and side effects regularly in the medical record.

Methylphenidate

3.1 Initiating methylphenidate treatment

» Availability: Methylphenidate is restricted in many countries. Know the legislation governing the prescription and supply of stimulants.

» Preparation: Immediate-release formulations are usually available as a 10 mg tablet. Modified-release formulations are available in some countries.

» Pre-medication assessment for use of methylphenidate: In addition to the suggestions for assessment given in the flowchart, specifically assess:

– cardiovascular system – history, pulse, blood pressure, cardiac examination (obtain ECG if it is clinically indicated); methylphenidate is contraindicated in cardiovascular diseases;
– height and weight;
– risk assessment for substance use and drug diversion;
– specific medical diseases (e.g. use methylphenidate with caution in children with both ADHD and epilepsy);
– other mental disorders – methylphenidate may worsen anxiety and is contraindicated in psychosis.

» DO NOT prescribe without supervision of specialist.

3.2 Dosage and administration

» Immediate release formulation:
– Start at 5 mg once or twice daily.
– Gradually (over 4 – 6 weeks) increase the dose (in 2 – 3 divided doses) until there is no further improvement and side-effects are tolerable, up to a maximum recommended daily dose of 60 mg (in 2 – 3 divided doses).

» Extended-release preparations: If available, prescribe as a single dose in the morning.

3.3 Assessing response

Record symptoms and side-effects at each dose change. If rebound hyperactivity occurs when the effect of the drug wears off, the doses can be divided to include a late evening dose.

3.4 Side-effects

» Common side-effects: insomnia, decreased appetite, anxiety and mood changes
– reduce dose and refer to a pediatrician or psyschiatrist

» Less common side-effects: abdominal pain, headache, nausea, temporary growth retardation and low weight (monitor height and weight, consider a break in drug treatment over school holidays to allow 'catch-up' growth), change in blood pressure and heart rate (monitor before and after dose change and once in 3 months thereafter – consult a physician if sustained change over two recordings is seen), vomiting (give with food), tics.

» Routine blood tests and ECG are not required; they should be conducted if there is a clinical indication.

3.5 Follow-up

» Discontinue methylphenidate if there is no response after 1 month.

» Measure weight every 3 months and height every 6 months. If increase in height or weight stops, refer to a pedicatrician or Psychiatrist.

» If the child responds to methylphenidate, continue it for one year and then refer to a pediatrician or psychiatrist to check whether further continuation is justified.

Dementia

Dementia is a syndrome due to illness of the brain, which is usually chronic and progressive in nature. The conditions that cause dementia produce changes in a person's mental ability, personality and behaviour. People with dementia commonly experience problems with memory and the skills needed to carry out everyday activities. Dementia is not part of normal ageing. Although it can occur at any age, it's more common in older people.

People with dementia often present with complaints of forgetfulness or feeling depressed. Other common symptoms include deterioration in emotional control, social behaviour or motivation. People with dementia may be totally unaware of these changes and may not seek help. Sometimes it is thus the family who seeks care. Family members may notice memory problems, change in personality or behaviour, confusion, wandering or incontinence. However some people with dementia and their carers may deny or minimize the severity of memory loss and associated problems.

Dementia results in decline in intellectual functioning and usually interferes with activities of daily living, such as washing, dressing, eating, personal hygiene and toilet Activities.

1. Does the person have dementia?

- Ask the person, and also someone who knows them well, about problems with memory, orientation, speech and language, and difficulties in performing key roles and activities.
- Test orientation, memory and language. » **DEM 2**

- Ask the person or carer about the symptoms:
  - When were they first noticed?
  - What was the age of the person?
  - Was the onset sudden or gradual (over several months or years)?
  - What is the duration of the illness?
  - Are the symptoms worse at night ?
  - Is there associated drowsiness, impairment of consciousness?
  - Was the onset linked to a head injury, a blackout or a stroke?

- Is there a clinical history of:
  - Goitre, slow pulse, dry skin or hypothyroidism?
  - Sexually transmitted infection (STI) or HIV?
  - Cardiovascular disease?
  - Poor dietary intake, malnutrition, anaemia?

YES

If on testing, cognitive impairment or memory complaint:
- has been present for at least 6 months
- is progressive in nature, and
- is associated with impairment of social function

Dementia is likely

- Convey the result of the assessment. » **DEM 3.1**
- Offer psychosocial treatments for cognitive symptoms and functioning. » **DEM 3.2**
- Promote independence and maintain function. » **DEM 3.3**
- Follow up. » **DEM 3.6**

NO

- if the onset is abrupt and duration is short (days to weeks)
- If the disturbance is more at night and is associated with impairment of consciousness
- if there is disorientation to time / place

Delirium is likely

- Refer to a neurologist or Psychiatrist

NO

if there are any other unusual features, such as:
- Onset before the age of 60 years
- Clinical hypothyroidism
- Cardiovascular disease
- History of previous STI or HIV
- History of head injury or stroke

- Refer to a neurologist or Psychiatrist

Dementia

Assessment and Management Guide

2. Does the person have another Priority mental disorder?

» **Moderate to severe depression**
NOTE: Depression is common in dementia, but symptoms may be difficult to elicit from the person, so it may be necessary to rely on the carer's report of significant recent change.

» **psychosis**
(Assess according to mhGAP-IG Master Chart.)

YES — If **moderate to severe depression** is present

YES — If **psychosis** is present

» Manage the associated moderate-severe depression » **DEP** or psychosis. » **PSY**
» Review dementia diagnosis after successful treatment of depression. Cognitive impairment may be the result of severe depression, rather than dementia. Full recovery would indicate possibility of depression as the primary Problem.

3. Does the person have behavioural and Psychological symptoms of dementia?

Ask the carer about:

» Behaviour: wandering, night-time disturbance, agitation and aggression
» Psychological symptoms: hallucinations, delusions, anxiety or depressed mood
» When these symptoms occur and how often
» Whether these symptoms create a problem for the person or carer

Explore possible precipitants:

» Physical, such as pain, constipation, urinary infection
» Psychological, such as depression
» Environmental, such as crowding or any relocation

! look for imminent risk of harm to the person (e.g. from wandering) or carer (e.g. from assaultive behaviour that frightens the carer or has hurt others)

YES — If **behavioural and psychological symptoms** are present

» Manage with psychosocial therapies. » **DEM 3.4**
» Follow up. » **DEM 3.6**

YES — If **imminent risk of harm to person or carer**

» provide psychosocial therapies. » **DEM 3.4**
» In addition, offer pharmacological interventions. » **DEM 4**

4. Are cardiovascular disease and risk factors present?

Assess for
- Hypertension (blood pressure)
- Hyperlipidaemia
- Diabetes
- Smoking
- Obesity (weight, waist-to-hip ratio)
- Heart disease (angina or myocardial infarction)
- Previous stroke or transient ischaemic attacks

YES

If **cardiovascular risk factors or disease** are present

- Reduce cardiovascular risk factors according to local guidelines:
  - Advise person to stop smoking
  - Treat hypertension
  - Advise weight-reducing diet for obesity
  - Treat diabetes
- Refer to next level of care

5. Does the person suffer from other Physical conditions?

- Evaluate nutrition, eyesight, hearing, dentition, bladder and bowel function, and pain
- Obtain urinalysis
- Review medications, particularly those with significant anticholinergic side-effects (such as amitriptyline (an antidepressant); many antihistamines; antipsychotic drugs)

YES

if associated physical conditions are present, especially:
- Poor nutritional status
- Urinary tract infection
- Constipation or diarrhoea
- Medication interactions or Side-effects

- Treat associated physical conditions as it might improve cognition.
- Refer to next level of care

6. Is the carer experiencing strain or In need of support?

Assess
- Who is the main carer?
- Who else provides care and what care do they provide?
- Is there anything they find particularly difficult to manage?
- Are the carers coping? Are they experiencing strain? Are they depressed?
- Are they facing loss of income and / or additional expenses because of the needs for care?

YES

if carer is:
- Experiencing strain
- Depressed
- Facing overwhelming treatment costs

- Provide interventions for carers. » **DEM 3.5**
- Explore psychosocial interventions:
  - financial support such as disability services
  - information about the condition of the person
  - respite care
  - activation of community support network
  - family or individual therapy if available
- Follow up. » **DEM 3.6**
- Assess carer depression according to Depression Module and manage accordingly. » **DEP**

Dementia

Intervention Details

Identifying Dementia

Identifying dementia requires:

» Assessment of memory and cognitive functioning using simple tests

AND

» Confirmation by interviewing a family member or other person who knows the person well that these problems:

– Developed fairly recently
– Have been getting worse AND
– Are causing regular difficulties in carrying out daily work, domestic or social activities

Dementia can also be assessed using any locally validated tool.

2.1 Assess memory and cognitive functioning

» Assess memory by asking the person to repeat three common words immediately, then again 3 – 5 minutes later. (Shoe, yam, table)

» Assess orientation to time (time of day, day of week, season and year), and place (where is the person being tested, or where is the nearest market or store to their home).

» Test language skills by asking the person to name parts of the body, and explaining the function of an item, such as, “What do you do with a hammer?”

2.2 Interview key informant

» In an interview with a key informant (someone who knows the person well), ask about recent changes in thinking and reasoning, memory and orientation. Occasional memory lapses are common in older people, whereas some problems can be significant even if infrequent.

» Ask, for example, whether the person often forgets where they put things. Do they sometimes forget what happened the day before? Does the person sometimes forget where they are?

» Ask the informant when these problems started and whether they have been getting worse over time. Are there any time periods, lasting days, weeks or longer, when thinking and memory are completely back to normal?

» Identifying problems with daily activities can be difficult if the family minimizes these problems, if the problems are relatively minor, or if families routinely provide extensive support and care for older people. More complex tasks such as managing a house-hold budget, shopping or cooking tend to be affected first. Core self-care activities such as dressing, washing, feeding and toileting are only affected later.

» You will need to know the “usual activities” for older people in your area and for this particular family. Explore whether there have been any recent progressive changes. Does the person make errors or take longer to carry out tasks, perform them less well, or give up activities?

Intervention Details

Psychosocial Interventions

3.1 Conveying the results of the assessment

Ask people who are assessed for possible dementia whether they wish to know the diagnosis and with whom it should be shared. The explanation should be tailored to their ability to understand and retain information.

Start with giving basic information – but do not overload with too much information. Consider saying:

» Dementia is an illness of the brain and tends to get worse over time.

» Although there is no cure, there is much that can be done to help and support the person and the family. (Exercise control in declaring no cure is available).

» Many specific concerns and behaviours can be managed as they arise. A lot can be done to make the person more comfortable and to make life less stressful for the carer.

Make a realistic offer of ongoing help and support, and inform the person and carer of any other support available in their community.

3.2 Psychosocial interventions for cognitive symptoms and functioning

» Provide regular orientation information (e.g. day, date, weather, time and names of people) to people with dementia to help them to remain oriented to time, place and person.

» Use materials such as newspapers, radio or TV programmes, family albums and household items to promote communication, to orient them to current events, to stimulate memories and to enable people to share and value their experiences.

» Use simple short sentences to make verbal communication clear. Try to minimize competing noises, such as the radio, TV, or other people's conversation. Listen carefully to what the person has to say.

» Keep things simple, avoid changes to routine and avoid exposing the person to unfamiliar and bewildering places unless this is necessary.

3.3 Promote independence, functioning and mobility

Plan for activities of daily living in a way that maximizes independent activity, enhances function, helps to adapt and developskills, and minimizes need for support. Facilitate rehabilitation in the community involving people and their carers in planning and implementation of these interventions. Assist in liaison with available social resources.

Specifically consider the following:

» Give advice to maintain independent toileting skills, including prompting and regulation of fluid intake (if incontinence occurs, all possible causes should be assessed and treatment options tried before concluding it is permanent).

» Inform family members that it is important to keep the floor of the person's home without clutter as to reduce the risk of falling.

» Recommend making adaptations in the person's home. It can be helpful to add hand-rails or ramps. Signs for key locations (e.g. toilet, bath, bedroom) can help to ensure that the person does not get lost or lose orientation while at home.

» Recommend physical activity and exercise to maintain mobility and to reduce the risk of falls.

» Advise recreational activities (tailored to the stage of dementia).

» Refer to occupational therapy, if available.

» Manage sensory deficits (such as low vision, poor hearing) with appropriate devices (e.g. magnifying glass, hearing aids).

Dementia

DEM 3

Intervention Details

Psychosocial Interventions

3.4 Managing behavioural and Psychological symptoms

» Identify and treat underlying physical health problems that may affect behaviour (pain, infections, etc).

» Consider environmental modifications, such as appropriate seating, safe wandering areas, signs (e.g. no exit sign on street door, or signpost to toilet).

» Identify events (e.g. shopping at a busy market) or factors (e.g. going out alone) which may precede, trigger or enhance problem behaviours, and try to see if that can be modified.

» Consider soothing, calming or distracting strategies, such as suggesting the person does activities they enjoy (e.g. Going for a walk, listening to music, engaging in conversation), especially when they are feeling agitated.

As people with dementia are at risk of abuse and neglect, health-care providers should be vigilant to protect the person with dementia, and they should apply any relevant local policies in this regard.

3.5 Intervention for carers

» Identify psychological distress and psychosocial impact on carers. Assess the carer's needs to ensure necessary support and resources for their family life, employment, social activities and health.

» Acknowledge that it can be extremely frustrating and stressful to care for people with dementia. It is important that carers continue to take care of people with dementia, avoiding hostility towards, or neglect of, the person. Carers need to be encouraged to respect the dignity of the person with dementia, involving them in decisions on their life as far as possible.

» Provide information to people with dementia, as well as family and other informal carers, from the time of diagnosis. This must be done sensitively and bearing in mind the wishes of the person and carers.

» Provide training and support in specific skills (like managing difficult behaviour) if necessary. To make these interventions most effective, elicit the active participation of the carer (for example through role-play).

» Consider providing practical support, e.g. where feasible, home-based respite care. Another family member or suitable person can supervise and care for the person with dementia (preferably in the usual home setting). This can relieve the main caregiver who can then rest or carry out other activities.

» Explore whether the person qualifies for any disability benefits or other social or financial support. This may be from government or non-governmental source or social networks.

» If feasible, try to address the carer's psychological strain with support, problem-solving counselling, or cognitive-behaviour interventions (see Depression and Advanced Psychosocial Interventions Module). **» DEP** and **» INT**

» Assess for and treat moderate-severe depression in carers (see Depression Module). **» DEP**

3.6 Follow-up

» At least once every three months, perform a regular medical and social care review.

» at each follow-up, assess:
- medical and psychiatric co-morbidities, including visual and hearing impairment, pain, continence;
- stability or progression of symptoms of dementia, looking for any new symptoms and any rapid changes;
- ability to participate in activities of daily living, and any new needs for care;
- safety risks (e.g. driving, financial management, medication management, home safety risks from cooking or smoking, possibility of wandering, etc.);
- presence and severity of depression;
- presence and severity of behavioural and psychological symptoms of dementia;
- risk of self-harm;
- if on any medication: treatment response, side-effects and adherence as well as cognitive and target symptoms;
- any barriers to participating in treatment;
- carer strain and coping, needs for information, training and support.

» Compare current assessment with previous notes, and discuss findings with the person and their carer.

» Determine any new supportive needs of the person and / or their carer and facilitate as necessary.

» Discuss and agree on any changes in the treatment plan.

Intervention Details

Pharmacological interventions

» For behavioural and psychological symptoms of dementia, consider antipsychotic medication such as haloperidol or an atypical antipsychotic medications only after a trial of psychosocial interventions. You may consider these medications if symptoms persist and there is imminent risk of harm.

» CONSULT A PSYCHIATRISTS OR NEUROLOGIST

Alcohol Use and Alcohol Use Disorders

ALC

Conditions resulting from different patterns of alcohol consumption include acute alcohol intoxication, harmful alcohol use, the alcohol dependence syndrome, and the alcohol withdrawal state. Acute intoxication is a transient condition following intake of alcohol resulting in disturbances of consciousness, cognition, perception, affect or behaviour. Harmful use of alcohol is a pattern of alcohol consumption that is causing damage to health. The damage may be physical (e.g. liver disease) or mental (e.g. episodes of depressive disorder). It is often associated with social consequences (e.g. Family problems, or problems at work).

Alcohol dependence is a cluster of physiological, behavioural and cognitive phenomena in which the use of alcohol takes on a much higher priority for a given individual than other behaviours that once had greater value. The alcohol withdrawal state refers to a group of symptoms that may occur upon cessation of alcohol after its prolonged daily use.

Alcohol Use and Alcohol Use Disorders

Assessment and Management Guide >> for Emergency Cases

1. Is the person acutely intoxicated With alcohol?

Look for:
- Smell of alcohol on the breath
- Slurred speech
- Uninhibited behaviour

Assess:
- Level of consciousness
- Cognition and perception

YES

If there is disturbance in the level of consciousness, cognition, perception, affect or behaviour following recent consumption of alcohol

Alcohol Intoxication is likely

- Assess airway and breathing.
- Put the person on their side to prevent aspiration in case they vomit.
- Refer to hospital if necessary or observe until effects of alcohol have worn off.
- **If methanol poisoning is suspected, refer to hospital for emergency management.**

2. Does the person have features of Alcohol withdrawal?

Alcohol withdrawal occurs following cessation of heavy alcohol consumption, typically between 6 hours and 6 days after the last drink.

Look for:
- Tremor in hands
- Sweating
- Vomiting
- Increased pulse and blood pressure
- Agitation

Ask about:
- Headache
- Nausea
- Anxiety

NOTE: Seizures and confusion may occur in severe cases.

YES

Is withdrawal likely to be SEVERE? Look for:
- Past episodes of severe alcohol withdrawal including delirium and seizures
- Other medical or psychiatric problems or benzodiazepine dependence
- Severe withdrawal symptoms already present only a few hours after stopping drinking

- Treat **immediately** with diazepam. » **ALC 3.1**
- Treat in hospital or detoxifi cation centre if available.
- **If withdrawal is complicated by delirium:** » **ALC 3.1**
  - Treat the withdrawal with diazepam.
  - Manage in a safe environment.
  - Keep well hydrated.
  - If delirium or hallucinations persist despite treatment of other withdrawal symptoms, then consider using antipsychotics such as haloperidol 2.5 – 5 mg orally up to 3 times daily.
- **If withdrawal is complicated by a seizure,** treat with diazepam in the first instance and do not use anticonvulsants to prevent further seizures.

Alcohol Use and Alcohol Use Disorders

ALC 1

Assessment and Management Guide >> for Emergency Cases

3. Does the person have acute confusion or clouding of consciousness with recent history of heavy alcohol consumption?

YES

Is this acute Wernicke's encephalopathy, head injury or alcohol-withdrawal delirium?

- Examine for nystagmus and ataxia of Wernicke's encephalopathy. Ophthalmoplegia may occur in severe cases.
- Examine for signs of head injury such as lacerations, or bleeding around head or ears.
- Re-assess for alcohol withdrawal Delirium

Exclude other common causes of confusion, such as infections, hypoxia, hypoglycaemia, hepatic encephalopathy, and cerebrovascular accidents.

Acute Wernicke's encephalopathy

- Treat all suspected cases with i.v. or i.m. Thiamine 100 mg 3 times daily for 3 – 5 days.
- Refer the person urgently to the hospital.

Head injury

- Monitor level of consciousness.
- Seek surgical opinion.

Alcohol withdrawal delirium

- Treat alcohol withdrawal delirium. » ALC 3.1

Alcohol Use and Alcohol Use Disorders

Assessment and Management Guide

1. Look for a pattern of alcohol Consumption and alcohol-related harm

» **Ask if the person consumes alcohol.**

If YES

» **Ask if the person consumes alcohol in a way that puts them at risk of harm:**

Drinking quantity and frequency

– Has consumed 5 or more standard drinks (or 60 g alcohol)* on any given occasion in the last 12 months
– Drinks on average more than two drinks per day
– Drinks every day of the week

* *A standard drink is a measure of the amount of pure alcohol consumed, usually between 8 g and 12 g. If the amount of alcohol contained in a standard drink in that country is outside these limits, the number of standard drinks may need to be adjusted.*

NOTE: Detecting alcohol use disorders in routine care

Hazardous use of alcohol and alcohol use disorders are common. Except in areas of very low alcohol consumption, people presenting to the health facility should be asked about their alcohol consumption. This may be done informally or by the use of a questionnaire such as the WHO-AUDIT, or the WHO–ASSIST.

YES

If YES

» Conduct a more detailed history to look for harmful alcohol use or dependence **(see point 2 below and » ALC 2.1)**

If no alcohol dependence

» **the clinical scenario is**
HAZARDOUS USE OF ALCOHOL
or
HARMFUL USE OF ALCOHOL

» State clearly the results of alcohol use assessment
and explain the links between this level of alcohol use, the person's health problems, and the short-term and long-term risks of continuing use at the current level.
» Ask about other substance use **» DRU 2.1**
» Have a short discussion about the person's reasons for their alcohol use.

See Brief Interventions » ALC 2.2 for details

» State clearly the recommendation to either cut down to safer levels or stop using alcohol completely and your willingness to help the person to do so.
– If the person is willing to try to cut down or stop using alcohol, then discuss ways of achieving this objective.
– If not, communicate confidently that it is possible to stop or reduce hazardous or harmful alcohol use and encourage the person to come back if he or she wants to discuss the issue further.

» Follow up at the next opportunity.
» Seek specialist advice as needed for people with ongoing harmful use of alcohol who have failed to respond to brief interventions.

If the person is an adolescent, see **» ALC 2.6**

If it is a women who is pregnant or breastfeeding,

Alcohol Use and Alcohol Use Disorders

ALC 1

Assessment and Management Guide >> for Emergency Cases

2. Does the person have alcohol Dependence?

Conduct a detailed alcohol use history » ALC 2.1

Look for:

- A strong desire or sense of compulsion to take alcohol
- Difficulties in controlling alcohol use in terms of its onset, termination or levels of use
- A physiological withdrawal state when alcohol use has ceased or been reduced, as shown by the characteristic withdrawal syndrome for alcohol; or use of the same (or a closely related) substance with the intention of relieving or avoiding withdrawal symptoms
- Evidence of tolerance, such that increased doses of alcohol are required in order to achieve effects originally produced by lower doses
- Progressive neglect of alternative pleasures or interests because of alcohol use, increased amount of time necessary to obtain or take alcohol or to recover from its effects
- Alcohol use persisting despite clear evidence of overtly harmful consequences, such as harm to the liver, depressive mood states, or impairment of cognitive functioning

YES

If 3 or more features are present:

the clinical scenario is ALCOHOL DEPENDENCE

- State clearly the results of the assessment, and explain both the short-term and long-term risks of continuing use at the current level.
- Have a short discussion about the person's motivations for their alcohol use. **See Brief Interventions. » ALC 2.2**
- Advise complete cessation of alcohol.
- Advise daily consumption of thiamine 100 mg.
- If the person is willing to try to stop using alcohol, facilitate alcohol cessation.
  - Determine the appropriate setting to cease alcohol.
  - Plan the cessation of alcohol.
  - Arrange detoxification if necessary.
  - During detoxification, treat withdrawal symptoms with diazepam. **» ALC 3.1**
- After detoxification, prevent relapse with medication (naltrexone, acamprosate or disIfiran), if available. **» ALC 3.2**
- Assess and treat any medical or psychiatric co-morbidity, ideally after 2 – 3 weeks of abstinence as some problems will resolve with abstinence.
- Consider referral to a self-help group (such as Alcoholics Anonymous), or a residential therapeutic community **» ALC 2.3**
- DO NOT administer punishment in the name of treatment.
- Address housing and employment needs. » ALC 2.4
- Provide information and support to person, carers and family members. » ALC 2.5
- If available, provide psychosocial interventions such as family counselling or therapy, problem-solving counselling or therapy, cognitive behavioural therapy, motivational enhancement therapy, or contingency management therapy. » INT
- Consider referral to a specialized treatment facility.
- Follow up as needed, frequently initially.
- Seek specialist support as needed.

Intervention Details

2.1 Taking alcohol use history

When asking about alcohol consumption:

» Ask questions without indicating a preferred answer, and try not to display surprise at any responses given.

» Ask about the level and pattern of consumption of alcohol, as well as any behaviours associated with alcohol use that may risk the person's health and the health of others (i.e., where, when and with whom alcohol consumption typically occurs, what triggers alcohol consumption, activities when intoxicated, financial implications, capacity to care for children, and violence towards others).

» Ask about harms from alcohol, including:
- accidents, driving while intoxicated
- relationship problems
- medical problems such as liver disease / stomach ulcers
- legal / financial problems
- sex while intoxicated and that is later regretted or risky
- alcohol-related violence including domestic violence

» Ask about commencement and development of alcohol use in relation to other life events, for example, by taking a chronological history.

» If there is evidence of hazardous or harmful alcohol use, investigate dependence by asking about the development of tolerance, withdrawal symptoms, use in greater amounts or over a greater length of time than was intended, continued alcohol use in the face of problems related to it, difficulty in stopping or cutting down alcohol use, and craving for alcohol use.

» Ask about social networks and the person's alcohol and other drug consumption patterns.

When examining the person, look for:

» presence of intoxication and withdrawal;

» evidence of long-term heavy alcohol consumption, such as liver disease (swollen or shrinking liver, peripheral signs of liver injury), cerebellar or peripheral nerve damage.

Investigations that should be considered (when possible):

» liver enzymes and full blood examination.

» Abdomino - Pelvic Ultrasound.

2.2 Brief interventions to reduce harmful alcohol consumption

» **Examples of ways that the harmful or hazardous use of alcohol can be reduced**
- not having alcohol at home;
- not going to pubs or other locations where people use alcohol;
- asking support from family or friends;
- asking the person to come back with family or friends and to discuss a way forward together at the health centre.

» **Talking to people about the reasons they use alcohol**
- Engage the person in a discussion about their alcohol use in a way that he / she is able to talk about both the perceived benefits of it and the actual and / or potential harms, taking into consideration the things that are most important to that person in life.
- Steer the discussion towards a balanced evaluation of the positive and negative effects of alcohol by challenging overstated claims of benefits and bring up some of the negative aspects which are perhaps being understated.
- Avoid arguing with the person and try to phrase something in a different way if it meets resistance – seeking to find understanding of the real impact of alcohol in the person's life as much as is possible for the person at that time.
- Encourage the person to decide for themselves if they want to change their pattern of alcohol use, particularly after there has been a balanced discussion of the pros and cons of the current pattern of use.
- If the person is still not ready to stop or reduce alcohol use, then ask the person to come back to discuss further.

2.3 Self-help groups

» Consider advising people with alcohol dependence to join a self-help group, e.g. Alcoholics Anonymous. Consider facilitating initial contact, for example by making the appointment and accompanying the person to the first session.

2.4 Address housing and employment needs

» Where available, work with local agencies and community resources to provide supported employment for those who need support to return to work or find a job and to enable access to local employment (or educational) opportunities, based on the person's needs and skill level.

Alcohol Use and Alcohol Use Disorders

Intervention Details

» Where available, work with local agencies and community resources to find supported housing or assisted living facilities, as well as independent living facilities, if these are needed. Carefully consider the capacity of the person and the availability of alcohol or other substances in advising and facilitating optimal housing arrangements.

2.5 Supporting families and carers

Discuss with families and carers the impact of alcohol use and dependence on themselves and other family members, including children. Based on feedback from families:

» Offer an assessment of their personal, social and mental health needs.

» Provide information and education about alcohol use and dependence.

» Help to identify sources of stress related to alcohol use; explore methods of coping and promote effective coping behaviours.

» Inform them about and help them access support groups (e.g. self-help groups for families and carers) and other social resources.

2.6 Substance use in adolescence

» Clarify the confidential nature of the health care discussion, including in what circumstances parents or other adults will be given information.

» Identify the most important underlying issues for the adolescent, keeping in mind that adolescents are often not able to articulate their problems well. This might mean asking open ended questions covering the areas covered by the HEADS acronym (Home, Education / Employment / Eating, Activities, Drugs and alcohol, Sexuality / Safety / Suicide) and allowing sufficient time for the discussion.

» Although they usually present with less severe substance abuse problems, young people can present with severe dependence. It is just as important to screen adolescents for drug and alcohol problems as adults.

» Provide parents and the adolescent with information on the effects of alcohol and other substances on individual health and social functioning.

» Encourage a change in the adolescent's environment rather than focusing directly on the adolescent as being the problem, such as by encouraging participation in school or work and activities after school / work that occupy the adolescent's time, and encourage participation in group activities which facilitate the adolescent's skill acquisition and contribution to their communities. It is important that adolescents are involved in activities which interest them.

» Encourage parents and / or responsible adults to know where the adolescent is, who they are with, what they are doing, when they will be home, and to expect the adolescent to be accountable for their activities.

» Encourage parents to set clear expectations (at the same time being prepared to negotiate these expectations with the adolescent), and to discuss with adolescents the consequences of the adolescent's behaviours and non conformity with expectations.

» Advise parents to limit their own behaviours which may be contributing to their children's substance use, including the purchasing or providing of alcohol or the provision of funds which are being spent on substance use, keeping in mind the potential influence of their own alcohol and drug use on their children.

2.7 Women – Pregnancy and breastfeeding

» Advise women who are pregnant or considering becoming pregnant to avoid alcohol completely.

» Advise women that consuming even small amounts of alcohol early in pregnancy can harm the developing foetus, and that larger amounts of alcohol can result in a syndrome of severe developmental problems called Foetal Alcohol Syndrome (FAS).

» Advise women who are breastfeeding to avoid alcohol completely.

» Given the benefits of exclusive breastfeeding (particularly in the first 6 months), if mothers continue to drink alcohol they should be advised to limit their alcohol consumption, and to minimise the alcohol content of the breast milk, such as by breastfeeding before drinking alcohol and not again until after blood levels fall to zero (allowing approximately 2 hours for each drink consumed, i.e. 4 hours if TWO drinks are consumed), or using expressed breast milk.

» Mothers with harmful substance use and young children should be offered what social support services are available, including additional post natal visits, parenting training, and child care during medical visits.

Intervention Details

3.1 Management of alcohol withdrawal

- Be alert for the person at risk of a withdrawal syndrome, for example, the person with undiagnosed alcohol dependence in the district hospital.
- When there is evidence of a withdrawal syndrome developing (or before withdrawal symptoms develop in the case of planned withdrawal), administer diazepam at an initial dose of up to 40 mg daily (i.e., 10 mg four times daily or 20 mg twice daily) for 3 – 7 days. In people with impaired hepatic metabolism (e.g. liver failure, elderly) use a single low dose initially (5 – 10 mg) and determine the duration of action of this dose before prescribing further doses.

> **!**
> *The dose and duration of diazepam treatment should be determined individually, according to the severity of withdrawal and the presence of other medical disorders. In the hospital setting, diazepam can be given more frequently (i.e., hourly) and up to higher daily doses (i.e., up to 120 mg daily for the fi rst 3 days) if necessary and based on frequent assessment of the Person.*

- Administer thiamine 100 mg / day orally for 5 days (or longer if required) to prevent the development of thiamine-deficiency syndromes such as Wernicke's encephalopathy. Consider other vitamin supplementation when indicated.
- Ensure adequate fluid intake and electrolyte requirements are met. Correct potassium and magnesium levels that are typically low.
- Ensure carer support.
- Provide as quiet and non-stimulating an environment as possible, which is well lit in the day time and lit enough at night to prevent falls if the person gets up in the night.
- When the person has severe alcohol dependence (previous history of severe alcohol withdrawal, seizures or delirium) or concurrent serious medical or psychiatric disorders or is lacking adequate support (REFER TO A DOCTOR OR NEXT LEVEL OF CARE)
- If other medical problems are suspected, consider referral to a doctor for assessment and treatment

WHERE to withdraw from alcohol?

- Have there been past episodes of severe withdrawal symptoms, seizures or delirium?
- Are there other significant medical or psychiatric problems?
- Do significant withdrawal features develop within 6 hours of the last drink?
- Has outpatient withdrawal failed?
- Is the person homeless or without any social support?

If YES to any of the above then inpatient withdrawal is preferable.

Alcohol-withdrawal delirium

- Treat the person in a low stimulus and safe environment where they are unlikely to do themselves harm.
- Treat underlying alcohol withdrawal with diazepam.
- Administer thiamine 100 mg i.v. or i.m. 3 times daily for 5 days.
- Use antipsychotic medication, if necessary, for the duration of psychotic symptoms only (e.g. Haloperidol 2.5 – 5 mg orally tds).
- Maintain hydration.
- Avoid restraining the person by physical force like tying with a rope or wire.

Always consider other causes of delirium and hallucinations (e.g. head injury, hypoglycaemia, infection (most commonly pneumonia), hypoxia, hepatic encephalopathy or cerebrovascular accidents).

Alcohol Use and Alcohol Use Disorders

Intervention Details

3.2 Relapse-prevention medications after withdrawal from alcohol

Several medications are useful in the treatment of alcohol dependence and increase the likelihood of the person maintaining abstinence from alcohol. The principal medications are acamprosate, naltrexone and disulfiram. The decision to use of any of these medications should be made taking into consideration preferences of the person and an assessment of benefit versus risk (e.g. is there an excessive risk if the medication is administered by non-medically trained health workers or if the person has liver disease or is using other drugs). All three medications should be avoided, if possible, in women who are pregnant or breastfeeding, and in people with significant renal or hepatic impairment, although each situation should be individually assessed. Where a specialist centre is available, the person can be referred there for these and other treatments. With these medications, an effective response may include a reduction in the quantity and frequency of alcohol consumption, if not complete abstinence.

3.2.1 Acamprosate

Acamprosate suppresses the urge to drink alcohol in the alcohol-dependent person. It is best started immediately after withdrawal from alcohol has been achieved. It is given at a dose of 2 tablets (each containing 333 mg acamprosate) three times per day, except in people with a body weight of less than 60 kg, when the dose is reduced to 2 tablets twice daily. Treatment is usually administered for 12 months. Adverse reactions associated with acamprosate treatment occur in about 20% of patients and include diarrhoea, nausea, vomiting, abdominal pain, pruritus, occasionally maculopapular rash, and, rarely, bullous skin reactions.

3.2.2 Naltrexone

Naltrexone also suppresses the urge to drink alcohol. It can be started after withdrawal from alcohol at a dose of 50 mg daily. It is then maintained within the 50 – 100 mg range for 12 months. Importantly, the person must not have taken any opioid drugs for the previous 7 days. The person must be warned that naltrexone will block opioid drugs in case they need opioid analgesia in the near future. Adverse reactions occur in about 20% of patients and include nausea, vomiting, abdominal pain, anxiety, sleeping difficulties, headache, reduced energy, joint and muscle pain. Liver toxicity can occur with higher doses of naltrexone and, if feasible, liver function tests should be routinely carried out.

3.2.3 Disulfiram

The effect of disulfiram is based on the fear of the unpleasant and potentially dangerous reaction, which includes facial flushing, nausea, vomiting and fainting, when alcohol is consumed by the person taking the medication. The person must be advised of its mechanism of action and the nature of the disulfiram-alcohol reaction, including the fact that 1 in 15,000 patients treated with it die as a result of the reaction (this is low compared with the risk of dying from untreated alcohol dependence). Disulfiram should be offered to motivated patients for whom medication adherence can be monitored by treatment personnel, carers or family members, and when health-care providers are aware of potential adverse effects, including the disulfiram-alcohol reaction. The dose is typically 200 mg daily. Adverse reactions include drowsiness, fatigue, nausea, vomiting, reduced libido, rarely psychotic reactions, allergic dermatitis, peripheral neuritis or hepatic cell damage. Disulfiram is contraindicated in people with coronary heart disease, cardiac failure, history of cerebrovascular accidents, hypertension, psychosis, severe personality disorders or suicide risk.

Drug Use and Drug Use Disorders

Conditions resulting from different patterns of drug use include acute sedative overdose, acute stimulant intoxication or overdose, harmful or hazardous drug use, cannabis dependence, opioid dependence, stimulant dependence, benzodiazepine dependence, and their corresponding withdrawal states.

Harmful use of drugs is a pattern of drug consumption that is causing damage to health. The damage may be physical (as in cases of infections related to drug use) or mental (e.g. episodes of depressive disorder) and is often associated with damage to social functioning (e.g. family problems, legal problems or work-related problems).

Drug dependence is a cluster of physiological, behavioural and cognitive phenomena in which drug use takes on a much higher priority for a given individual than other behaviours that once had greater value.

The drug withdrawal state refers to group of symptoms occurring upon cessation of a drug after its prolonged daily use.

Drug Use and Drug Use Disorders

Assessment and Management Guide >> for Emergency Cases

1. Is the person suffering from A sedative overdose?

Opioid overdose or other sedative overdose or mixed drug with or without alcohol overdose
- Unresponsive or minimally responsive
- Slow respiratory rate
- Pinpoint pupils (opioid overdose)

YES

If
- Respiratory rate < 10 **OR**
- Oxygen saturation < 92 %

- **Treat airway, breathing and circulation**
- **Naloxone 0.4 mg subcutaneous, i.m. or i.v. (for opioid overdose – but ineffective for other sedative overdose), repeat if necessary.**
- Observe for 1 – 2 hours after naloxone administration.
- For overdoses due to long acting opioids – transport to hospital for naloxone infusion or ventilatory support.

If unresponsive to naloxone
- provide airway and ventilatory support and transport to hospital

2. Is the person in a state of acute Stimulant intoxication or overdose?

- Dilated pupils
- Excited, racing thoughts, disordered thinking, paranoia
- Recent use of cocaine or other stimulants
- Raised pulse and blood pressure
- Aggressive, erratic, or violent behaviour

YES

Cocaine or amphetamine-type stimulant intoxication or overdose

- Give diazepam in titrated doses until the person is calm and lightly sedated.
- If psychotic symptoms do not respond to benzodiazepines, then consider using short-term antipsychotics.
- DO NOT commence long-term antipsychotics.
- Monitor blood pressure, pulse rate, respiratory rate, temperature every 2 – 4 hours.
- If the person complains of chest pain, if tachyarrhythmias are present or if the person becomes violent or unmanageable, transfer the person to hospital.
- During the post-intoxication phase – be alert for suicidal thoughts or actions.

3. Is the person suffering from Acute opioid withdrawal?

- History of opioid dependence, recent heavy use ceasing in the last days
- Muscle aches and pains, abdominal cramps, headaches
- Nausea, vomiting, diarrhoea
- Dilated pupils
- Raised pulse and blood pressure
- Yawning, runny eyes and nose, pilo-erection ("goosefl esh")
- Anxiety, restlessness

YES

Opioid withdrawal

- Treat with reducing doses of opioids or alpha-adrenergic agents (clonidine) using either supervised dosing or daily dispensing.
- Treat specifi c symptoms as needed (diarrhoea, vomiting, muscle pain, insomnia).
- Consider starting opioid agonist maintenance treatment.
- Oral or i.v. rehydration, if necessary.

Drug Use and Drug Use Disorders

Assessment and Management Guide

1. Does the person use illicit or nonprescribed drugs in a way that risks damage to health?

- Ask about recent drug use. **» DRU 2.1**
- Look for drug-related harm.

NOTE: *Screening questionnaires, such as the WHO–ASSIST, can be used to screen for drug use and related problems*

YES

If YES then assess for dependence (see no. 2 below) and drug related harm. If the person is NOT drug dependent, then the clinical scenario is:

HAZARDOUS DRUG USE
or
HARMFUL DRUG USE

- State clearly the results of substance use assessment and explain the links between this level of substance use, the person's health problems, and the short-term and long-term risks of continuing use at the current level.
- Ask about alcohol and other substance use. **» ALC 2.1**
- Have a short discussion about the person's reasons for their drug use.
- **See Brief Interventions » DRU 2.2** for details
- State clearly the recommendation to stop harmful substance use and your willingness to help the person to do so.
  - If the person is willing to try to cut down or stop drug use, then discuss ways of achieving this objective.
  - If not, communicate confidently that it is possible to stop or reduce hazardous or harmful substance use and encourage the person to come back if he or she wants to discuss the issue further.
- If the person is an adolescent, see the section on adolescent substance use. **» ALC 2.6**
- If it is a women who is pregnant or breastfeeding , see **» DRU 2.7**
- Follow up at the next opportunity.
- Seek specialist advice as needed for people with ongoing harmful drug use who have failed to respond to brief Interventions.

NOTE: Case Finding
The prevalence of drug use disorders varies enormously between settings. In settings of high levels of drug use, it might be appropriate to ask all patients about their drug consumption. This may be done informally or by the use of a questionnaire such as the WHO-ASSIST. In other settings, ask about drug use when clinical features suggest the presence of a drug use disorder. These include unkempt appearance, injuries, infections and mental health problems.

2. Are there features of drug dependence?

» **Conduct a detailed drug use assessment. » DRU 2.1**

Look for:

» Strong desire or sense of compulsion to take drugs

» Difficulties in controlling drug use in terms of its onset, termination or levels of use

» A physiological withdrawal state when drug use has ceased or been reduced, as shown by the characteristic drug-withdrawal syndrome; or use of the same (or a closely related) substance with the intention of relieving or avoiding withdrawal symptoms

» Evidence of tolerance, such that increased doses of the substance are required in order to achieve effects originally produced by lower doses

» Progressive neglect of alternative pleasures or interests because of drug use, increased amount of time necessary to obtain or take drug or to recover from its effects

» Drug use persisting, despite clear evidence of overtly harmful consequences

YES

If 3 or more features are present,

the clinical scenario is:
DRUG DEPENDENCE

» State clearly the diagnosis and inform about the risks of short-term and long-term harm.

» Explore the person's reasons for their drug use, using brief intervention techniques. **» DRU 2.2**

» Advise the person to stop using the substance completely and indicate the intention to support the person in doing so.

» Ask the person if they are ready to try to stop using the substance.

» **Cannabis or Stimulant Dependence**
- Provide a more intensive brief intervention (i.e., up to 3 sessions, each lasting up to 45 minutes). **» DRU 2.2**
- Treat withdrawal symptoms. **» DRU 3.3**
- Arrange detoxification service if necessary.

» **Opioid dependence**
- Assess severity of dependence. **» DRU 2.1**
- In most cases, advise opioid agonist maintenance treatment (also known as opioid-substitution Therapy, OST). Provide or refer for OST, if available. **» DRU 3.1**
- Arrange planned detoxification if requested. **» DRU 3.1**

» **Benzodiazepine dependence**
- Gradual reduction of benzodiazepines with supervised dispensing or more rapid reduction of benzodiazepines in an in-patient setting. **» DRU 3.2**

IN ALL CASES

» Consider referral to self-help groups, and rehabilitation / therapeutic communities. **» DRU 2.3**

» Address housing and employment needs. **» DRU 2.4**

» Provide information and support to person, carers and family members. **» DRU 2.5**

» If available, provide psychosocial interventions, such as family counselling or therapy, problem-solving counselling or therapy, cognitive behavioural therapy, motivational enhancement therapy, contingency management therapy. **» INT**

» Offer harm reduction strategies for people who inject drugs. **» DRU 2.6**

Drug Use and Drug Use Disorders

Intervention Details

2.1 Assessment

Taking a drug use history

When asking about drug consumption:

» Ask questions about illicit drug use in a non-judgemental way, perhaps after asking about tobacco / nicotine, alcohol and any traditional drug use that may be relevant.

» Ask about the level and pattern of consumption, and any behaviours associated with drug use that may risk health and the health of others (e.g. drug smoking, drug injection, activities when intoxicated, financial implications, capacity to care for children, violence toward others).

» Ask about commencement and development of drug use in relation to other life events, for example by taking a chronological history.

» Ask about harms from drug use including:
- injuries and accidents
- driving while drug affected
- relationship problems
- drug injection and related risks
- legal / financial problems
- sex while intoxicated and that is risky or later regretted

» Enquire about dependence by asking about the development of tolerance, withdrawal symptoms, use in greater amounts or over a greater length of time than was intended, continued drug use in the face of problems related to drug use, difficulty in stopping or cutting down drug use, and craving for drug use.

Things to look for in the examination

» **Signs of injection:** Common injection sites are the antecubital fossae or groin. The person may demonstrate where they have injected drugs. Old injection marks appear as skin pigmentation, or thinning of the skin. New injection sites are small and usually slightly red and inflamed. In cases of dependence on injected drugs (such as heroin), both new and old injection sites should be visible.

» **Presence of intoxication and withdrawal:**
- Opioid intoxication: drowsiness, being “on the nod”, slow speech, small pupils and depressed respiration.
- Opioid withdrawal: anxiety, dilated pupils, abdominal cramps, yawning, runny nose, and piloerection (“gooseflesh”).

- Benzodiazepines intoxication: sedation, slowed and slurred speech, depressed respiration.
- Benzodiazepine withdrawal: anxiety and agitation, muscle cramps, abdominal cramps, raised pulse and blood pressure, insomnia, and (when severe) seizures and delirium.

- Stimulant intoxication: hyperactivity, rapid speech and dilated pupils.
- Stimulant withdrawal: initially fatigue, increased appetite, irritability, emotional depression, and anxiety.

- Cannabis intoxication: red conjunctivae, delayed responsiveness, and normal pupil size.
- Cannabis withdrawal: mood lability, anxiety and muscle cramps (there may not be any observable features).

» **Physical appearance and mental state.** Physical appearance is a useful guide for the capacity to self-care.

» **Common health complications of injecting drug use:** There may be evidence of HIV infection and related illnesses, hepatitis B or C, injection site infections, or tuberculosis.

» **Other common health conditions associated with drug use:** poor dentition, parasitic skin infections (lice, scabies), sexually transmitted diseases, malnutrition.

Investigations which should be considered

» Urine drug screen: It can be a useful way of verifying a drug use history, particularly if the person has something to gain by not telling the truth. It should be conducted before commencing OST, and the results should be available before the third dose (to prevent accidental overdose in people not opioid dependent starting OST). A urine dipstick can be used, although it is less reliable.

» Serology for blood-borne viruses (HIV, hepatitis B and C).

» Tests for sexually transmitted infections (STI).

» Sputum testing (when tuberculosis is suspected).

Intervention Details

Psychosocial Interventions

2.2 Brief intervention techniques

Ways to discuss substance use:

- Engage the person in a discussion about their substance use in a way that he / she is able to talk about both the perceived benefits of it and the actual and / or potential harms, taking into consideration the things that are most important to that person in life.
- Steer the discussion towards a balanced evaluation of the positive and negative effects of the substance by challenging overstated claims of benefits and bring up some of the negative aspects which are perhaps being understated.
- Avoid arguing with the person and try to phrase something in a different way if it meets resistance – seeking to find understanding of the real impact of the substance in the person's life as much as is possible for that person at that time.
- Encourage the person to decide for themselves if they want to change their pattern of substance use, particularly after a balanced discussion of the pros and cons of the current pattern of use.
- If the person is still not ready to stop or reduce substance use, then ask the person to come back to discuss further, perhaps with a family member or friend.

2.3 Self-help groups

Consider advising people with drug dependence to join a self-help group, e.g. Narcotics Anonymous. Consider facilitating initial contact, for example by making the appointment and accompanying the person to the first session.

2.4 Address housing and employment needs

- Where available, work with local agencies and community resources to provide supported employment for those who need support to return to work or find a job and to enable access to local employment (or educational) opportunities, based on the person's needs and skill level.
- Where available, work with local agencies and community resources to find supported housing or assisted living facilities, as well as independent living facilities, if these are needed. Carefully consider the capacity of the person and the availability of alcohol or other substances in advising and facilitating optimal housing arrangements.

2.5 Supporting families and carers

Discuss with families and carers the impact of drug use and drug use disorders on themselves and other family members, including children. Based on feedback from families:

- Offer an assessment of their personal, social and mental health needs.
- Provide information and education about drug use and drug use disorders.
- Help to identify sources of stress related to drug use; explore methods of coping and promote effective coping behaviours.
- Inform them about and help them access support groups (e.g. Self-help groups or families and carers) and other social resources.

Drug Use and Drug Use Disorders

Intervention Details

Psychosocial Interventions

2.6 Harm-reduction strategies

- Advise on the risks of drug injection.
- Provide information on less risky injection techniques and the importance of using sterile injection equipment.
- Give information on how to access needle and syringe exchange programs where they exist, or other sources of sterile injection equipment.
- Encourage and offer testing for blood-borne viral illnesses, whenever possible.
- Offer treatment for complications of drug use and other medical and psychiatric problems, and psychosocial support, even if the person does not wish to cease using drugs at this time.
- Over time, when a relationship has been established, intensified efforts should be made to encourage people who inject drugs to receive treatment for their drug use disorders.

2.7 Women – pregnancy and breastfeeding

- Inquire about the menstrual cycle and inform women that drug use can interfere with the menstrual cycle, sometimes creating the false impression that pregnancy is not possible.
- Advise and support women who are pregnant to stop all psychoactive drug use. Pregnant opioid dependent women should generally be advised to take an opioid agonist replacement such as methadone.
- Screen babies of mothers with drug use disorders for withdrawal symptoms (also known as neonatal abstinence syndrome). Neonatal abstinence syndrome due to maternal opioid use should be treated with low doses of opioids (such as morphine) or barbiturates.
- Advise and support breastfeeding mothers not to use any psychoactive drugs.
- Advise and support mothers with substance use disorders to breastfeed exclusively for at least the first 6 months, unless there is specialist advice not to breastfeed.
- Mothers with harmful substance use and young children should be offered what social support services are available, including additional post natal visits, parenting training, and child care during medical visits.

Intervention Details

Pharmacotherapy

3.1 Managing opioid withdrawal

! Caution is needed before embarking upon withdrawal from opioids, especially where there has been injection use. Withdrawal results in lowered tolerance to opioids, and if there is resumption of opioid use, the person will be at risk of overdose. Withdrawal is best undertaken when there is a plan for admission to a residential rehabilitation programme. Alternatively, the person may be considered for opioid agonist maintenance treatment with either methadone or buprenorphine. When a decision is made to initiate withdrawal, inform about the expected symptoms and duration of the withdrawal process, and select one of the following:

» **Buprenorphine** Buprenorphine is given sublingually at a dose range of 4 – 16 mg per day for 3 to 14 days. Before initiation of buprenorphine treatment, it is important to wait until opioid withdrawal symptoms and signs have become evident (at least 8 hours after the last dose of heroin and at least 24 – 48 hours after the last dose of methadone) because there is a risk that it will precipitate a withdrawal syndrome. Care should be taken particularly if the person is prescribed other sedative drugs.

» **Methadon**e is given orally at an initial dose of 15 – 20 mg, increasing if necessary to 30 mg per day, and then tapering off over 3 to 10 days. Care should be taken particularly if the person is prescribed other sedative drugs.

» **Clonidine or lofexidine** is given at a dose range of 0.1 – 0.15 mg 3 times daily (according to body weight). Lightheadedness and sedation may result. Symptomatic treatment should be given, e.g. treat nausea with anti-emetics, pain with simple analgesics, and insomnia with light sedatives. Monitor blood pressure closely.

3.2 Managing benzodiazepine withdrawal

Elective withdrawal from benzodiazepines utilizes a gradual taper in dose over 8 – 12 weeks and with conversion to long-acting benzodiazepines; in conjunction with psychosocial support.

If severe uncontrolled benzodiazepine withdrawal develops (or occurs in an unplanned way on sudden cessation of these drugs): **CONSULT SPECIALIST** or other available resource person regarding starting a high-dose benzodiazepine sedation regime and hospitalisation.

! *Avoid prescribing benzodiazepines to unknown outpatients.*

3.3 Other drug withdrawal (amphetamines, cannabis, cocaine)

» Manage withdrawal symptoms as they emerge, e.g. Treat nausea with anti-emetics, pain with simple analgesics, insomnia with light sedatives.

» Maintain hydration.

» Avoid restraining the person.

» Allow the person to leave the treatment facility if they wish to do so.

» Depressive symptoms may occur after withdrawal or during the withdrawal period and the person may have pre-existing depression. **BE ALERT TO THE RISK OF SUICIDE.**

3.4 Continued treatment and support after detoxification

Offer all people continued treatment, support and monitoring after successful detoxification, irrespective of the setting in which it was delivered.

3.5 Opioid agonist maintenance treatment (also known as opioid substitution treatment)

Opioid agonist maintenance requires the presence of established and regulated framework (these medications should not be prescribed in the absence of such framework). It is characterized by the prescription of long-acting opioids, such as methadone and buprenorphine, generally on a daily supervised basis. There is strong evidence that agonist maintenance treatment with methadone and buprenorphine effectively reduces illicit drug use, mortality, spread of HIV and criminality, and improves physical and mental health and social functioning.

Monitoring: Medications used in opioid agonist maintenance treatment are open to misuse and diversion; hence, programmes use various methods of limiting the risk of diversion, including supervised consumption.

Self-harm / Suicide

Suicide is the act of deliberately killing oneself. Self-harm is a broader term referring to intentional self-inflicted poisoning or injury, which may or may not have a fatal intent or outcome. Any person over 10 years of age experiencing any of the following conditions should be asked about thoughts or plans of self-harm in the last month and about acts of self-harm in the last year:

» any of the other priority conditions (see mhGAP-IG Master Chart);
» chronic pain;
» acute emotional distress.

Evaluate thoughts, plans and acts of self-harm during the initial evaluation and periodically thereafter as required. Attend to the person's mental state and emotional distress.

!

Asking about self-harm does NOT provoke acts of self-harm. It often reduces anxiety associated with thoughts or acts of self harm and helps the person feel understood.
However, try to establish a relationship with the person before asking questions about self-harm. Ask the person to explain their reasons for harming themselves.

Self - harm / Suicide

Assessment and Management Guide

1. Has the person attempted a medically Serious act of self-harm?

Observe for evidence of self-injury

Look for:

- Signs of poisoning or intoxication
- Signs / symptoms requiring urgent medical treatment such as:
  - bleeding from self-inflicted wound
  - loss of consciousness
  - extreme lethargy

Ask about:

- Recent poisoning or other self-harm

YES

If person requires urgent medical treatment for act of Self-harm

- Medically treat injury or poisoning.
- If Acute Pesticide Intoxication, follow Pesticide Intoxication Management. » **SUI 2.3**
- If medical hospitalization is needed, continue to monitor the person closely to prevent suicide

NO

If NO, assess for imminent risk of self-harm / suicide

In all cases:

Place the person in a secure and supportive environment at the health facility while being assessed (do not leave them alone).

- Care for the person with self-harm. » SUI 2.1
- Offer and activate psychosocial support. » SUI 2.2
- Consult mental health specialist if available.
- Maintain regular contact and follow-up. » SUI 2.4

Self - harm / Suicide

SUI 1

Assessment and Management Guide

2. Is there an imminent risk of Self-harm / suicide?

Ask person and carer about:

- Current thoughts or plan to commit suicide or self-harm
- History of thoughts or plan of self-harm in the past month or act of self-harm in the past year
- Access to means of self-harm

Look for:

- Severe emotional distress
- Hopelessness
- Extreme agitation
- Violence
- Uncommunicative behaviour
- Social isolation

YES

If there are:

- current thoughts or plan to commit suicide / self-harm

OR

- history of thoughts or plan of self-harm in the past month or act of self-harm in the past year in a person who is now extremely **agitated, violent, distressed** or **uncommunicative**

There is **imminent risk of self-harm / suicide.**

- **Take the following precautions:**
  - Remove means of self-harm.
  - Create secure and supportive environment; if possible, offer separate, quiet room while waiting.
  - Do not leave the person alone.
  - Supervise and assign a named staff member or a family member to ensure safety.
  - Attend to mental state and emotional distress.

- Offer and activate psychosocial support. **» SUI 2.2**
- Consult mental health specialist, if available.
- Maintain regular contact and follow-up. » SUI 2.4

NO

If there is no imminent risk of self-harm / suicide, **but history of thoughts or plan of self-harm in the past month or act of self-harm in the past year**

- Offer and activate psychosocial support. » SUI 2.2
- Consult mental health specialist, if available.
- Maintain regular contact and follow-up. » SUI 2.4

3. Does the person have concurrent priority mental, neurological or drug use disorders?
(See mhGAP-IG Master Chart)

- Depression
- Alcohol or drug use disorders
- Bipolar disorder
- Psychosis
- Epilepsy
- Behavioural disorders

YES

If concurrent priority Conditions

Manage the concurrent conditions (see relevant modules) in conjunction with the above actions.

4. Does the person have chronic pain?

YES

If chronic pain is present

Manage pain and treat any relevant medical disease.

5. Does the person have emotional symptoms severe enough to warrant Clinical management?

- Difficulty carrying out usual work, school, domestic or social activities
- Marked distress or repeated help-seeking
- Repeated self-medication for emotional distress or unexplained somatic symptoms

YES

If YES, additional clinical management of symptoms is warranted

See the module on **Other Significant Emotional or Medically Unexplained Complaints. » OTH**

Self - harm / Suicide

SUI 2

Intervention Details

Advice and Treatment

2.1 Care for the person with self-harm

Place the person in a secure and supportive environment at the health facility (do not leave them alone). If a person with self-harm must wait for treatment, offer an environment that minimizes distress, if possible in a separate, quiet room with supervision and regular contact with a named staff member or a family member to ensure safety.

- Remove the means of self-harm.
- Consult a mental health specialist, if available.
- Mobilize family, friends and other concerned individuals or available community resources to monitor and support the individual during imminent risk period. » SUI 2.2
- Treat people who have self-harmed with the same care, respect and privacy given to other people, and be sensitive to likely emotional distress associated with self-harm.
- Include the carer(s) if the person wants their support during assessment and treatment, although the psychosocial assessment should usually include a one-to-one interview between the person and health worker to help explore private concerns or issues.
- Provide emotional support to relatives / carers if they need it.
- Ensure continuity of care.
- Hospitalization in non-psychiatric services of general hospitals with the goal of preventing acts of self-harm is not recommended. If admission to a general (non-psychiatric) hospital for management of medical consequences of an act of self-harm is necessary, monitor the person closely to prevent subsequent self-harm in the hospital.
- **If prescribing medication:**
  - use medicines that are the least dangerous in case of overdose;
  - give prescriptions for short duration (e.g. one week at a time).

2.2 Offer and activate psychosocial support

Offer psychosocial support

- Offer support to the person.
- Explore reasons and ways to stay alive.
- Focus on the person's positive strengths by getting them to talk of how earlier problems have been resolved.
- Consider problem-solving therapy for treating people with acts of self-harm in the last year, if sufficient human resources are available. » INT

Activate psychosocial support

- Mobilize family, friends, concerned individuals and other available resources to ensure close monitoring of the individual as long as the risk persists.
- Advise the person and carer(s) to restrict access to the means of self-harm (e.g. pesticides and other toxic substances, medication, firearms) while the individual has thoughts, plans or acts of self-harm.
- Optimize social support from available community resources. These include informal resources such as relatives, friends, acquaintances, colleagues and religious leaders, or formal community resources, if available, such as crisis centres and local mental health centres.
- Inform carers and other family members that asking about suicide will often reduce the anxiety surrounding the feeling; the person may feel relieved and better understood.
- Carers of people at risk of self-harm often experience severe stress. Provide emotional support to relatives / carers if they need it.
- Inform carers that even though they may feel frustrated with the person, it is suggested to avoid hostility or severe criticism towards the person at risk of self-harm.

Intervention Details

Advice and Treatment

2.3 Pesticide Intoxication Management

» If health-care facility has a minimum set of skills and resources, then treat using the WHO document Clinical Management of Acute Pesticide Intoxication (http://www.who.int/mental_health/prevention/suicide/pesticides_intoxication.pdf).

Otherwise, transfer the person immediately to a facility that has the following resources:

- skills and knowledge about how to resuscitate individuals and assess for clinical features of pesticide poisoning;
- skills and knowledge to manage the airway, in particular to intubate and support breathing until a ventilator can be attached;
- atropine and means for its intravenous (i.v.) administration if signs of cholinergic poisoning develop;
- diazepam and means for its i.v. administration if the person develops seizures.

» Consider giving activated charcoal if the person is conscious, gives informed consent and presents within one hour of the poisoning.

» **Forced emesis is not recommended.**

» **Oral fluids should not be given.**

2.4 Maintain regular contact and follow-up

» Maintain regular contact (via telephone, home visits, letters or contact cards), more frequently initially (e.g. weekly for the initial 2 months) and less frequently as the person improves (once in 2 – 4 weeks thereafter). Consider maintaining more intensive or longer contact if necessary.

» Follow up for as long as suicide risk persists. At every contact, routinely assess suicide thoughts and plans. If risk is imminent, go to the subsection Imminent Risk of Self-harm / Suicide in the Assessment and Management section of this module. » SUI 1

2.5 Prevention of suicide

Beyond clinical assessment and management of priority conditions, district-level health officers and health-care providers can take action to prevent suicide, as follows:

» **Restrict access to means of self-harm (such as pesticides, firearms, high places).**

- Actively involve the community to find locally feasible ways to implement interventions at the population level to reduce access to means of suicide.
- Establish collaboration between health and other relevant sectors.

» **Develop policies to reduce harmful use of alcohol as a component of suicide prevention** particularly within populations with high prevalence of alcohol use.

» **Assist and encourage the media to follow responsible reporting practices of suicide.**

- Avoid language which sensationalizes or normalizes suicide or presents it as a solution to a problem.
- Avoid pictures and explicit description of the method used.
- Provide information about where to seek help.

Other Significant Emotional or Medically Unexplained Complaints

OTH

People in the mhGAP-IG category "Other Significant Emotional or Medically Unexplained Complaints" have anxiety, depressive or medically unexplained somatic symptoms. They do not have any of the conditions covered elsewhere in this document (except possibly for the condition self-harm). People in this category may experience either "normal" distress or a mental disorder not covered in the mhGAP-IG (e.g. somatoform disorder, mild depression, dysthymia, panic disorder, generalized anxiety disorder, post-traumatic stress disorder, acute stress reaction, adjustment disorder).

The management of "Other Significant Emotional or Medically Unexplained Complaints" by practitioners trained in mhGAP-IG excludes psychotropic medications. Nonetheless, a subset of people in this category may benefit from medication prescribed by a qualified practitioner trained in diagnosis and evidence-based treatment of conditions not covered in this Intervention Guide.

!

» This module should not be considered for people who meet the criteria for any of the mhGAP priority conditions (except for the condition self-harm).

» This module should only be used after explicitly ruling out moderate-severe depression **(» DEP).**

Assessment and Management Guide

1. Does the person have moderate-severe depression or any other priority Condition (other than self-harm)?

YES — If YES

» Terminate assessment and go to relevant module.

2. Does the person have a physical condition that fully explains the Presence of the symptoms?

YES — If YES

» Terminate assessment.
» Initiate relevant medical treatment and follow-up.

» Conduct a general medical examination and essential Investigations

NO — If NO

In ALL cases:

» **DO NOT** prescribe antidepressants or benzodiazepines
» **DO NOT** manage complaints with injections or other ineffective treatments (e.g. vitamins).
» Address current psychosocial stressors. **» DEP 2.2**
» In adolescents and adults:
  – Address inappropriate self-medication.
  – Reactivate social networks. **» DEP 2.3**
  – Where available, consider one of the following treatments: structured physical activity programme, **» DEP 2.4** behavioural activation, relaxation training, or problem-solving treatment. **» INT**
» Follow up. **CONSULT A SPECIALIST** if no improvement at all or If the person (or his / her parents) asks for more intense treatment.

Other Significant Emotional or Medically Unexplained Complaints

OTH 1

Assessment and Management Guide

3. Are there prominent medically Unexplained somatic symptoms?

YES

If YES

Follow above advice (applicable to ALL cases) plus:

- » Avoid unnecessary medical tests / referrals and do not offer placebo.
- » Acknowledge that the symptoms are not "imaginary".
- » Communicate results of tests / examination, saying that no dangerous disease has been identi? ed, but that it is nevertheless important to deal with the distressing symptoms.
- » Ask for the person's explanations of somatic symptoms.
- » Explain how bodily sensations (stomach ache, muscle tension) can be related to experiencing emotions, and ask for potential links between the person's bodily sensations and emotions.
- » Encourage continuation of (or gradual return to) normal activities.
- » Advise the person to re-consult if symptoms worsen.

4. Has the person been recently exposed to extreme stressors (losses, traumatic Events)?

YES

If YES

Follow above advice (applicable to ALL cases) plus:

- » In case of bereavement: support culturally appropriate mourning / adjustment and reactivate social networks. **» DEP 2.3**
- » In case of acute distress after recent traumatic events: offer basic psychological support (psychological ? rst-aid), i.e., listen without pressing the person to talk; assess needs and concerns; ensure basic physical needs are met; provide or mobilize social support and protect from further harm.
- » **DO NOT** offer psychological debrie? ng (i.e., do not promote ventilation by requesting a person to brie? y but systematically recount perceptions, thoughts, and emotional reactions experienced during a recent, stressful event).

5. Have there been (a) thoughts or plans of suicide / self-harm during the last Month or (b) acts of self-harm during The last year?

YES

If YES

- » Manage both the signi? cant emotional or medically unexplained Complaints (see above) and the risk of self-harm. **» SUI 1**

Advanced Psychosocial Interventions

INT

For the purposes of the mhGAP-IG, the term “advanced psychosocial intervention” refers to an intervention that takes more than a few hours of a health-care provider’s time to learn and typically more than a few hours to implement. Such interventions can be implemented in non-specialized care settings, but only when sufficient human resource time is made available.

Advanced Psychosocial Interventions

INT

The interventions described in this section cover both psychological and social interventions requiring substantial dedicated time. A number of the described interventions are known as psychotherapies or psychological treatments. Around the world, these treatments tend to be provided by specialists specifically trained in them. Nonetheless, they may be offered by trained and supervised non-specialized health workers. These psychological treatments are usually provided on a weekly basis over a number of months in either individual or group format.

Some of the interventions, such as cognitive behavioural therapy and interpersonal psychotherapy, have successfully been implemented by community health workers in low-income countries as part of research programmes that ensured that community health workers had the time to learn and implement these interventions under supervision. These examples show that these interventions can be made available through non-specialized human resources, opening-up possibilities for scaling up.

Scaling up care requires investment. Health-system managers should aim to allocate sufficient human resources to care for mental, neurological and substance use disorders in order to ensure the wide availability of the interventions covered in this section.

The remainder of this section provides summary descriptions of each of the interventions (in alphabetical order). Within the modules, these interventions are marked by the abbreviation **» INT,** indicating that these require a relatively more intensive use of human resources. There is a need to develop specific protocols and training manuals for implementing these interventions in non-specialized health-care settings.

Behavioural activation

Behavioural activation, which is also a component of Cognitive behavioural therapy for depression, is a psychological treatment that focuses on activity scheduling to encourage a person to stop avoiding activities that are rewarding. The mhGAP-IG recommends it as a treatment option for depression (including bipolar depression) and other significant emotional or medically unexplained complaints.

Cognitive behavioural therapy (CBT)

Cognitive behavioural therapy (CBT) is based on the idea that feelings are affected by thinking and behaviour. People with mental disorder tend to have unrealistic distorted thoughts, which if unchecked can lead to unhelpful behaviour. CBT typically has a cognitive component (helping the person develop the ability to identify and challenge unrealistic negative thoughts) and a behavioural component. CBT is different for different mental health problems. The mhGAP-IG recommends it as a treatment option for depression (including bipolar depression), behavioural disorders, alcohol use disorders or drug use disorders, and also recommends it as a treatment option for psychosis just after the acute phase.

Contingency management therapy

Contingency management therapy is a structured method of rewarding certain desired behaviours, such as attending treatment, behaving appropriately in treatment and avoiding harmful substance use. Rewards for desired behaviours are reduced over time as the natural rewards become established. The mhGAP-IG recommends it as a therapy for people with alcohol use disorders or drug use disorders.

Family counselling or therapy

Family counselling or therapy should include the person if feasible. It entails multiple (usually more than six) planned sessions over a period of months. It should be delivered to individual families or groups of families. It has supportive and educational or treatment functions. It often includes negotiated problem-solving or crisis management work. The mhGAP-IG recommends it as a therapy for people with psychosis, alcohol use disorders or drug use disorders.

Interpersonal psychotherapy (IPT)

Interpersonal psychotherapy (IPT) is a psychological treatment designed to help a person identify and address problems in their relationships with family, friends, partners and other people. The mhGAP-IG recommends it as a treatment option for depression, including bipolar depression.

Motivational enhancement therapy

Motivational enhancement therapy is a structured therapy, typically lasting four sessions or less, to help people who are dependent on substances. It involves an approach to motivate change by using the motivational interviewing techniques described in the section on brief interventions. » ALC 2.2 The mhGAP-IG recommends it as therapy for people with alcohol use disorders or drug use disorders.

Advanced Psychosocial Interventions

Parent skills training for parents of children and adolescents with behavioural disorders

Parent skills training for parents of children with behavioural disorders involves training focusing on positive parent-child interactions and emotional communication, teaching the importance of parenting consistency, discouraging harsh punishments and requiring the practice of new skills with their children during the training. Although the content should be culturally sensitive, it should not allow violation of children's basic human rights according to internationally endorsed principles. Providing parent training requires that the health-care providers receive training themselves.

Parent skills training for parents of children and adolescents with developmental disorders

Parent skills training for parents of children with developmental disorders involves using culturally appropriate training material relevant to the problem to improve development, functioning and participation of the child within families and communities. It involves techniques teaching specific social, communicative and behavioural skills using behavioural principles (e.g. Teaching new behaviours by rewarding these behaviours, or addressing problem behaviours by carefully analysing triggers of the problem behaviour) to change contributing environmental factors. Parents need to be supported in the application of the training. Parents of children with different levels of intellectual disability and specific problem behaviours need to develop additional skills adapted to the needs of their children. Health-care providers need additional training to be able to offer parent training.

Problem-solving counselling or therapy

Problem-solving counselling or therapy is a psychological treatment involving offering direct and practical support. The therapist and person work together to identify and isolate key problem areas that might be contributing to the person's mental health problems, to break these down into specific, manageable tasks, and to problem-solve and develop coping strategies for particular problems. The mhGAP-IG recommends it as an adjunct treatment option for depression (including bipolar depression) and as a treatment option for alcohol use disorders or drug use disorders. It is also recommended for self-harm, other significant emotional or medically unexplained complaints, or parents of children and adolescents with behavioural disorders.

Relaxation training

This intervention involves training the person in techniques such as breathing exercises and progressive relaxation to elicit the relaxation response. Progressive relaxation teaches how to identify and relax specific muscle groups. Usually treatment consists of daily relaxation exercises for at least 1 – 2 months. The mhGAP- IG recommends it as an adjunct treatment option for depression (including bipolar depression), and as a treatment option for other significant emotional or medically unexplained complaints.

Social skills therapy

Social skills therapy helps rebuild skills and coping in social situations to reduce distress in everyday life. It uses role-playing, social tasks, encouragement and positive social reinforcement to help improve ability in communication and social interactions. Skills training can be done with individuals, families and groups. Usually treatment consists of 45 to 90 minute sessions once or twice per week for an initial 3 months and then monthly later. The mhGAP-IG recommends it as a treatment option for people with psychosis or behavioural disorder.

A Partnership between the Ministry of Health of Nigeria,
World Health Organization and European Commission

For more information, please contact:

World Health Organization Country Office,
Plot 1620A, Maitama Sule Street,
Off Yakubu Gowon Crescent [illegible]
